# Bioinformatic characterization of type-specific sequence and structural features in auxiliary activity family 9 proteins

**DOI:** 10.1186/s13068-016-0655-2

**Published:** 2016-11-09

**Authors:** Vuyani Moses, Rowan Hatherley, Özlem Tastan Bishop

**Affiliations:** Research Unit in Bioinformatics (RUBi), Department of Biochemistry and Microbiology, Rhodes University, Grahamstown, 6140 South Africa

**Keywords:** AA9, LPMO, PMO, Motifs, Phylogenetics, Structural analysis

## Abstract

**Background:**

Due to the impending depletion of fossil fuels, it has become important to identify alternative energy sources. The biofuel industry has proven to be a promising alternative. However, owing to the complex nature of plant biomass, hence the degradation, biofuel production remains a challenge. The copper-dependent Auxiliary Activity family 9 (AA9) proteins have been found to act synergistically with other cellulose-degrading enzymes resulting in an increased rate of cellulose breakdown. AA9 proteins are lytic polysaccharide monooxygenase (LPMO) enzymes, otherwise known as polysaccharide monooxygenases (PMOs). They are further classified as Type 1, 2 or 3 PMOs, depending on the different cleavage products formed. As AA9 proteins are known to exhibit low sequence conservation, the analysis of unique features of AA9 domains of these enzymes should provide insights for the better understanding of how different AA9 PMO types function.

**Results:**

Bioinformatics approaches were used to identify features specific to the catalytic AA9 domains of each type of AA9 PMO. Sequence analysis showed the N terminus to be highly variable with type-specific inserts evident in this region. Phylogenetic analysis was performed to cluster AA9 domains based on their types. Motif analysis enabled the identification of sub-groups within each AA9 PMO type with the majority of these motifs occurring within the highly variable N terminus of AA9 domains. AA9 domain structures were manually docked to crystalline cellulose and used to analyze both the type-specific inserts and motifs at a structural level. The results indicated that these regions influence the AA9 domain active site topology and may contribute to the regioselectivity displayed by different AA9 PMO types. Physicochemical property analysis was performed and detected significant differences in aromaticity, isoelectric point and instability index between certain AA9 PMO types.

**Conclusions:**

In this study, a type-specific characterisation of AA9 domains was performed using various bioinformatics approaches. These highly variable proteins were found to have a greater degree of conservation within their respective types. Type-specific features were identified for AA9 domains, which could be observed at a sequence, structural and physicochemical level. This provides a basis under which to identify and group new AA9 LPMOs in future.

**Electronic supplementary material:**

The online version of this article (doi:10.1186/s13068-016-0655-2) contains supplementary material, which is available to authorized users.

## Background

Plant biomass is a material with high structural and chemical complexity providing huge potential for biotechnological applications [1]. Biomass is regarded as one of the world’s major sources of energy. Currently, it is believed that biomass contributes roughly around 10–14% of the world’s energy supply [2]. Various approaches have been proposed for breaking down plant biomass with the most common being biochemical conversion and thermochemical conversion. Biochemical conversion is often seen as an advantageous approach over thermochemical conversion as it does not result in the destruction of carbohydrate structures [3]. There are many factors that influence biomass recalcitrance; however, the main contributor is its major constituent, cellulose.

Cellulose occurs as a linear homopolymer, composed of glucose molecules that are arranged in a repeating fashion joined by beta-1,4-glucosidic bonds. Enzyme systems have been developed in various organisms for the cellulose degradation. The initial stage in the degradation of lignocellulosic matter has always been a crucial step for the entire process. Fungal organisms employ a wide range of cellulose-degrading enzymes which work in concert to degrade cellulose [4]. A group of enzymes, called lytic polysaccharide monooxygenases (LPMOs), also referred to as polysaccharide monooxygenases (PMOs), has recently received a great deal of research attention for their potential application in cellulose degradation [5]. LPMOs are divided into three groups: Auxiliary Activity (AA) family 9, AA10, AA11 and AA13 [6, 7].

AA9 enzymes, previously characterized as glycohydrolase 61 (GH61), are copper-containing metallo enzymes. The type II copper ion is coordinated by a histidine brace, characteristic of these enzymes [8, 9]. The inclusion of AA9 enzymes in reaction mixtures has been demonstrated to have synergistic effects with other cellulose-degrading enzymes resulting in an increased rate of cellulose degradation [10]. This increase in cellulose degradation is largely attributed to AA9 proteins direct interaction with crystalline cellulose through their flat active site surface. It has been proposed that AA9 proteins introduce nicks on crystalline cellulose making it easily accessible for degradation by classical cellulose-degrading enzymes [11]. AA9 proteins have been suggested to oxidatively cleave the glycosidic linkages connecting cellulose without removing the glucan chain from the surface of cellulose [12].

AA9 enzymes have been categorized into three distinct types depending on their ability to oxidize the cellulose structure at different cellulose carbon positions [8, 13]. They are referred to Type 1, 2 and 3 PMOs (also PMO1, PMO2 and PMO3, respectively), although this nomenclature has so far only been applied to AA9 LPMOs [5, 6, 12–16]. The cleavage at either C1 or C4 produces aldonolactone or 4-ketoaldose, respectively [12, 16]. Type 1 PMOs cleave the C1 carbon of pyranose residues, Type 2 PMOs are known to cleave the C4 carbon of pyranose residues and Type 3 PMOs are unspecific in their preference for producing either aldonolactone or 4-ketoaldose [9, 12, 13, 16]. There is some evidence suggesting that AA9 proteins can also oxidize the C6 carbons of the glucose ring [13].

AA9 LPMOs are modular proteins which contain a catalytic AA9 domain, often coupled with different carbohydrate-binding modules (CBMs) [8]. A common feature of the AA9 domain is the lack of a substrate-binding pocket [17]. The absence of a classical binding site led to the reclassification of these enzymes as auxiliary activity (AA) enzymes as opposed to glycoside hydrolases (GHs) [8]. A characteristic feature of AA9 domains is a conserved immunoglobulin like beta-sandwich fold. The beta strands form a conserved scaffold, which is linked by highly variable loop regions [17]. Studies seeking to describe the contributors of substrate specificity and regioselectivity have been carried out [18, 19]. One such study [18] showed that AA9 LPMOs are a highly diverse protein group with this diversity affecting the flat active site surface extensively. Further investigation has been successful in identifying of regions on the surface of AA9 proteins that may play a role in regioselectivity of different AA9 PMO types [19]. These studies are consistent with the proposal put forward by Hemsworth et al. [6], which suggests that the loop regions surrounding copper ion in the active site of LPMOs may affect substrate binding and orientation which results in the observed regioselectivity.

AA9 proteins are renowned for their diversity and abundance in fungal genomes. It is a well-known fact that many fungal organisms encode multiple AA9 proteins [13]. Even though AA9 proteins have demonstrated high sequence variability, the presence of distinct AA9 PMO types alludes to the presence of unique type-specific features. To further understand AA9 protein sequence diversity and its effect on AA9 PMO type specificity, it is important to characterize and quantify unique sequence and structural features to determine their potential impact on regioselectivity of AA9 PMO types. The aim of the study was to analyze type-specific sequence and structural features of AA9 proteins using various bioinformatics approaches. The high sequence diversity inherent to AA9 proteins may have functional consequences, since primary protein sequence dictates the structure of proteins which in turn relates to the function [18]. Identification of type-specific features would further contribute to the classification of new AA9 LPMOs, and highlight regions of these proteins that may be further studied to gain insight into how they function. Here, we present the bioinformatic characterization of AA9 domains from 153 different AA9 proteins. We identify a number of sequence and structural features, as well as physicochemical properties that differentiate between different types of AA9 PMOs.

## Methods

### Data retrieval

AA9 protein sequences were retrieved from the Pfam 27.0 database [20], Pfam id: PF03443. The collection consisted of 827 modular protein sequences which all had AA9 domains. These sequences were from 87 fungal organisms and one plant organism (*Zea Mays*). The multiple alignment using fast Fourier transform (MAFFT) alignment tool [21] was used for the initial alignment of sequences. This alignment revealed the presence of highly variable sequences and short fragments in the dataset, which were subsequently removed. This was done by extracting the AA9 domains from each sequence in the alignment. Once the AA9 domain had been extracted, a python script was used to remove duplicate sequences (100% sequence identity), resulting in 139 AA9 protein domains included in this study. The sequences from *Neurospora crassa* were used as references for each AA9 PMO type (Table 1). These sequences had been previously classified as Type 1, 2 or 3 PMOs [13]. The remaining 139 sequences used in this study are shown in Additional file 1. Further, all the available AA9 crystal structures were obtained from the Protein Data Bank (PDB) and are presented in Table 1. The final dataset included 153 AA9 domain sequences, some of which also contained a signal peptide region.

**Table 1:** Available AA9 PDB structures and *Neurospora crassa* reference sequences

PDB ID	Uniprot accession	Organism	AA9 PMO type	Citation
PDB sequences				
4B5Q	H1AE14	*Phanerochaete chrysosporium*	Type 1	[39]
3EII	D0VWZ9	*Thielavia terrestris*	Type 1	[37]
3EJA	D0VWZ9	*Thielavia terrestris*	Type 1	[37]
4EIS	Q7SA19	*Neurospora crassa*	Type 1	[13]
4EIR	Q1K8B6	*Neurospora crassa*	Type 2	[13]
2YET	G3XAP7	*Thermoascus aurantiacus*	Type 3	[9]
3ZUD	G3XAP7	*Thermoascus aurantiacus*	Type 3	[9]
2VTC	GUN7	*Trichoderma reesei*	Type 3	[38]
Protein				
*Neurospora crassa* reference sequences				
NCU00836	Q7SCJ5	*Neurospora crassa*	Type 1	[13]
NCU03328	Q1K4Q1	*Neurospora crassa*	Type 1	[13]
NCU02344	Q7S411	*Neurospora crassa*	Type 1	[13]
NCU01050	Q1K8B6	*Neurospora crassa*	Type 2	[13]
NCU02240	Q7S439	*Neurospora crassa*	Type 2	[13]
NCU02916	Q7SHI8	*Neurospora crassa*	Type 2	[13]
NCU07898	Q7SA19	*Neurospora crassa*	Type 3	[13]
NCU05969	Q7S1V2	*Neurospora crassa*	Type 3	[13]
NCU07760	Q7S111	*Neurospora crassa*	Type 3	[13]

### Sequence alignment

The PROfile Multiple Alignment with predicted Local Structures and 3D constraints (PROMALS3D) alignment tool [22] was, then, used to generate a more accurate AA9 alignment by including structural information. The crystal structures listed in Table 1 were used as input for the alignment program. Through phylogenetic clustering and comparing sequence identity with their respective reference sequences, the sequences in the dataset were divided into three types. These groupings were, then, used to generate type-specific PROMALS3D alignments. The input structures used for these alignments were 4B5Q, 4EIR and 3ZUD for Type 1, 2 and 3, respectively. Once all alignments were carried out, again all vs all sequence identity calculations were done using Matlab to identify the extent variation in AA9 domain sequences.

### Phylogenetic analysis

To conduct further type-specific analyses on the AA9 domain, it was crucial to cluster the sequences by phylogenetic tree calculations. Phylogenetic trees were constructed using molecular evolutionary genetic analysis (MEGA) v6.0 [23]. Bayesian information criterion (BIC) scores were used to determine the best evolutionary model for phylogenetic tree construction. Evolutionary models with the lowest BIC scores were chosen as the best. Evaluation of models was conducted under three different gap deletions (90, 95 and 100%). Maximum likelihood trees were constructed for the best three models for each gap deletion. For all trees constructed, a maximum heuristics search was conducted using Nearest-Neighbor-Interchange (NNI). The initial trees were generated using the default Neighbor Join and BioNJ algorithms. 1000 bootstrap replicates and a very strong branch swap filter were used with each tree construction. The best models were determined to be Whelan And Goldman model (WAG) [24], WAG and Gama distribution (WAG + G) and WAG + G with Invariant sites (WAG + G+I). Phylogenetic trees where constructed for all three models at the three specified gap deletions resulting in the calculation of nine trees. Each generated phylogenetic tree was compared to its respective bootstrap consensus tree to observe overall branching pattern. The phylogenetic tree of the WAG + G + I model at a 90% gap deletion was chosen as the best tree due to the observed branch support and similar branch pattern to the bootstrap consensus tree.

### Physicochemical property analysis

Type-specific comparative physicochemical property analysis was performed. The separated three AA9 groups were individually analyzed based on aromaticity [25], grand average of hydrophobicity (GRAVY) index, isoelectric points [26], instability index [27], as well as molecular weights and amino acid residue composition. Aromaticity (a relative measure of aromatic residues in a protein sequence) was calculated using the protein analysis class from the ProtParam module in BioPython. The GRAVY index is a hydropathicity index that describes the solubility of the proteins where a protein with a positive GRAVY index is hydrophobic and a protein with a negative GRAVY index is hydrophilic [28]. A *t* test was used to determine any significant differences in the distributions of physicochemical properties observed amongst AA9 PMO types, performed using the R package, at a 5% level of significance.

### Homology modeling

Homology modeling was done for the Type 1 sequence, *Aspergillus niger* (*A. niger*) AA9 homolog 9 (Uniprot accession: G3XUH5.1). The template utilized for modeling (PDB ID: 4B5Q) was identified using the HHpred webserver [29]). Homology modeling was carried out with MODELLERv9.12 [30]. 100 models were generated with refinement set to very slow. DOPE Z-score [31] evaluations were used to rank the models and the top three models were selected for further analysis. The MetaMQAPII [32] webserver was used to select the best model.

### Motif analysis

Motif analysis was done for all sequences using the Multiple Em for Motif Elucidation (MEME) webserver [33] to search for conserved motifs with a size range between 6 and 50 residues. Motif alignment search tool (MAST) [34] was utilized to detect the presence of any overlapping motifs. 30 significant motifs were identified. MEME log file was parsed using a Matlab script to generate a heat map showing the conservation of motifs among AA9 domains. The type-specific motifs observed from this analysis were mapped to respective AA9 domain structures.

### Manual docking and structure mapping

Manual docking of AA9 proteins to crystalline cellulose was performed using a similar method to Li et al. [13]. This was done to identify structurally important features that are crucial for type-specific substrate binding and to observe the interaction of type-specific motifs with cellulose. Surface-exposed aromatic residues, as described by Li et al. [13], were identified in each AA9 crystal structure. The constructed cellulose substrate consisted of 5 chains of 12 pyranose residues using a Iβ asymmetric unit coordinates [35]. Important type-specific features were then mapped on Type 1, 2 and 3 AA9 crystal structures 3EJA, 4EIR and 3ZUD, respectively. For the Type 1 AA9 structure 3EJA was manually docked such that Tyr-190, Tyr-191 and Tyr-67 were aligned to the pyranose residues of cellulose chain 3 while His-1 was aligned to cellulose chain 4. The Type 2 AA9 crystal structure was aligned such that the His-1 and Tyr-206 were aligned with the cellulose chain 3. The Type 3 AA9 crystal structure was aligned such that the His-1, Tyr-24 and Tyr-212 were aligned with the cellulose chain 3. The *A. niger* homology model was aligned to cellulose such that His-1 and Trp-34 was aligned with cellulose chain 4, Trp-131 was aligned with cellulose chain 3 and Trp-207 was aligned with cellulose chain 4.

## Results and discussion

This study focused on identifying features inherent to the different types of AA9 PMOs at a sequence, structural and physicochemical level. This was done by studying the catalytic AA9 domains of these proteins by clustering 153 sequences retrieved from Pfam into their respective types and identifying type-specific features.

### Multiple sequence alignment shows AA9 PMO type-specific inserts

**Fig. 1:**
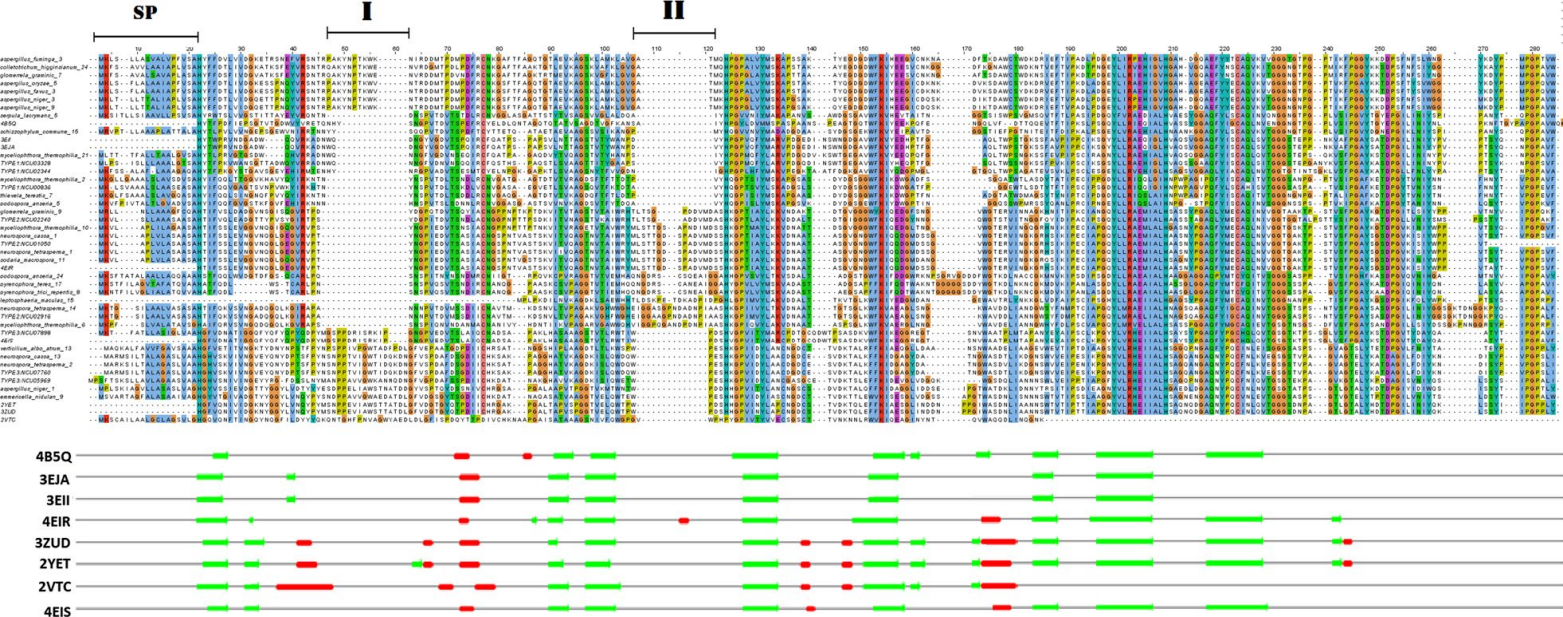
Promals3D alignment of AA9 domains. An alignment of 47 representative sequences to indicate the different AA9 sequences retrieved. The signal peptide (SP) is shown above the sequences, along with insert regions I and II. The PDB IDs of structures used as Promals3D input are shown at the* bottom* along with a representation of secondary structure along their sequences. Alpha helices are shown in *red* and beta sheets are shown in *green*

After extracting 153 AA9 domain sequences, as indicated in the methodology section, the sequences were aligned using PROMALS3D (see Additional file 2). The alignment of AA9 sequences revealed that the N terminus of AA9 domains is highly variable as opposed to the more conserved C terminus. Closer analysis of the variable N terminus showed type-specific insertions and deletions present in this region. Previously, Vu et al. [19] showed that inserts in the N terminus region of AA9 domains play a role in type specificity. Here, we observed that the type-specific inserts were predominantly conserved in their respective sequences. Figure 1 represents a small subset of 47 aligned sequences extracted from the full alignment in Additional file 2. In the alignment in Fig. 1, three distinct sequence groups are apparent. These sequence groups are characterized by the presence or absence of inserts at specific positions in the alignment, which we refer to as regions I and II. The first group of sequences was found to lack both inserts in regions I and II. The second AA9 group only possessed inserts in region II. The third AA9 group possessed only inserts in region I. Interestingly, the AA9 sequences containing an eight-residue insert in region I (Fig. 1) were found to be Type 1 PMOs. Phylogenetic analysis (described later) confirmed that these clustered with other Type 1 PMO sequences.

**Fig. 2:**
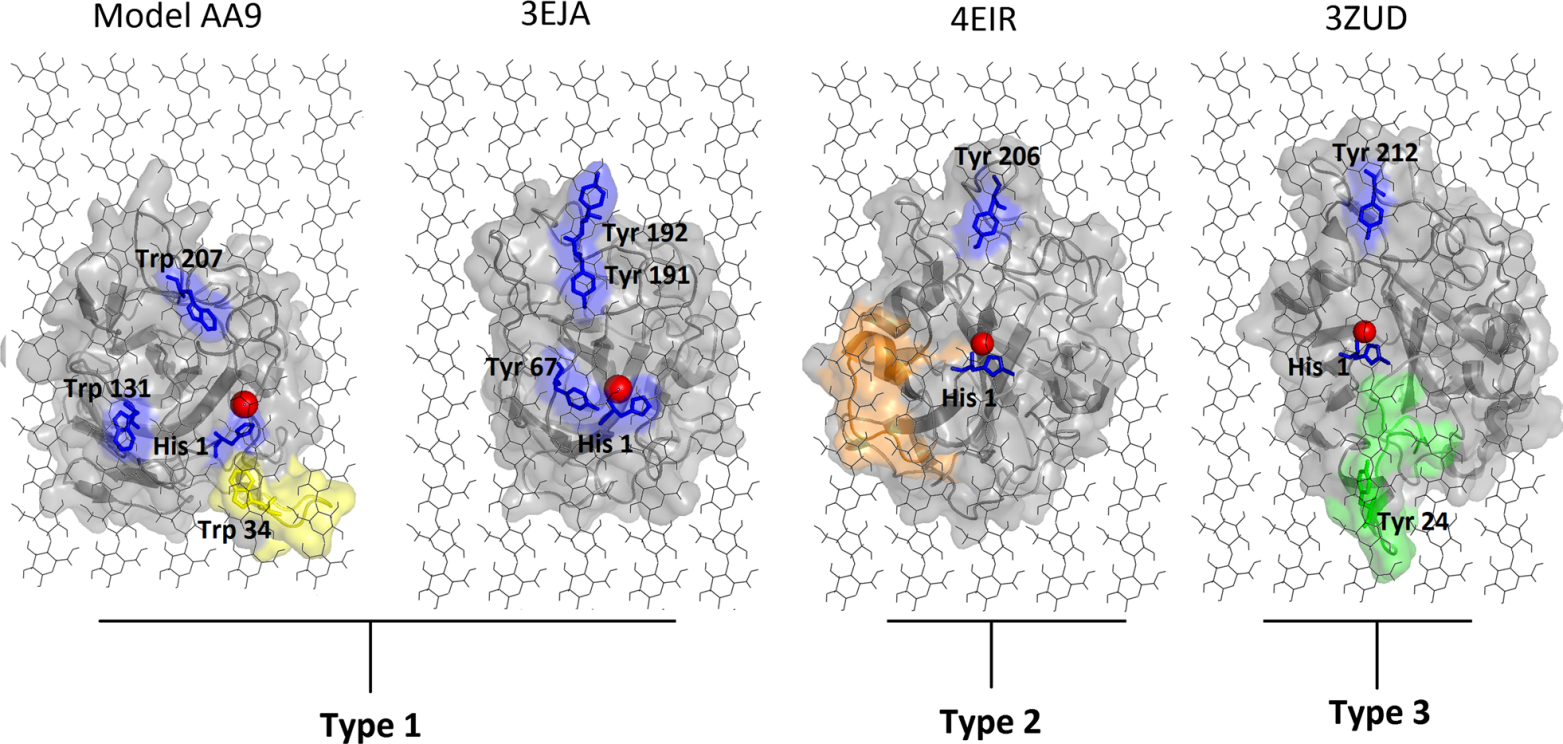
Structural representation of type-specific inserts of AA9 domains. Proteins are shown as cartoons and colored in *gray*, with the inserts colored in *green* and *orange* for region I and II, respectively. The Type 1 region I insert is colored in *yellow*. The AA9 structures were aligned onto cellulose Iβ using the planar aromatic residues on the AA9 active site. The cellulose is shown in *black lines* and the flat surface aromatic residues are represented in sticks and are colored in *blue*, with the type II copper ion shown as a *red sphere*. The planar residues Trp-34 on the model AA9 is colored in *yellow* as it forms part of the region I insert. On the 3ZUD crystal structure the Tyr 24 residue is colored in *green* as it forms part of the region I insert

As previous studies have shown [19], protein structures with region I inserts (Fig. 1) were found to possess a modification on the active site surface that is characteristic of Type 3 PMO sequences. A similar observation was made for Type 2 PMOs as they were found to have the region II insert. The position of the inserts on AA9 structures was determined by mapping these regions on their respective structures as shown in Fig. 2. As there was no crystal structure of a Type 1 PMO with the region I insert in the PDB, homology modeling of the *A. niger* Type 1 PMO was performed to study this region. AA9 proteins are proposed to interact with cellulose through aromatic and polar interactions [37, 38]. Hemsworth et al. [6] indicated that steric congestion of the LPMO active site may also influence the orientation of its interaction with the substrate. As a result, the structural implications of the identified type-specific features were assessed by manually docking AA9 proteins to cellulose. The active site of AA9 proteins possesses planar aromatic residues similar to surface-binding (Type A) CBMs [36]. As such, AA9 structures were aligned onto cellulose Iβ using the planar aromatic residues on the AA9 active site (Fig. 2). The region I insert was found to be associated with planar aromatic residues in both Type 1 and 3 AA9 proteins. This insert was found near the active site residues of both Type 1 and 3 AA9 proteins. More specifically, the region I insert of the modeled Type 1 protein was found to be associated with a planar aromatic residue Trp-34 (Fig. 2). This residue is situated towards the end of this eight-residue insert and is positioned to interact with the cellulose substrate. Similarity, the region I insert found in the Type 3 crystal structure, 3ZUD, is associated with the planar Tyr-24 residue. This Tryptophan residue was highly conserved in most sequences with a region I insert. Planar aromatic active site residues are suggested to be important for the function of these AA9 proteins [17]. Polar residues were also identified in the region I insert of Type 1 sequence, as seen in Fig. 1. Of these, Thr-32 and Lys-33 in the modeled Type 1 structure were orientated to potentially interact with the cellulose substrate. The region I insert in Type 3 sequences was also found to possess polar residues. On the 3ZUD crystal structure, residues Ser-26, Thr-37, Thr-39 and Glu-40 were found to face the substrate suggesting potential involvement in cellulose interaction. The region II insert, specific to Type 2 sequences, was found to lack aromatic residues in the 4EIR crystal structure; however, polar residues were identified within this insert sequence. These included Ser-70, Thr-71, Thr-72, Ser-75 and Asp-78, which are all orientated to interact with cellulose. Unlike the region I, region II insert showed higher sequence variation (Fig. 1). The flat active site of AA9 PMO types is believed to bind the cellulose substrate differently, which is considered to be the cause of the different region selectivity displayed by these enzymes (C1, C4 or both) [6]. The positioning of these type-specific insertions near the cellulose interface further indicates that they may contribute to substrate binding and orientation (Fig. 2).

**Fig. 3:**
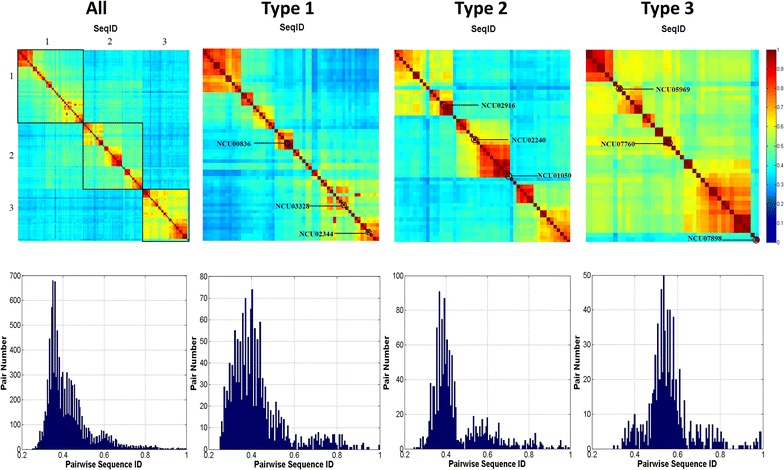
Sequence identity heat maps. The pairwise sequence identity values of all AA9 domains as well as sequences from each type of AA9 PMO are shown. The heat maps at the top show these scores as a color-coded matrix, of every sequence vs every sequence in their respective groupings shown. Heat maps are colored from *blue* to *red* with *red* show high conservation and *blue* showing low conservation. In the first panel (All), the blocks are numbered to indicate sequences from each AA9 PMO type. The positions of the Type 1, 2 and 3 reference sequences within each heat map are indicated. In the histogram at the *bottom*, the pairwise sequence identity values are shown and the number of sequence pairs with this value is shown on the *y*-axis

All vs all sequence identity calculations were performed to observe the similarity between the reference sequences and sequence groups in Fig. 1. Separate alignments were generated for each AA9 PMO type (Additional files 3, 4 and 5 for Type 1, 2 and 3 PMOs, respectively) using PROMALS3D. All vs all sequence identity calculations for the alignments produced (Fig. 3) showed higher sequence conservation in individual AA9 PMO types as opposed to the alignment containing all AA9 domain sequences. The alignment of AA9 domain sequences revealed generally low conservation across all types. Interestingly, three distinct regions of conservation were observed. These conservation regions corresponded to the expected AA9 types showing that even though there was low sequence identity among all AA9 domains, there was higher sequence identity within AA9 PMO types. A mapping of the Type 1, 2 and 3 reference sequences to the heat map (Fig. 3) shows the higher sequence identity shared between these sequences and others from the same type. The heat maps did show some degree of sequence similarity between Type 1 and Type 2 sequences, indicated by the green blocks where these sequences overlap. The histogram for the all vs all sequence identity indicates that the pairwise sequence identities of the majority of AA9 domains fall in the range of 0.3–0.4 indicating the high sequence variation expected from this group of enzymes. Type 1 PMO sequences show high sequence variation; however, distinct conserved boxes of sequences were observed within Type 1 PMOs indicative of possible Type 1 variants.

**Fig. 4:**
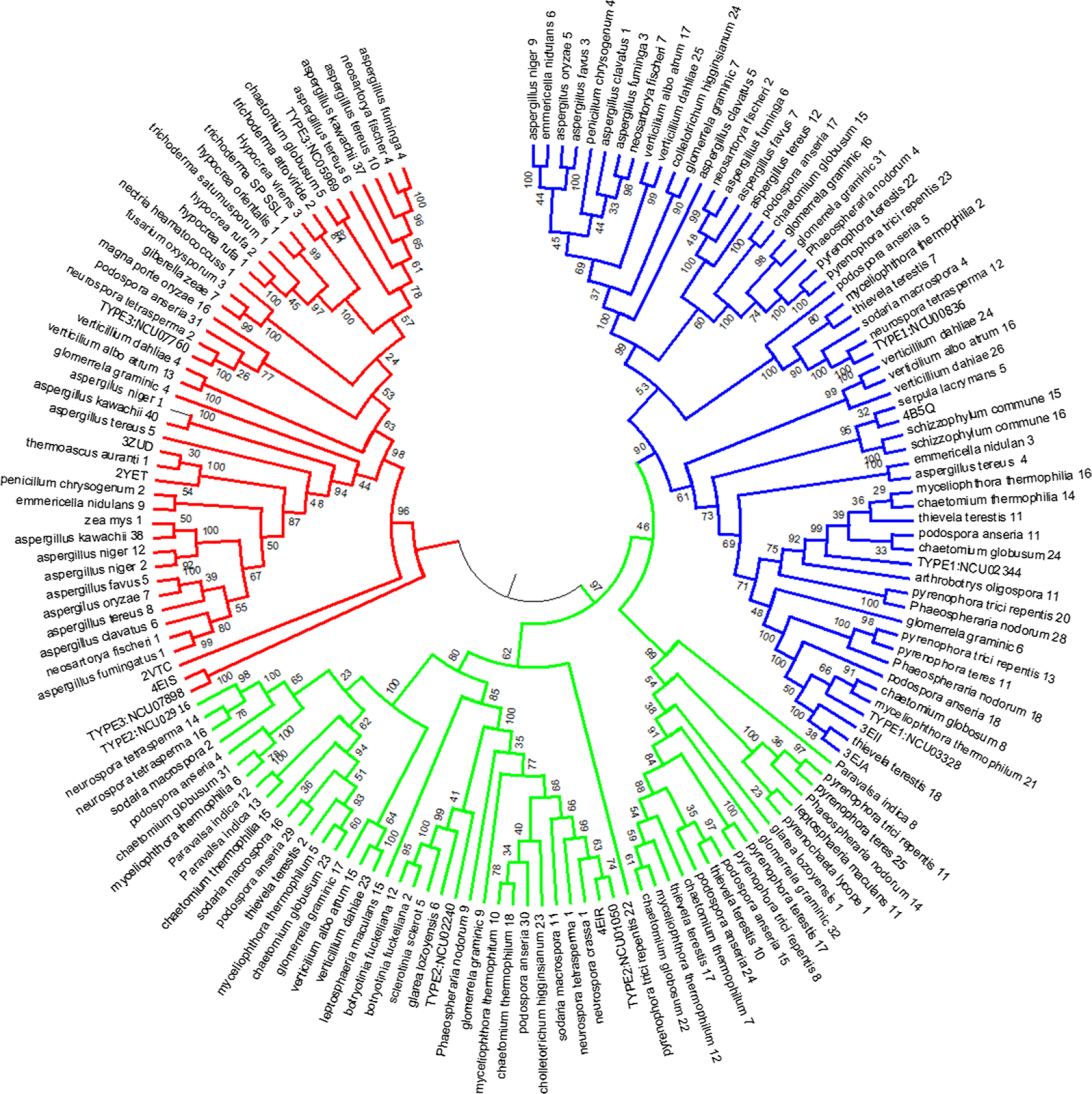
Molecular phylogenetic analysis by maximum likelihood method of AA9 proteins at 90% site coverage. Maximum likelihood tree constructed using MEGA6. Sequences are colored as follows: Type 1—*Blue*; Type 2—*Green*; Type 3—*Red*

**Fig. 5:**
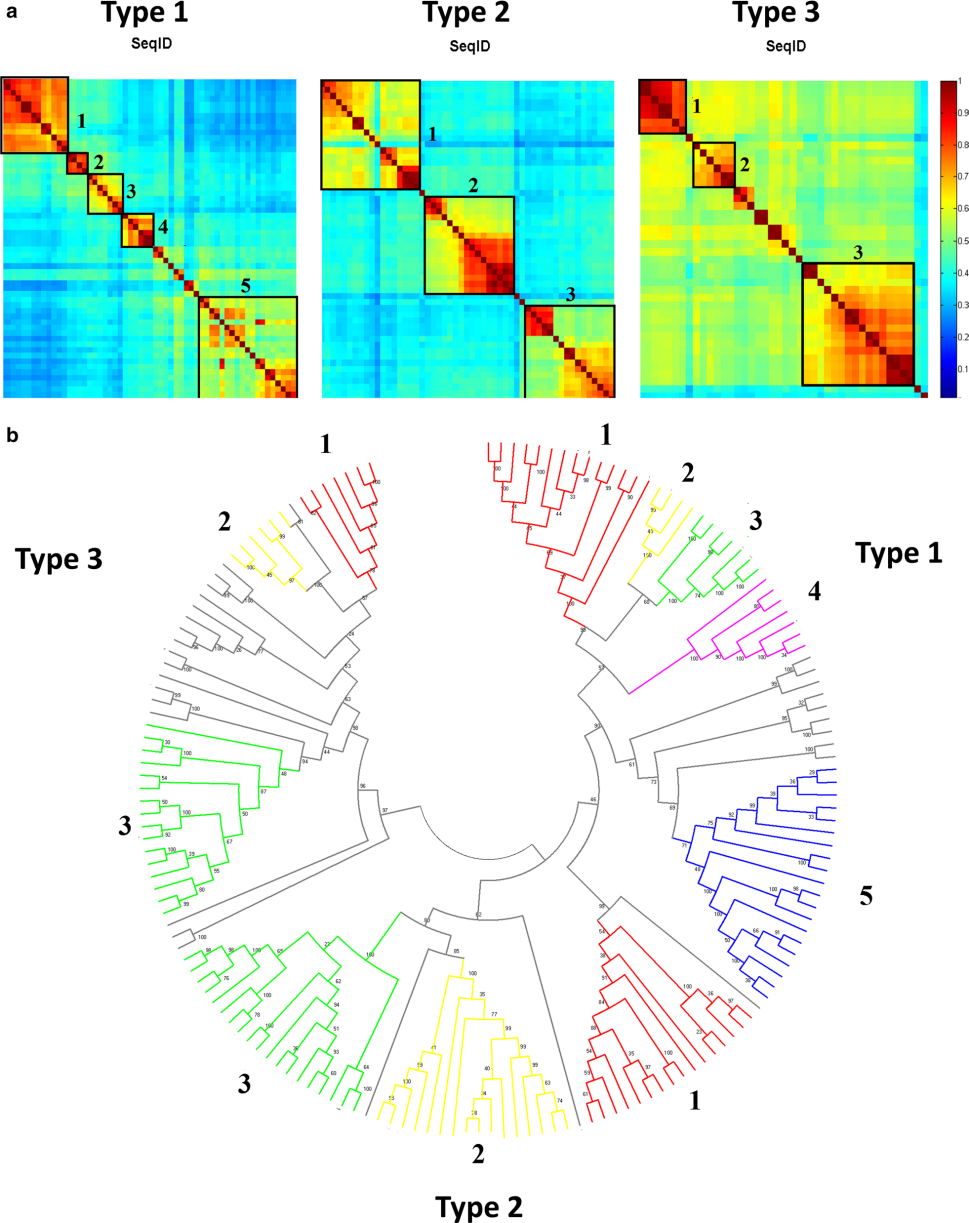
Mapping of sequence identity conservation boxes to the phylogenetic tree. **a** Clusters within the each sequence identity heat map in Fig. 3 are shown as *boxes*. **b** The positions of these sequences are then highlighted on the phylogenetic tree from Fig. 4

The observed Type 1 sequence variation was further investigated on the phylogenetic tree (Fig. 4) revealing the presence of clusters which are found corresponding to the observed conservation boxes in Fig. 3. A mapping of these boxes to the tree is shown in Fig. 5. For Type 2 sequences, three distinct conservation regions were identified. Phylogenetic branches were observed which correspond to the three major Type 2 regions of conservation (Fig. 4). Histograms of sequence identities (Fig. 3) indicated that Type 2 sequences were slightly more conserved than the Type 1 PMOs, with fewer sequence pairs displaying identity values below 0.4. Type 3 sequences showed the highest degree of conservation with most of the sequence identities above 0.5. However, there was little conservation between Type 3 and other types. The presence of conservation regions in AA9 PMO types could be indicative of previously undescribed phylogenetically distinct sub-groupings. Considering the diverse nature of AA9 proteins with respect to the sequence variation as well as modularity of proteins containing an AA9 domain [13], the presence of sub-groups with specialized functions is possible. The histograms show the distribution of sequence identity values across the different groupings. Mean values were also calculated for each. The mean sequence identity value when all sequences were considered was 0.39, with a standard deviation of 0.02. For the Type 1, Type 2 and Type 3 PMOs, the mean sequence identity values measured were 0.44, 0.46 and 0.57, with standard deviations of 0.02, 0.04 and 0.05, respectively. This indicates the higher sequence conservation of the Type 3 sequences when compared to the other AA9 sequences. There were two Type 3 sequences which were found to have very low sequence identity when compared to the rest of the group. These sequences can be clearly seen by lack of conservation in the bottom right corner of the Type 3 heat map. These highly variable sequences were found to be the reference sequence NCU07898, as well as the crystal structure sequence 2VTC. The phylogenetic analysis showed these two sequences to form out-groups with respect to the other Type 3 sequences, which explains the measured lack of conservation.

### Type-specific motifs identified, which also reveal sub-groups

**Fig. 6:**
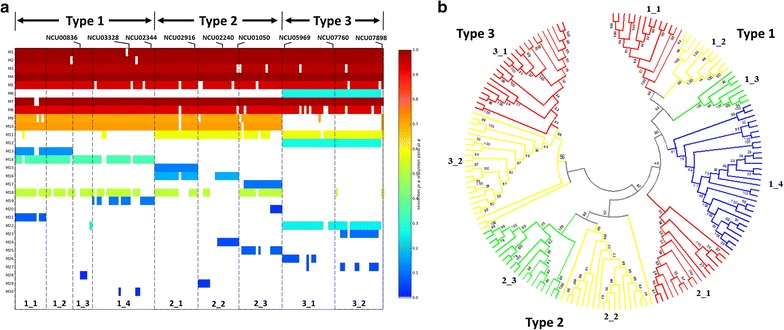
Motif analysis of AA9 domains. **a** Heat map representing the extent of conservation of the identified 30 motifs on AA9 sequences. Sequences are grouped according to type starting from Type 1–3. Motifs are colored based on conservation, as shown in the key. Divisions are indicated to show the sub-groups within each AA9 PMO type identified during motif analysis. The position of each reference sequences from *Neurospora crassa* is also shown on the motif heat maps. **b** Mapping of AA9 sub-groups to the phylogenetic tree produced for AA9 domains. The sub-groups indicated in (**a**) are shown mapped to the phylogenetic tree from Fig. 4

Motif analysis was conducted to investigate the conserved type-specific sequence motifs displayed by AA9 PMO types. A total of 30 motifs were identified from all AA9 domains in the dataset using the MEME webserver. The maximum number of motifs was set at 30, because any number above this resulted in the identification of insignificant motifs. The regular expression for the final motif dataset is shown in Additional file 6. A heat map indicating the conservation of sequence motifs on AA9 domains, as in the order returned by MEME, is displayed in Fig. 6a. The results show that motifs 1, 2, 3, 4, 5, 7 and 8 were common to all of the AA9 PMO types with few sequences lacking these motifs.

Motif analysis of Type 1 sequences revealed the presence of four distinct sequence sub-groups which are characterized by different patterns of conserved motifs (highlighted in Fig. 6a). In the Type 1 sequences, motifs 14 and 18 were found to be common to all four sub-groups. The first Type 1 sub-group (Type 1_1) was characterized by the presence of motifs 13 and 21. The second Type 1 sub-group (Type 1_2) was found to be similar to Type 1_1; however, motif 21 was absent. The Type 1_3 sequences were found to only contain motifs 14 and 18, whereas the final Type 1 sub-group (Type 1_4) contained motif 19 in addition to motifs 14 and 18. In Type 2 sequences, three sub-groups were identified. The Type 2_1 sub-group was found to be associated with motifs 15, 16 and 18. The Type 2_2 sequences were only associated with motif 16. The Type 2_3 sub-group was associated with motifs 17 and 18, with a few sequences also associated with motif 20. Type 3 sequences were found to be least variable sequences with respect to motifs with all sequences possessing motif 22. Due to the presence of Type 3 variable motifs, the sequences were grouped into two sub-groups. The first Type 3 sub-group (Type 3_1) was found to be associated with 26 while the second sub-group (Type 3_2) was associated with motif 23 and 27.

Prior to motif analysis, the input sequences used were reordered based on the observed phylogenetic tree clustering order (Fig. 4). This was performed to identify phylogenetic clusters corresponding to any motif sequence sub-groups which may be present. These sub-groups were mapped to the phylogenetic tree shown, and were found to be phylogenetically distinct (Fig. 6b). Sequences in these sub-groups each branched off from a common point on the tree, with the exception of the outgroup sequences in sub-groups 2_2 and 3_2. There was an interesting overlap between these motif sub-groups and the sequence subsets identified within conserved boxes on the sequence identity heat maps (Fig. 5b). These subsets did not describe all sequences on the phylogenetic tree, but they did fall within the sub-groups identified through motif analysis (Fig. 6b). Another interesting observation was that motif 21, found in the Type 1_1 sequences, was found to be the 8-residue insert occurring in region I of the AA9 domain alignment (Fig. 1) and shown in Fig. 2 (Model AA9).

Motifs 9, 10 and to some extent 18, were common to both Type 1 and 2 sequences, but almost entirely absent in Type 3 sequences. Motif 18 was found in the signaling peptide regions of AA9 domains. Motif 14 was the only Type 1-specific motif found to be present in most Type 1 sequences. There were no notable motifs conserved exclusively across the majority of Type 2 sequences. Motif 11 was commonly found in Type 2 sequences, as well as Type 3 sequences. Three sequence sub-groups were identified for Type 2 sequences.

**Fig. 7:**
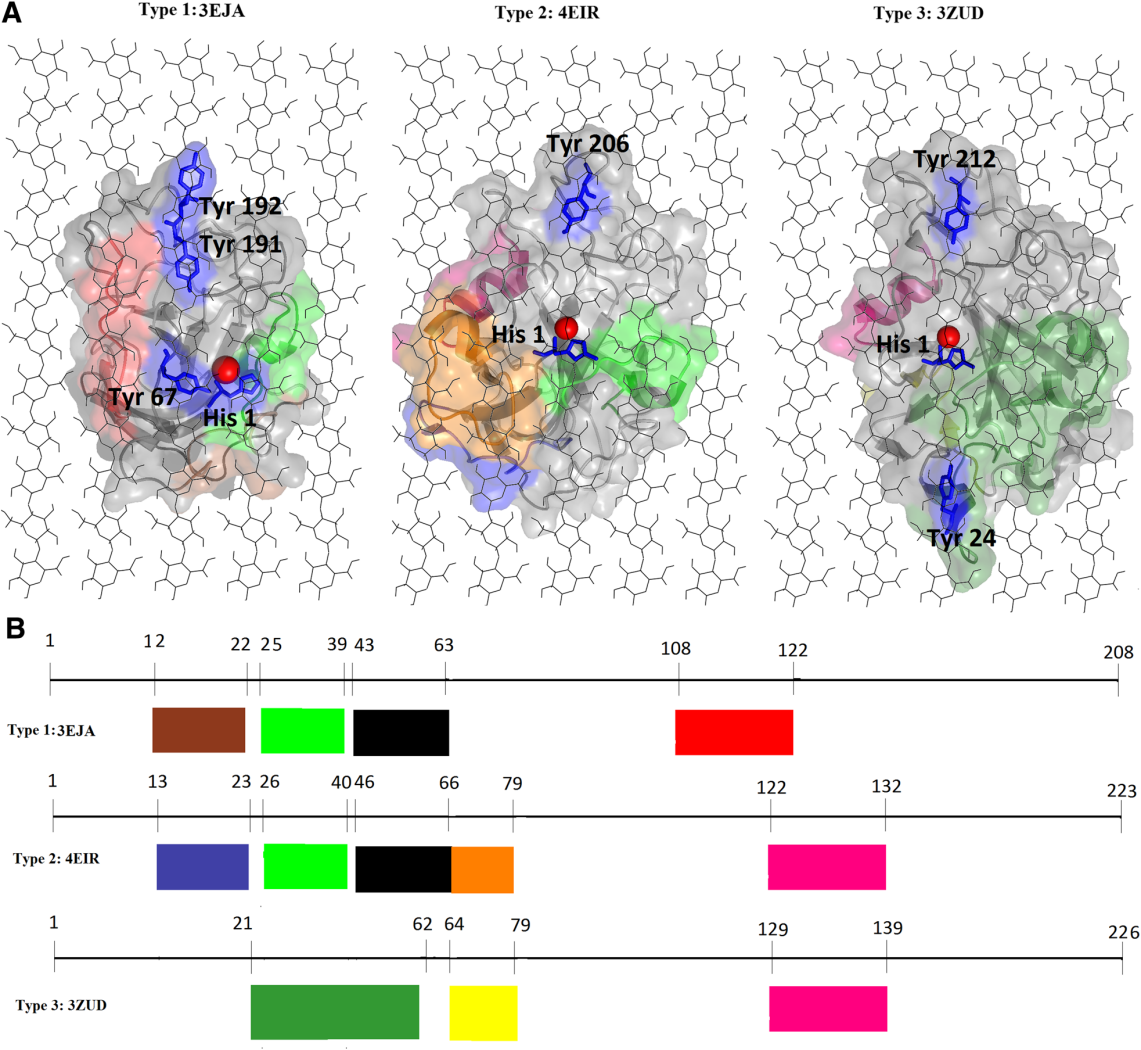
Visualization of type-specific motifs on crystal structures and linear sequences. **a** Structural visualization of type-specific motifs. Crystal structures 3EJA, 4EIR and 3ZUD were used to represent the AA9 PMO types, respectively. The AA9 structures were aligned onto cellulose Iβ using the planar aromatic residues on the AA9 active site. The flat surface aromatic residues are represented as sticks and are colored in *blue*, with the type II copper ion shown as a *red sphere*. **b** Linear representation of N-terminal type-specific motifs. The motifs are visualized on structures 3EJA, 4EIR and 3ZUD linear sequences to show type-specific motifs. The motifs shown are motifs *6*, *9*, *10*, *11*, *12*, *14*, *15*, *16* and *19*, colored based on where they are mapped to the structure (**a**). The motifs are colored as follows: motif 6 *dark green*, motif 9 *green*, motif 10 *black*, motif 11 *pink*, motif 12 *yellow*, motif 14 *brown*, motif 15 *orange*, motif 16 *dark blue* and motif 19 *red*

The high level of sequence conservation displayed in Type 3 sequences was reflected at a motif level, with motifs 6, 12 and 22 being conserved in most of the sequences. Motifs 6 and 12 were specific to Type 3 sequences, lacking only in the three proteins (crystal structures 4EIS, 2VTC and the reference sequence NCU07898) that formed the out-group in the phylogenetic analysis. This observation was based on variability of motifs observed in this region. Motif analysis allowed for the identification of type-specific motifs. These motifs were determined to be motif 6, 9, 10, 11, 12, 13, 14, 15, 16, 17, 18 and 19. Type-specific motifs were mapped to crystal structures 3EJA, 4EIR and 3ZUD, representing Type 1, 2 and 3 PMOs, respectively (Fig. 7a). Motifs 13, 17 and 18 were not included in Fig. 7 as these motifs are not present in the representative crystal structures used. Motifs 13 and 17 were sub-group specific, whereas motif 18 was found to occur in the signal peptide region, which is cleaved from mature protein. Results of this analysis showed that areas which play roles in type specificity appear to be located on the active site surface. As proposed by Hemsworth et al. [6], the active site configuration of different AA9 PMO types may affect substrate binding which in turn affects region-selectivity. Our findings are consistent with this proposal as the active sites of different AA9 PMO types varied in motif compositions. Common motifs among AA9 PMO types were also observed in Fig. 7. For example, motif 9 which was found to correspond to a loop associated with a small helical region, which is common to both Type 1 and Type2 PMOs. A similar observation was made between Type 2 and Type 3 PMOs, where motif 11 was found associated with both. As with multiple sequence alignment results, motif analysis was able to detect variability in the N terminus of AA9 domain sequences (Fig. 7b). For both Type 1 and 2 AA9 proteins, motif 9 was positioned to potentially interact with cellulose chains 4 and 5 (Fig. 7). In Type 3 AA9 proteins, this region is replaced by the larger motif 6. Similarly, Type 1 AA9 proteins were found to be associated with motif 19 which is positioned for possible interaction with cellulose chain 2. In Type 2 AA9 sequences, motif 19 is replaced by motif 11 and 15 and motif 11 only in the Type 3 structure (Fig. 7). The manual docking results indicate that the type-specific motifs identified generally occur in regions that are in proximity with cellulose chains. The AA9 sub-groups identified are based on the different motif compositions of different sequences within each AA9 type (Fig. 6). The presence of these different motifs is likely to affect the region selectivity of AA9 proteins due to the different binding configurations observed among AA9 types; however, molecular dynamics studies are required to further the understanding of these findings.

### Physicochemical differences is observed between different AA9 PMO types

**Fig. 8:**
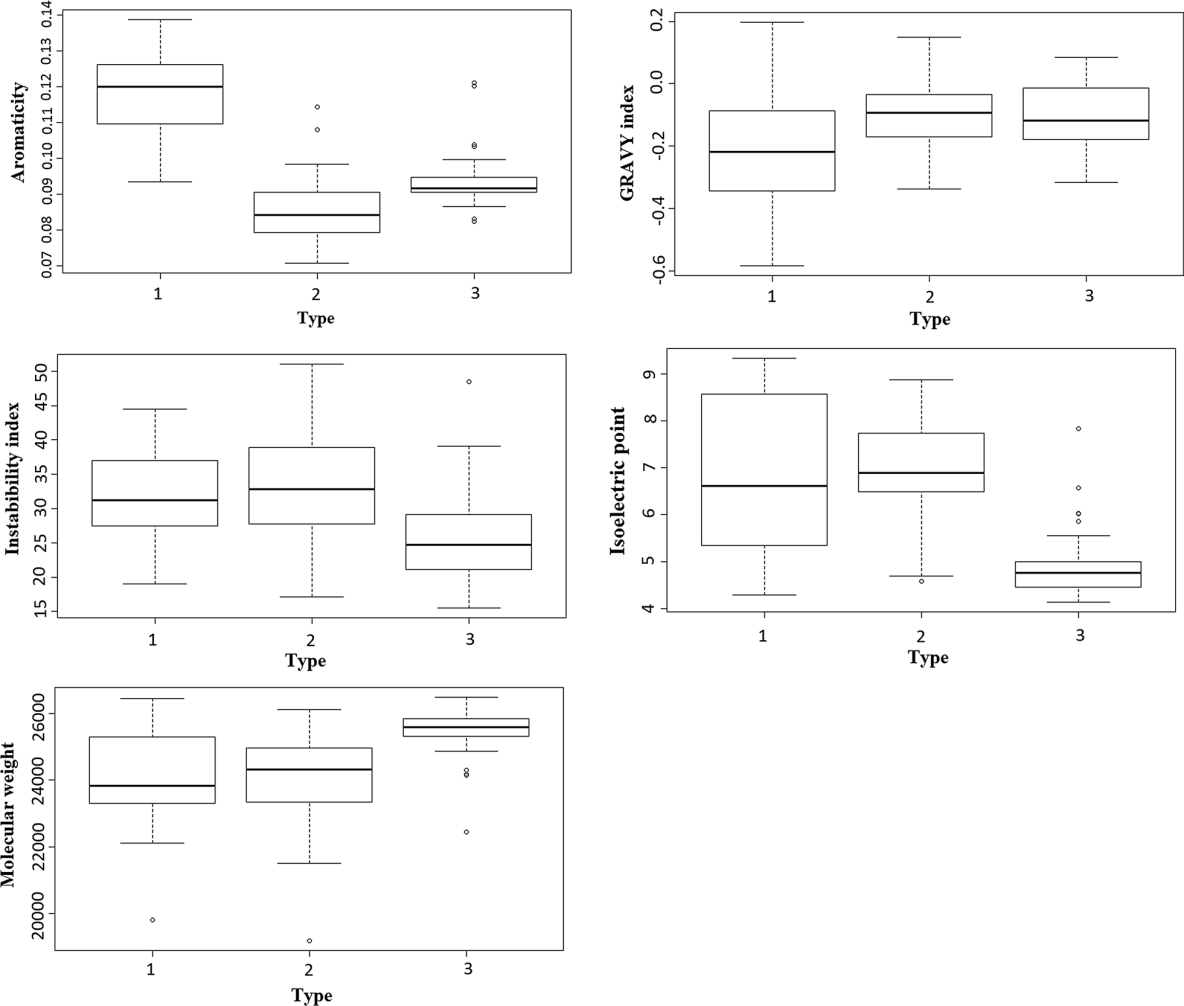
*Boxplot* representation of the distribution of the different physicochemical properties analyzed for Type 1, 2 and 3 in AA9 protein sequences. In their respective order, the properties analyzed were: Aromaticity, GRAVY index, Instability index, Isoelectric point and Molecular weight. The numbering corresponds the respective types. The *dark central line* shows the median of the data. The* lines* on either side of the median show the* upper* and* lower* quartile which represent data that fall above 75% and data that falls below 25%, respectively. The *lines* at the extremes represent the *lower* and *upper fence* which allows for the detection of outliers. 1.5 IQR (interquartile range) of the* upper* or* lower* quartile results in the fences. Outliers are indicated as *dots* that occur outside the whiskers of each plot

The physicochemical properties of AA9 domains were investigated to identify features which are unique to certain AA9 PMO types (Fig. 8). For each physicochemical property, three boxplots were generated to represent Type 1, 2 and 3 AA9 proteins. The first feature analyzed was the aromaticity of AA9 domains. Aromaticity is a relative measure of the proportion of aromatic residues in a particular protein. Due to the postulate that the aromatic residues are critical to the function of these enzymes [13], we evaluated the aromaticity of AA9 domains to see if any type-specific differences could be observed. Figure 8 suggests that the distributions of aromaticity in AA9 domains are type specific. Type 1 PMOs were found to have the highest aromaticity while Type 2 PMOs displayed the lowest. The active site surface of AA9 domains is prominent with surface-exposed aromatic residues. Aromatic residues have long been believed to be crucial for interacting with cellulose in various cellulose-interacting enzymes [13]. As such, differences in the observed distribution of aromaticity among the various PMO types could prove to be important. The box plots revealed a great deal of variability of the calculated physicochemical properties, even within each type. To detect significant differences between the datasets, *t* tests were performed using R. The results of these are shown in Table 2. When comparing aromaticity values, the differences between Type 1 sequences and both Type 2 and Type 3 sequences were found to be significant with a *p* value well below 0.05.

**Table 2:** Results of the *t* test, performed to compare the means of the different physicochemical properties at a 5% level of significance

	Type 1	Type 2
Aromaticity		
Type 2	2.20E−16	
Type 3	2.72E−12	1
Instability index		
Type 2	0.6848	
Type 3	5.47E−06	1.92E−05
Molecular weight		
Type 2	0.5214	
Type 3	1	1
GRAVY index		
Type 2	1	
Type 3	1	0.55
Isoelectric point		
Type 2	0.5705	
Type 3	3.54E−11	2.52E−16

In each block, a *p* value is given, with a value < 0.05 indicating that the means of the two groups are significantly different

A number of outliers were identified in the aromaticity datasets. Type 2 sequences were found to have two outliers which were *Verticillium albo*-*atrum* AA9 homolog 15 and *Leptosphaeria maculans* homolog 15. The phylogenetic clustering observed in Fig. 4 places these sequences in two separate sister groups in the Type 2 phylogenetic branch. Both these sequences were found to occur on the outer group branches of their respective sister groups suggesting that these sequences are more diverse than the other sequences. For Type 3 sequences, aromaticity outliers above the upper quartile were identified as *Verticillium albo*-*atrum* homolog 13, *Magnaporthe oryzae* homolog 16, NCU07898 and the crystal structure 2VTC, whereas the sequences *Aspergillus fumigatus* homolog 4 and *Penicillium chrysogenum* homolog 2 were found below the lower quartile. Phylogenetic clustering of the Type 3 aromaticity outliers shows the NCU07898 and 2VTC sequences form outer groups early on the Type 3 phylogenetic branch while the other outliers where found to be distributed in the phylogenetic branch. These two sequences were also found to have low sequence identity relative to the other Type 3 sequences (bottom right corner of the Type 3 heat map, Fig. 3).

The GRAVY index for all AA9 PMO types was evaluated to assess the extent to which each of the AA9 PMO type is able to interact with water. All three AA9 PMO types were found to have negative GRAVY indices which indicate that these sequences are hydrophilic. However, all sequences in the upper quartile of the box plots for each type were found to be associated with hydrophobic GRAVY indices. No significant differences between the proportions of GRAVY indices amongst AA9 PMO types were identified (Table 2).

It has been determined that proteins with an instability index below 40 can be regarded as being stable [27]. A majority of AA9 sequences was determined to have instability index values below 40 which indicate that AA9 domains are stable, with Type 3 PMOs having the lowest instability index. Notably, there were few sequences in all three types which were found to have instability values above 40. A single Type 3 outlier was identified for the instability index. The sequence was determined to be *Fusarium oxysporum* homolog 3 with an instability index of 48.477 meaning this sequence is predicted as being highly unstable. No phylogenetic influence was observed for this outlier.

The isoelectric points (pI) of the three AA9 PMO types were calculated to assess the functional pH of AA9 domains. Type 1 and 2 PMOs displayed a wide pH range with sequences found to have acidic, neutral and basic pI values. Type 3 PMOs were only found to have acidic pI values, which span between neutral and highly acidic range, with the exception of a single protein. For isoelectric points, Type 3 sequences were found to have four outliers which were sequences *Magnaporthe oryzae* homolog 16, *Aspergillus clavatus* homolog 6, NCU07898 and the crystal structure 2VTC. This analysis identified a broad pH range that Type 1 and 2 PMOs may be functional at, suggesting that different AA9 proteins belonging to these types may be required at different environmental conditions. The acidic pI measurements for Type 3 sequences could suggest a more specialized role for these enzymes. In terms of molecular weight, Type 1 and 2 PMOs had similar sizes, with both being between 19 and 26.5 kDa. Type 3 PMO sequences were found to be significantly larger than Type 1 and 2 PMOs (Table 2) with a size range of approximately 23–27.6 kDa. Outliers were identified for all three AA9 PMO types. Type 1 and 2 each had a single outlier while Type 3 sequences had five outliers. The outliers were *Arthrobotrys oligospora* homolog 11 and *Leptosphaeria maculans* homolog 15 for Type 1 and 2, respectively, and for Type 3 the outliers were *Chaetomium globosum* homolog 5, *Aspergillus terreus* homolog 5, NCU07898, 2VTC and 3ZUD. With the exception of NCU07898 and 2VTC, these outliers were not observed within the results from phylogenetic clustering.

**Fig. 9:**
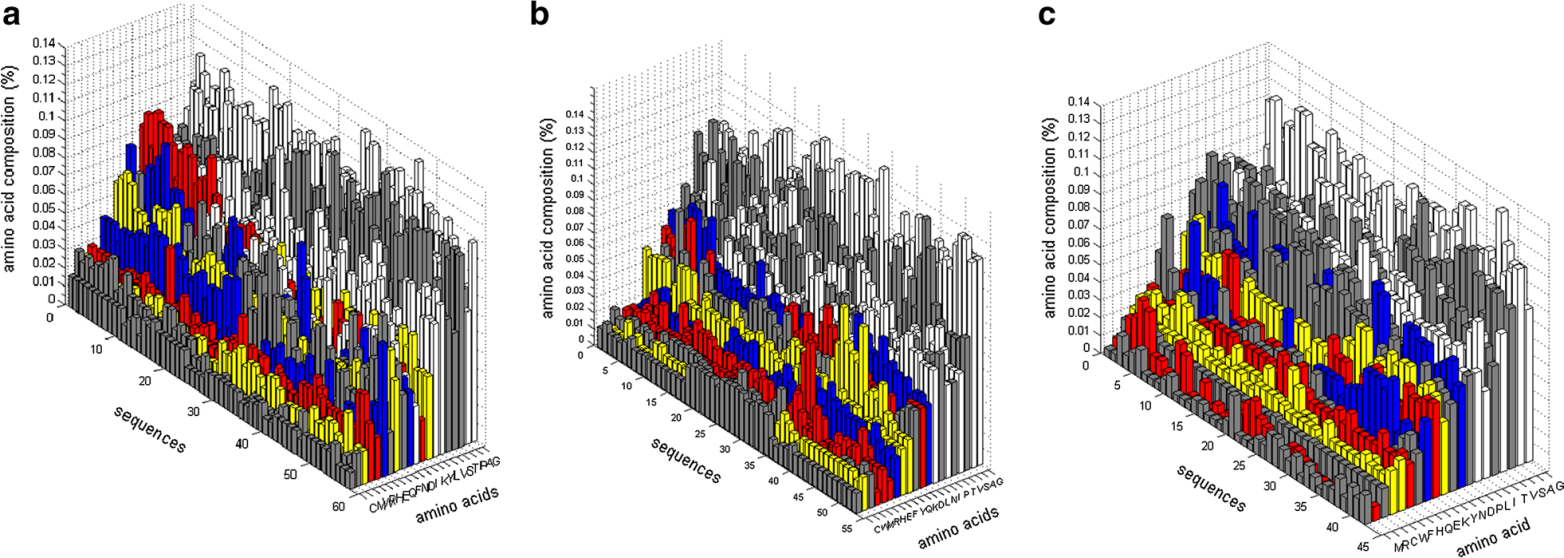
3D plots showing the amino acid composition of the three AA9 PMO types. Amino acids counts are shown as a percentage for each sequence. The occurrence frequency of each amino acid is ranked from lowest to highest for clarity. Residues are colored as follows: Aromatic—*Yellow*; Positively charged—*Red*; Negatively charged—*Blue*; Hydrophobic—*White*. **a** Type 1, **b** Type 2 and **c** Type 3 PMO sequences

The amino acid composition for AA9 domains was evaluated for all three AA9 PMO types (Additional file 7). The findings are summarized in the 3D plots in Fig. 9 which show the amino acids ranked from the lowest to the highest average occurrence show the predominance of residues in each type. Individual sequence analysis of amino acid frequencies revealed a high variability in amino acids conservation for all three types. Across all AA9 PMO types, hydrophobic residues (Alanine, Glycine, Isoleucine, Valine and Leucine) were found to occur at higher frequencies than other residues. Some polar residues (Serine, Threonine and Asparagine) occurred at relatively high frequency, while Glutamic acid, Cysteine and Methionine were less prevalent in AA9 sequences. Across all AA9 domains, both charged residues and aromatic residues were found to occur at the lowest frequencies. The low number of aromatic residues was also seen in the aromaticity calculations (Fig. 8). Though these residues are considered to be important to the interaction between AA9 enzymes and their substrates, they have been found to be evenly distributed throughout the AA9 domain and not just within the active site [13]. Variation in amino acid composition was observed among all AA9 sequences; however, no type-specific variation could be observed.

## Conclusions

The aim of the study was to evaluate sequence and structural features that can be used to further elucidate the highly variable AA9 PMO types. The initial approach was to utilize all AA9 sequences in the Pfam database, this proved difficult due to the presence of short fragments and highly divergent sequences, which were subsequently removed, decreasing the size of the dataset from 827 sequences to 153 sequences.

Identifying type-specific sequence and structural features was made possible through the use of reference sequences from *Neurospora crassa*. The inclusion of the reference sequences in all the analyses carried out created a reference to allow association of certain features with a specific AA9 PMO type. Sequence analysis was carried out to investigate any unique features that respective AA9 PMO types may have. The obtained sequences were aligned, phylogenetically classified, motifs were analyzed and the physicochemical properties were determined. The identification of inserts which play a role in type specificity was achieved through multiple sequence alignment of AA9 domain sequences. It was observed that the absence or presence of either insert in region I or II was important in determining type specificity, as shown in previous studies [19]. However, due to the highly variable nature of these AA9 domain sequences the inserts alone are not enough to draw conclusions about type specificity. The inserts were found in the N terminus suggesting that this region is crucial for elucidating type specificity. Structural analysis was carried out by manually docking the AA9 catalytic domains to crystalline cellulose. This revealed that the insert in region I may interact with cellulose through a conserved planar Tryptophan residue. The influence of the region II insert on Type 2 specificity was not as clear, but is likely influenced by polar residues in this region. In addition, phylogenetic analysis at 90% gap deletion was carried out to reveal AA9 PMO types. Overall, AA9 sequences were shown to be a very diverse group of enzymes. However, the analysis of sequences at type level shows that the AA9 domains are more conserved within their types. It was revealed that these domains have specific motifs that distinguish between different AA9 PMO types and that the majority of these motifs occur in the N-terminal of the AA9 domain. Heat maps were sensitive enough to identify groupings of sequences within the different AA9 PMO types that were reflected in the phylogenetic tree. These proposed sub-groups were further characterized by identifying extra or slightly different motif organizations occurring among AA9 PMO types. When mapped to the phylogenetic tree, many of these sub-groups were found to be phylogenetically distinct. The motif analysis yielded similar results with sequence alignment which supports the argument that the N terminus of AA9 domains is important for type specificity. The potential implications of these sub-groups will need to be elucidated through further study of the AA9–cellulose interface and the involvement of the motifs identified. Initial analysis of this interface indicates that some of these motifs may be involved in the interaction with cellulose.

The physicochemical properties of AA9 PMO types were evaluated. The analysis of all AA9 domains indicated that these are a stable group of enzymes. It was determined that Type 3 sequences are generally acidic in nature while Type 1 and 2 PMO sequence do not appear to have any certain preference with respect to pI. This finding could suggest that fungal organisms have a repertoire of enzymes that may be used in different environmental conditions. Aromatic residues have always been known to be important features in AA9 LPMOs from the metal-coordinating residues to the planar aromatic residues found in the active site [17]. However, analysis of the global structural distribution of aromatic residues on AA9 domain structures, as well as the effect of distribution on type specificity is an aspect that was previously not understood. It was found that aromatic residues on AA9 domain are distributed throughout this structure; however, aromatic residues tend to protrude out into the active site surface, while those located away from active site surface tend to be buried. This observation offered no distinction between AA9 PMO types; however, it did implicate these aromatic residues in substrate interaction [13]. The relative aromaticity across AA9 domains was evaluated. The findings suggest that different AA9 PMO types tend to favor different compositions of aromatic residues which could result in different enzymatic functions, with Type 1 PMOs being most prominent in measured aromaticity.

This study was successful in identifying wide-selection AA9 sequences from each type. The analysis of individual AA9 PMO types was able to reveal the diverse nature of observable features of AA9 domains. As diverse as these domains are, there were type-specific features observed. The main aim of the study was to identify sequence and structural features that are specific to AA9 PMO types. During the course of the research, the aims were met and we were successful in determining a means of distinguishing between the various AA9 PMO types.

Structural features were identified on these enzymes which warrant the need for further investigation through the use of techniques such as molecular dynamics as well as docking. This would shed further insight into interactions with cellulose substrate, displayed by different types of AA9 PMOs, as well as the sub-groups found within each type.

## Additional files

**Additional file 1.** Names and accession numbers of all AA9 sequences used in this study. Names indicated are specific to this study. Most of the sequences retrieved had no assigned name in Uniprot; as such, the sequences were named according to the host organisms with a number shown at the end to account for organisms that express several different AA9 LPMOs.

**Additional file 2.** Multiple sequence alignment of all AA9 domain sequences used in this study.

**Additional file 3.** Multiple sequence alignment of the Type 1 PMO sequences used in this study.

**Additional file 4.** Multiple sequence alignment of the Type 2 PMO sequences used in this study.

**Additional file 5.** Multiple sequence alignment of the Type 3 PMO sequences used in this study.

**Additional file 6.** Motifs identified on AA9 domain sequences. The regular expression describes the sequence of the motif, with residues in square bracket showing various amino acids that can occur in that position. The “width” column indicates the length of the motif and the “sites” column displays the number of times the motif occurs in the input dataset.

**Additional file 7.** Amino acid composition values for all AA9 domains evaluated.
