# Supplementary material for: Bioinformatic characterization of type-specific sequence and structural features in auxiliary activity family 9 proteins
Source: Biotechnol Biofuels. 2016 Nov 9;9:239. doi: 10.1186/s13068-016-0655-2 (PMC5101804; doi:10.1186/s13068-016-0655-2)
Supplement: Supplementary file 1 — Additional file 1. Names and accession numbers of all AA9 sequences used in this study. Names indicated are specific to this study. Most of the sequences retrieved had no assigned name in Uniprot; as such, the sequences were named according to the host organisms with a number shown at the end to account for organisms that express several different AA9 LPMOs. [file 13068_2016_655_MOESM1_ESM.pdf]

Type 1 sequence name	Uniprot acession
aspergillus_fumingatus_3	B0XU33.1
cholleotitracum_higginsianum_15	H1W1L4
glomerrela_graminic_7	E3Q410.1
aspergillus_clavatus_5	A1CC32.1
aspergilus_oryzae_5	Q2UI80.1
aspergillus_favus_3	B8N7Z7.1
aspergillus_niger_9	G3XUH5.1
emmericella_nidulans_6	Q5B8T4.1
aspergillus_clavatus_1	A1C9D5.1
neosartorya_fischeri_7	A1DI09.1
penicillum_chrysogenum_4	B6HG02.1
verticilium_albo_atrum_17	C9SUD7.1
verticillium_dahiae_25	G2XHH3.1
pyrenophora_teres_22	E3S7R0.1
Phaeospheraria_nodorum_4	Q0UB83.1
pyrenophora_trici_repentis_23	B2WHT5.1
podospora_anseria_17	B2ASU3.1
glomerrela_graminic_16	E3QS38.1
glomerrela_graminic_31	E3QZY5.1
chaetomium_globosum_15	Q2H3R3.1
neosartorya_fischeri_2	A1DN02.1
aspergillus_tereus_12	Q0CMK3.1
aspergillus_favus_7	B8NYH4.1
aspergillus_fumingatus_6	B0Y7Z8.1
serpula_lacrymans_5	F8PCC0.1
schizzophylum_commune_15	D8QHF7.1
schizzophylum_commune_16	D8QHH2.1
chaetomium_globosum_8	Q2GWI6.1
pyrenophora_trici_repentis_13	B2W3J4.1
Phaeospheraria_nodorum_18	Q0U089.1
podospora_anseria_18	B2ASV8.1
thievela_terestis_18	G2RGE5.1
myceliophthora_thermophilum_21	G2QNY4.1
emmericella_nidulans_3	Q5AZ52.1
aspergillus_tereus_4	Q0CJQ7.1
podospora_anseria_11	B2APE9.1
glomerrela_graminic_6	E3Q3S7.1
arthrobotrys_oligospora_11	G1X8Q3.1
pyrenophora_trici_repentis_20	B2WB22.1
Phaeospheraria_nodorum_28	Q0V599.1
myceliophthora_thermophilum_16	G2QJR7.1
thievela_terestis_11	G2R6N0.1
chaetomium_globosum_24	Q2GW98.1
chaetomium_thermophilum_14	G0SB71.1
pyrenophora_teres_11	E3RTH1.1
myceliophthora_thermophilia_2	G2Q4M0.1

sodaria_macrospora_4	F7VRX4.1
neurospora_tetrasperma_12	F8N2R6.1
thievela_terestis_7	G2QZK6.1
podospora_anseria_5	B2ADY5.1
verticillium_dahiae_24	G2XGG6.1
verticilium_albo_atrum_16	C9SU69.1
verticillium_dahiae_26	G2XGG6.1

Type 2 sequence names	Uniprot accession
glomerrela_graminic_9	E3Q9F3.1
glarea_lozoyensis_6	H0EI65.1
botryotinia_fuckeliana_2	A6RKN5.1
leptosphaeria_maculans_15	E5A955.1
pyrenophora_trici_repentis_22	B2WFW7.1
botryotinia_fuckeliana_12	G2Y9L4.1
sclerotinia_sclerot_5	A7EV93.1
Phaeospheraria_nodorum_9	Q0V783.1
chaetomium_thermophilia_18	G0SH69.1
colletotrichum_higginsianum_23_123	H1W1E5.1
podospora_anseria_30	B2B5J7.1
myceliophthora_thermophilia_10	G2QA92.1
neurospora_crassa_1	G2Q9F7.1
neurospora_tetrasperma_1	F8MB10.1
sodaria_macrospora_11	F7VXA8.1
Paravalsa_indica_8	G4TGI4.1
pyrenophora_trici_repentis_11	B2W2K6.1
leptosphaeria_maculans_11	E5A089.1
Phaeospheraria_nodorum_14	Q0V4B8.1
pyrenochaeta_lyocope_1	G9JLA8.1
pyrenophora_terestis_25	E3S9U5.1
glarea_lozoyensis_1	H0ECR5.1
myceliophthora_thermophilia_12	G2QCJ3.1
chaetomium_thermophilum_7	G0S408.1
podospora_anseria_15	B2AS19.1
thievela_terestis_10	G2R1U0.1
podospora_anseria_24	B2AVF1.1
pyrenophora_teres_17	E3RY70.1
pyrenophora_trici_repentis__8	B2VZV1.1
chaetomium_globosum_22	Q2H8N9.1
thievela_terestis_17	G2RB73.1
glomerrela_graminic_32	E3R0D2.1
verticillium_dahiae_23	G2XD59.1
verticilium_albo_atrum_15	C9SU05.1
glomerrela_graminic_17	E3QST8.1
chaetomium_globosum_23	Q2HGH1.1
podospora_anseria_29	B2B4L5.1
chaetomium_thermophilum_15	G0SDM5.1

sodaria_macrospora_16	F7W4L9.1
myceliophthora_thermophilum_5	G2Q7A5.1
thievela_terestis_2	G2QQL2.1
Paravalsa_indica_13	G4TLJ0.1
Paravalsa_indica_12	G4TLI9.1
myceliophthora_thermophilia_6	G2Q9F7.1
chaetomium_globosum_31	Q2GW98.1
podospora_anseria_4	B2ADG1.1
sodaria_macrospora_2	F7VLD7.1
neurospora_tetrasperma_16	G4U541.1
neurospora_tetrasperma_14	F8N2R6.1

Type 3 sequence names	uniprot accession
giberella_zeae_7	I1RPX8.1
fusarium_oxysporum_3	F9FC43.1
nectria_heamatococcuss_1	C7YUC8.1
verticilium_albo_atrum_13	C9SWG7.1
verticillium_dahiae_4	G2WVF8.1
neurospora_tetrasperma__2	F8MEC6.1
magna_porte_oryzae_16	G4N560.1
podospora_anseria_31	B2B686.1
hypocrea_orientalis_1	H9C5T5.1
trichoderma_SP_SSL_1	B5TYI4.1
Hypocrea_virens_3	G9N0U1.1
trichoderma_atroviride_2	G9NS04.1
hypocrea_rufa_1	D9IXC6.1
hypocrea_rufa_2	H2KXF9.1
trichoderma_saturnusporum_1	D3JTC4.1
aspergillus_kawachii_37	G7XJV9.1
aspergillus_tereus_6	Q0CXX8.1
neosartorya_fischeri_4	A1DKL1.1
aspergillus_fumingatus_4	B0XZE1.1
aspergillus_tereus_10	Q0CQ24.1
chaetomium_globosum_5	Q2GSI4.1
aspergillus_fumingatus_1	B0XR28.1
neosartorya_fischeri_1	A1D2G7.1
aspergillus_niger_1	G3XUH5.1
aspergillus_kawachii_40	G7XVK2.1
aspergillus_tereus_5	Q0CLL8.1
emmericella_nidulan_9	Q5BEI9.1
penicillum_chrysogenum_2	B6H3A3.1
aspergillus_niger_2	A2QR94.1
aspergillus_niger_12	G3YHB3.1
aspergillus_kawachii_38	G7XNT9.1
zea_mys_1	B4FA31.1
aspergillus_clavatus_6	A1CPL3.1
aspergillus_tereus_8	Q0D0T6.1

aspergilus_oryzae_7	Q2UNV1.1
aspergillus_favus_5	B8NID4.1
glomerrela_graminic_4	B5WYD8.1
