# Supplementary material for: Bioinformatic characterization of type-specific sequence and structural features in auxiliary activity family 9 proteins
Source: Biotechnol Biofuels. 2016 Nov 9;9:239. doi: 10.1186/s13068-016-0655-2 (PMC5101804; doi:10.1186/s13068-016-0655-2)
Supplement: Supplementary file 2 — Additional file 2. Multiple sequence alignment of all AA9 domain sequences used in this study. [file 13068_2016_655_MOESM2_ESM.pdf]

```
                                           10        20        30        40        50        60
                                  ....|....|....|....|....|....|....|....|....|....|....|....|
serpula_lacrymans_5/1-229       1 M---------KSITLLSIAA-VLLPSVSAHYRWTSL----VVGS-TITTA-YEYVR---- 40
schizzophylum_commune_15/1-228  1 M---------RVPTL-LAAA-PLATTALAHYTLPVL----VVNG-EPSGE-WVNIR---- 39
schizzophylum_commune_16/1-228  1 M---------RLSSL-IATA-SLAASALAHYTLPTL----IVNG-QPSGE-WVNIR---- 39
chaetomium_globosum_8/1-223     1 M---------LTTT--FALL-GAALGASAHYTLPKV----GSGS-DW-----QHVRRA-- 36
pyrenophora_trici_repentis_13/  1 M---------KTSA--LLIA-AGATLASAHYTLPSI----NGDG-TW-----VHVRQA-- 36
pyrenophora_teres_11/1-209      1 M---------KTSA--LILA-AGATLASAHYTLPSI----NSDG-TW-----VHVRQA-- 36
Phaeospheraria_nodorum_18/1-21  1 M---------KTST--VLLA--AASVASAHYTLPIV----NGDS-AW-----THVRQA-- 35
podospora_anseria_18/1-223      1 M---------LASL--ALVL-STALSATAHYTLPRV----GNGA-DW-----QHVRRA-- 36
thievela_terestis_18/1-224      1 --------MLANGA--IVFL-AAALGVSGHYTWPRV----NDGA-DW-----QQVRKA-- 37
myceliophthora_thermophilia_21  1 M---------LTTT--FALL-TAALGVSAHYTLPRV----GTGS-DW-----QHVRRA-- 36
TYPE1:NCU03328/1-229            1 M---------LPSI--SLLL-AAALGTSAHYTFPKVWANSGTTA-DW-----QYVRRA-- 40
emmericella_nidulans__3/1-229   1 M---------KSGLL-FTTA-SLALTASAHYVFPAL----VQDG-AATGD-WKYVRDW-- 41
aspergillus_tereus_4/1-228      1 M---------KGSYL-LGSA-MLAATSYAHYVFPAL----IKDG-EATPD-WKYVRQW-- 41
podospora_anseria_11/1-231      1 M---------KASTT--LAV-LAAAGAQAHYTFPGT----KYNG-VAQPQ-WDTVRIT-- 40
glomerrela_graminic__6/1-230    1 M---------KFSAV--LVA-LAAASAEAHYTFGRL----VYGG-TTYPE-WQYVRKT-- 40
arthrobotrys_oligospora_11/1-1  1 M--------------------------------------------------------T-- 2
pyrenophora_trici_repentis_20/  1 M---------KYSLA--ALL-AVASTASAHYTLPEL----TVKG-VKTGQ-WAYVRKT-- 40
Phaeospheraria_nodorum_28/1-22  1 M---------KTAFL--SLL-AAASTASAHYTFPGF----ISGS-TVTSP-WEYVRKT-- 40
myceliophthora_thermophilia_16  1 M---------KLTSS--LAV-LAAAGAQAHYTFPRA----GTGG-SLSGE-WEVVRMT-- 40
thievela_terestis_11/1-231      1 M---------KLTTS--VAL-LAAAGAQAHYTFPQT----DING-QLSGE-WVTIRET-- 40
chaetomium_globosum_24/1-226    1 M---------KFASS--VAL-LAAAGAQAHYTFPKT----VVDG-VTSAE-WETIRIT-- 40
chaetomium_thermophilum_14/1-23 1 M---------KFTTP--LAL-LAVVGVQAHYTFPRT----KVNG-VLSGE-YETVRLT-- 40
TYPE1:NCU02344/1-232            1 M---------KFSSA--LAF-LAAAGAQAHYTFPKG----YSTG-AVSGE-YEHIRMT-- 40
myceliophthora_thermophilia_2/  1 M---------KGLLGAAALS-LAVSDVSAHYIFQQL----TTGG-VKHAV-YQYIRKN-- 42
sodaria_macrospora_4/1-217      1 M---------K-LTVAAAL--LAASEASAHYIFQQV----GTGT-TVNPT-WKYIRQH-- 40
neurospora_tetrasperma_12/1-21  1 M---------K-LSVAAALS-LAASEASAHYIFQQV----GAGT-SVNPV-WKYIRKH-- 41
TYPE1:NCU00836/1-218            1 M---------K-LSVAAALS-LAASEASAHYIFQQV----GAGT-SVNPV-WKYIRKH-- 41
thievela_terestis_7/1-223       1 M---------KGLFSAAALS-LAVGQASAHYIFQQL----SING-NQFPV-YQYIRKN-- 42
podospora_anseria_5/1-221       1 M---------KVFPIVTALT-LGVADVSAHYIFQQF----GVGS-TKFGV-FEHIRKN-- 42
leptosphaeria_maculans_15/1-17  1 ------------------------------------------------------------ 1
glomerrela_graminic_9/1-232     1 M---------RLLNLL-----AAAGFCQAHTIFVSL----DADG-VNSGI-SQGVRTP-- 38
glarea_lozoyensis_6/1-240       1 M---------KTQS-YLSFA-ALLPFASAHTIFSQL----QVGS-TTYPI-SYGIRTP-- 41
TYPE2:NCU02240/1-235            1 M---------KVLSLL-----AAASAASAHTIFVQL----EADG-TTYPV-SYGIRTP-- 38
botryotinia_fuckeliana_2/1-234  1 M---------AMAK-FIVTS-YPESRAVVKAILTKP-----------SDT-SYAIRTP-- 35
botryotinia_fuckeliana_12/1-24  1 M---------KLQL-IIPFS-FLISYVSAHTIFMKL----QSGG-TLYNT-SYAIRTP-- 41
sclerotinia_sclerot_5/1-240     1 M---------KLQF-LIPSS-FLLSYVSAHTIFTQL----ESGG-TLYNT-SYAIRDP-- 41
Phaeospheraria_nodorum_9/1-227  1 M---------KLSTTL-----FFATAASAHTIFVSV----NGGK-V-----GDGVRVP-- 34
chaetomium_thermophilum_18/1-2  1 M---------KLLAPL-----ALVGAASAHTIFVSL----EVNG-VNHGV-GNGVRVP-- 38
cholletotrichum_higginsianum_3  1 M---------KVLLSL-----LTASLASAHTIFSSL----EVGG-VNQGQ-GNGVRIP-- 38
podospora_anseria_30/1-234      1 M---------KFAPI------LLASAASAHTIFSSL----EVNG-VNHGV-GGGVRVP-- 37
myceliophthora_thermophilia_10  1 M---------KVLAPL-----ILAGAASAHTIFSSL----EVGG-VNQGI-GQGVRVP-- 38
neurospora_crassa_1/1-236       1 M---------KVLAPL-----VLASAASAHTIFSSL----EVNG-VNQGL-GEGVRVP-- 38
TYPE2:NCU01050/1-226            1 M---------KVLAPL-----VLASAASAHTIFSSL----EVNG-VNQGL-GEGVRVP-- 38
neurospora_tetrasperma_1/1-236  1 M---------KVLAPL-----VLASAASAHTIFSSL----EVNG-VNQGL-GEGVRVP-- 38
sodaria_macrospora_11/1-236     1 M---------KVLAPL-----VLASAASAHTIFSSL----EVGG-VNQGL-GQGVRVP-- 38
Paravalsa_indica_8/1-210        1 M---------RLAIALVLGA-TYVTGVFGHATFQQL----WVNG-VDQGS--YCVRLP-- 41
pyrenophora_trici_repentis_11/  1 M---------KYATIFL----AAAATVSAHSTWQQL----WVGS-EDKAG--TCVRTV-- 38
leptosphaeria_maculans_11/1-22  1 M---------KAGLILL----TAAASVSAHSTWQDL----WVGS-EDKAT--SCTRTV-- 38
Phaeospheraria_nodorum_14/1-22  1 M---------KSGLVLL----ATVASVSAHSTWQQL----WVGS-DDKAG--TCVRTV-- 38
pyrenochaeta_lyocope_1/1-227    1 M---------KSAIVLL----AAAASVSAHSTWQQL----WVGS-SDKAA--TCVRTV-- 38
pyrenophora_terestis_25/1-227   1 M---------KYATIFL----ATAATVSAHSTWQEL----WVGT-SDKAG--TCVRTV-- 38
glarea_lozoyensis_1/1-214       1 M---------GNASQSL------RKQVSSHPNAQHP----SIPA---------------- 25
myceliophthora_thermophilum_12  1 M---------KSFTLTTLAA-L-AGNAAAHATFQAL----WVDG-VDYGA--QCARLP-- 40
chaetomium_thermophilum_7/1-24  1 M---------KTLSLAALAA-LWAQKAAAHAMFQQL----WVDG-VDYGT--QCARVP-- 41
podospora_anseria_15/1-234      1 M---------KSTFFAALTA-LAAKEIAAHATFQQL---------WHGS--SCARLP-- 36
thievela_terestis_10/1-235      1 M---------RTTFAAALAA-FAAQEVAGHAIFQQL---------WHGS--SCVRMP-- 36
podospora_anseria_24/1-240      1 M---------KSFTATALAA-LLAQQAAAHSTFQQL----WVDG-TDFGS--QCARLP-- 41
pyrenophora_teres_17/1-233      1 M---------KSTFILAGVT-AFATQVAAHATFQDL-----------WS--TCARLP-- 34
pyrenophora_trici_repentis__8/  1 M---------KNTFILVGLI-ALATQVVAHATFQDL-----------WS--TCARLP-- 34
chaetomium_globosum_22/1-239    1 M---------KSFTVATLA--ALAGNAAAHATFQQL----WVDG-VDYGS--QCARVP-- 40
thievela_terestis_17/1-240      1 M---------KSFTIAALAA-LWAQEAAAHATFQDL----WIDG-VDYGS--QCVRLP-- 41
glomerrela_graminic_32/1-232    1 M---------KSIAALALTA-SMANMVAGHAIFQQL----WVNG-KDEAD--SCVRMP-- 41
verticillium_dahliae_23/1-243   1 M---------KYA---ISAL-ALAALSQGHAIFQVV----SVNG-VEAPP-LSGLRAP-- 39
verticilium_albo_atrum_15/1-21  1 M---------R----------NLGAIWEYGSSSEVG----SAPI-SEGPG-SERTPFP-- 33
glomerrela_graminic_17/1-244    1 M---------KLAF--LPAI-AFASMAHAHAIFQKV----SVNG-KEYGS-LAGLRAP-- 40
chaetomium_globosum_23/1-244    1 M---------KLSL--VSLL-GAALSVEGHAIFQKV----SVNG-ADQGS-LTGLRAP-- 40
podospora_anseria_29/1-244      1 M---------KFSF--IALL-ACGLTVDAHAIFQKI----SVNG-QDKGQ-LTGIRAP-- 40
chaetomium_thermophilum_15/1-2  1 M---------KLSL--ASLL-TAALSVQGHAIFQKV----SING-QDHGQ-LTGLRAP-- 40
sodaria_macrospora_16/1-245     1 M---------KISL--LSLI-TYGLAVDAHAIFQKL----SVNG-KDNGQ-LTGIRAP-- 40
myceliophthora_thermophilia_5/  1 M---------KLSL--FSVL-ATALTVEGHAIFQKV----SVNG-ADQGS-LTGLRAP-- 40
thievela_terestis_2/1-244       1 M---------KFSL--VSLL-AYGLSVEAHSIFQRV----SVNG-QDQGL-LTGLRAP-- 40
Paravalsa_indica_13/1-241       1 M---------KWAA--STLL-FAA-AANAHTIFQKV----FVNG-ADQGL-LVGLRAP-- 39
Paravalsa_indica_12/1-241       1 M---------KWAA--STLL-FAA-AANAHTIFQKV----FVNG-ADQGL-LVGLRAP-- 39
myceliophthora_thermophilia_6/  1 M---------KPF----SLV-ALATAVSGHAIFQRV----SVNG-QDQGQ-LKGVRAP-- 38
chaetomium_globosum_31/1-241    1 M---------KAF----SLV-TLATAVSGHAIFQRL----SVNG-QDQGQ-LKGIRAP-- 38
```

```
podospora_anseria_4/1-241        1   M---------KAF----TLV-SLAASVSAHSIFQKV----SVNG-VDQGQ-LKGVRAP--  38
sodaria_macrospora_2/1-240       1   M---------KTG---AILT-ALVASASAHTIFQKV----SVNG-ADQGQ-LKGIRAP--  39
neurospora_tetrasperma_16/1-24   1   M---------KTG---SILA-ALVASASAHTIFQKV----SVNG-ADQGQ-LKGIRAP--  39
neurospora_tetrasperma_14/1-24   1   M---------KTG---SILA-ALVASASAHTIFQKV----SVNG-ADQGQ-LKGIRAP--  39
TYPE2:NCU02916/1-241             1   M---------KTG---SILA-ALVASASAHTIFQKV----SVNG-ADQGQ-LKGIRAP--  39
4EIR/1-223                       1   -----------------------------XTIFSSL----EVNG-VNQGL-GEGVRVP--  23
pyrenophora_trici_repentis_22/   1   M---------KTQSVLLAAL-ASAPAALAHTVFTDF----FVDG-MPQG-----------  34
verticillium_dahiae_24/1-222     1   M---------KTA---VVLG-LLAPFAFGNYLFRSI----TVNR-GRSAD-WEFVRET--  39
verticilium_albo_atrum_16/1-21   1   -----------------MLG-LLAPFAFGNYLFRSI----TVNR-GRSAD-WEFVRET--  34
verticillium_dahliae_26/1-221    1   M---------KSS---ALLP-LLSSGVLAHYFFPNT----VVNG-VQSAE-WEFIRET--  39
3ZUD/1-228                       1   -----------------------------XGFVQNI----VIDG-KNYGG-YLVNQYPY-  24
TYPE3:NCU07898/1-239             1   M---------KT---FATLL-ASIGLVAAHGFVDNA----TIGG-QFYQF-YQPYQDPY-  40
2YET/1-228                       1   -----------------------------XGFVQNI----VIDG-KNYGG-YLVNQYPY-  24
thermoascus_auranti_1/1-228      1   -----------------------------XGFVQNI----VIDG-KNYGG-YLVNQYPY-  24
4EIS/1-224                       1   ----------------------------BHGFVDNA----TIGG-QFYQF-YQPYQDPY-  25
giberella_zeae_7/1-251           1   MA-FQSINSSKA-SFWLTLL-LPALGISAHGHVDEI----IVNG-VSYQG-YGSTDFPY-  50
fusarium_oxysporum_3/1-252       1   MSLLSEMTCFKA-ACWLALL-LPAITVSAHGHVDEI----IING-VSYQG-YGSTDFPY-  51
nectria_heamatococcuss_1/1-252   1   MTFFTAMSTLCA-SAWLYLL-FSAVSVSAHGHVTQV----IING-VAYGG-YLSTSFPL-  51
verticillium_albo_atrum_13/1-24  1   M----------AQKALFAVV-FGAVSAAAHGFVETI----TVNG-KTYDN-YNPSTFPY-  42
verticillium_dahliae_4/1-241     1   M----------AQKALFAVV-LGAVSAAAHGFVETI----TVNG-KTYDN-YNPSTFPY-  42
neurospora_tetrasperma__2/1-24   1   M----------ARKSILTAL-AGASLVAAHGHVSKV----IVNG-VEYQN-YDPTSFPY-  42
TYPE3:NCU07760/1-240             1   M----------ARMSILTAL-AGASLVAAHGHVSKV----IVNG-VEYQN-YDPTSFPY-  42
magna_porte_oryzae_16/1-243      1   M---------KS-AALLAAL-GSAGTVLAHGHVDYI----IVEG-VQYPG-YDVTKYPW-  42
podospora_anseria_31/1-244       1   -------MSNKA-ATLLAAL-SGAALVAAHGHVSHI----IVNG-VYYQN-YDPTTHFY-  44
hypocrea_orientalis_1/1-246      1   -------MIQKLSNLLLTAL-AVATGVVGHGHINNI----VVNG-VYYQG-YDPTSFPY-  45
trichoderma_SP_SSL_1/1-246       1   -------MIQKLSNLLLTAL-AVATGVVGHGHINNI----VVNG-VYYQG-YDPTSFPY-  45
Hypocrea_virens_3/1-246          1   -------MTQKLTSLLVTAL-TVATGVIGHGHVNNI----VING-AYYQG-YDPTLFPY-  45
trichoderma_atroviride_2/1-246   1   -------MAQKLSNLFAIAL-TVATGVVGHGHVNNI----VVNG-VYYQG-YDPTSFPY-  45
hypocrea_rufa_1/1-246            1   -------MIQKLSNLLVTAL-AVATGVVGHGHINDI----VING-VWYQA-YDPTTFPY-  45
hypocrea_rufa_2/1-246            1   -------MIQKLSNLLVTAL-AVATGVVGHGHINDI----VING-VWYQA-YDPTTFPY-  45
trichoderma_saturnusporum_1/1-   1   -------MIQKLSNLLVAAL-TVATGVVGHGHINNI----VING-VYYQA-YDPTSFPY-  45
aspergillus_kawachii_37/1-247    1   -------MRQAQSASLLAAL-LSATQVAAHGHVTNL----VVDG-VYYEG-FDISVFPY-  45
aspergillus_tereus_6/1-245       1   -------MDRLSKSTLLALL---ASQVAGHGHVTNI----VING-VSYQG-WDINSFPY-  43
neosartorya_fischeri_4/1-247     1   -------MRHVQSTQLLAAL-LFTTHVTAHGHVTNI----VVNG-VSYRG-WNIDSDPY-  45
aspergillus_fumingatus_4/1-247   1   -------MRHVQSTQLLAAL-LLTTRVTAHGHVTNI----VING-VSYRG-WNIDSDPY-  45
aspergillus_tereus_10/1-245      1   -------MHTLQSAILLGGL--LATQVAAHGHVTNI----VING-VYYRG-WNIDSDPY-  44
chaetomium_globosum_5/1-220      1   -------MPSYTSKTLLSAL-AGAVSVAAHGHVTNI----VING-VSYEG-FDPTSFPY-  45
TYPE3:NCU05969/1-243             1   -------MPSFTSKSLLAVL-AGAASVAAHGHVSNI----VING-EYYRG--FDSSLNY-  44
2VTC/1-228                       1   -----------------------------HGQVQNF----TING-QYNQG-FIL-DYYYQ  24
aspergillus_fumingatus_1/1-245   1   M---------SVPKIAAALL-SSAALVAGHGFVTGA----VVDG-KYYTG-YLVNQYPY-  43
neosartorya_fischeri_1/1-245     1   M---------SVSKIAAVLL-SSAALVAGHGFVSGA----VVDG-KYYTG-YLVNQYPY-  43
aspergillus_niger_1/1-245        1   M---------PLSKIAGVLL-ASASLVAGHGYVSSI----EVDG-TTYGG-YLVDTYYY-  43
aspergillus_kawachii_40/1-245    1   M---------SLSKIAGVLL-ASASLVAGHGYVSSI----EVDG-TTYGG-YLVDTYYY-  43
aspergillus_tereus_5/1-232       1   M---------SLSKIATGIL-ASATLVAGHGYVSGI----VADG-KYYSG-YLVDKYSY-  43
emmericella_nidulans_9/1-245     1   M---------SVARTAGFAL-ASAAIVAGHGYVTGI----VADG-TYYGG-YLVNQYPY-  43
penicillum_chrysogenum_2/1-245   1   M---------SVSKIAGLML-SSAAMVAGHGFVSGA----VVDG-TYHGG-YLVNNYPY-  43
aspergillus_niger_2/1-245        1   M---------SVAKIAGVVL-GSAALVAGHGYVSGA----VIDG-EYYGG-YIVSSYPY-  43
aspergillus_niger_12/1-244       1   M---------SVAKIAGVVL-GSAALVAGHGYVSGA----VIDG-EYYGG-YIVSSYAY-  43
aspergillus_kawachii_38/1-245    1   M---------SVAKIAGVVL-GSAALVAGHGYVSGA----VVDG-QYYGG-YIVSSYAY-  43
zea_mys_1/1-245                  1   M---------SVAKIAGVVL-GSAALVAGHGYVSGA----VVDG-QYYGG-YIVTSYAY-  43
aspergillus_clavatus_6/1-241     1   M---------SVTKIAGILL-GSAAMVAGHGFVTGA----VVDG-TYHTG-YLVNNYPY-  43
aspergillus_tereus_8/1-241       1   M---------SVAKIAGVVL-GSAALVAGHGYVTGA----VVDG-KYYAG-YDVTSAPY-  43
aspergilus_oryzae_7/1-242        1   M---------SIAKIAGVVL-GSAALVAGHGYVSGA----VVDG-QYYSG-YDM-SYHY-  42
aspergillus_favus_5/1-242        1   M---------SIAKIAGIVL-GSAALVAAHGHVSGA----VVDG-QYYPG-YDI-SYHY-  42
glomerrela_graminic_4/1-242      1   -------MSSFKNSIVLSAL-AGAVSVMAHGHVVSF----TDGS-QEFSG-YDVTKMAY-  45
aspergillus_fumingatus_3/1-238   1   M---------KLS--LLASV-ALVPFVSAHYFFDVL----VIDG-KETRS-NEFVRSNT-  41
cholletotrichum_higginsianum_2   1   M---------KFS--AVVLA-AIAPLVSAHYFFDTL----IVDG-KATKS-FEYVRSNT-  41
glomerrela_graminic_7/1-238      1   M---------KFS--AVALS-AVAPLASAHYFFDSF----IVDG-VATKS-FEYVRDNT-  41
aspergillus_clavatus_5/1-238     1   M---------KLS--VITAV-ALVPFVSAHYFFDKL----IVNG-KETAS-FQYVRSNT-  41
aspergilus_oryzae_5/1-238        1   M---------KLS--FLALA-AIAPFVSAHYFFDTL----IVDG-KESSP-NQYVRSNT-  41
aspergillus_favus_3/1-238        1   M---------KLS--FLALA-AIAPFVSAHYFFDTL----IVDG-KESSP-NQYVRSNT-  41
aspergillus_niger_9/1-238        1   M---------KLT--LLTTA-LIAPLVSAHYFFDTL----VIDG-QETTP-NQYVRSNT-  41
emmericella_nidulans__6/1-238    1   M---------KLS--LLAAA-AIAPMVSAHYFFDTL----VIDG-QETTP-NQYVRSNT-  41
aspergillus_clavatus_1/1-238     1   M---------KLS--LLALS-AIAPLVSAHYFFDVL----VIDG-KETKS-NEFVRSNT-  41
neosartorya_fischeri_7/1-238     1   M---------KLS--LLAAV-ALVPFVSAHYFFDVL----VIDG-QETKS-NEFVRSNT-  41
penicillum_chrysogenum_4/1-236   1   M---------KFS--LVALA-AIAPMVSAHYFFDTI----VIDG-KEAS---DSVRSNT-  39
verticillium_albo_atrum_17/1-21  1   M---------RFS--ILAVA-ALTPLASAHYFFDKL----IIDG-VETRS-NEFVRSNT-  41
verticillium_albo_atrum_17/1-2   1   M---------NFC--ILAAA-ALTPLASAHYFFDKL----IIDD-VETRS-NEFVRSST-  41
pyrenophora_teres_22/1-230       1   M---------KAV--ALFSL-LAPMVADAHYIFSSL----IVNG-AQVGGDFAYARKNS-  42
Phaeospheraria_nodorum_4/1-230   1   --------MVKAT--AVLAL-LAPL-TQAHYIFNQL----LVDN-KAIGGDYAYTRKNT-  42
pyrenophora_trici_repentis_23/   1   M---------KAV--ALFAL-VAPMVADAHYIFNLL----TV------------------  26
podospora_anseria_17/1-231       1   M---------KLS-SFTILA-GLAAQAQAHYIFNIL----IVNG-QRIGGEYTYVRRNS-  43
glomerrela_graminic_16/1-230     1   M---------RISTFLPVLA---VPFVQCHYIFSQL----IVNG-NAVGSDYTYMRKNS-  42
glomerrela_graminic_31/1-230     1   M---------KGLLSFGLLA---APLVQGHYIFSQL----FVDD-KAAGGDYTYIRKNT-  42
chaetomium_globosum_15/1-229     1   M---------K---SFFVAA-ALAASAEAHYIFNIL----MVND-QRMGGEYTYVRRNS-  41
neosartorya_fischeri_2/1-235     1   M---------KLTSSILFSLASVTPLVSGHYVFSKL----IVDG-KPTQD-FEYIRKNT-  44
aspergillus_tereus_12/1-235      1   M---------KSFSPSLLLLASVAPLVSGHYVFSKF----LVDG-SVSQD-FEYIRRNS-  44
```

```
aspergillus_favus_7/1-235          1   M---------KLNLASLSFLASIAPLVSGHYVFSKL----IVDG-QTTKD-FEYIRENS- 44
aspergillus_fumingatus_6/1-235     1   M---------KLTASILFSLASVTPLVSGHYVFSKL----IVDG-KPTQD-FEYIRRNT- 44
4B5Q/1-217                         1   -----------------------------HYTFPDF----IEPSGTVTGD-WVYVRET-- 24
3EJA/1-208                         1   -----------------------------HYTWPRV----NDGA-DW-----QQVRKA-- 19
3EII/1-208                         1   -----------------------------HYTWPRV----NDGA-DW-----QQVRKA-- 19

                                                70        80        90       100       110       120
                                       ....|....|....|....|....|....|....|....|....|....|....|....|
serpula_lacrymans_5/1-229          40  ---------------QNTNDNS--PVTDVTSTDLRCNVGGLA-SGATTSTYTVSAGSVVG 82
schizzophylum_commune_15/1-228     39  ---------------RTNNYYSQQPVTDVTSPDFTCYTTETQ---ATAETAEVAAGSSVS 81
schizzophylum_commune_16/1-228     39  ------------ATATTAEVAAGSSVSIKANGP----------------MYHAGVVNVYM 71
chaetomium_globosum_8/1-223        36  ---------------DNWQNNG--FVGDVNSAQIRCFQSSSA---GAQDVYTVSAGSTVK 76
pyrenophora_trici_repentis_13/     36  ---------------KNWQDNG--FVGDVTSSDIRCNQLRP----GTSGALSVAAGSSVK 75
pyrenophora_teres_11/1-209         36  ---------------KNWQDNG--FVGDVTSSDIRCNQLRS----GTSGALSVAAGSSVK 75
Phaeospheraria_nodorum_18/1-21     35  ---------------KNWQDNG--FVGDVTSSAIRCNQLSP----GKS-TLSVAAGSSVK 73
podospora_anseria_18/1-223         36  ---------------DNWQNNG--FVGSVTSPQIRCFQNSVA---GASQTYNVSAGSQLT 76
thievela_terestis_18/1-224         37  ---------------DNWQDNG--YVGDVTSPQIRCFQATPS---PAPSVLNTTAGSTVT 77
myceliophthora_thermophilia_21     36  ---------------DNWQNNG--FVGDVNSEQIRCFQATPA---GAQDVYTVQAGSTVT 76
TYPE1:NCU03328/1-229               40  ---------------DNWQNNG--FVDNVNSQQIRCFQSTHS---PAQSTLSVAAGTTIT 80
emmericella_nidulans__3/1-229      41  ---------------TGSYGNG--PVEDVTSLDIRCNKDAST-NGNATETLPVKAGEEIG 83
aspergillus_tereus_4/1-228         41  ---------------TGSYTNS--PVTDVDSLDIRCNVDATT--GNNTSTLGVAAGSTIG 82
podospora_anseria_11/1-231         40  ---------------QNHYSNG--PVTDVNSPLMTCYERDPG--VGAPNTLAVTAGSTVT 81
glomerrela_graminic__6/1-230       40  ---------------LNFYSNG--PTDGVSSTQIRCYEADAKD-RGTVQTLPVTAGSTIG 82
arthrobotrys_oligospora_11/1-1     2   ---------------TNKYSHG--PVTDVTSSDMRCYK-DPA--APIASTANVAAGSQVG 42
pyrenophora_trici_repentis_20/     40  ---------------SNYQSNG--PVTDVTSNAIRCYELSPG---TGSKTYTVNAGDTVG 80
Phaeospheraria_nodorum_28/1-22     40  -----------------TNSNG--PVTDVTSNQIRCYELSPG---TGSKTYTVNAGDTVG 78
myceliophthora_thermophilia_16     40  ---------------ENHYSHG--PVTDVTSPEMTCYQSGV---QGAPQTVQVKAGSQFT 80
thievela_terestis_11/1-231         40  ---------------TNHYSHG--PVTDVTSDQIRCYELNPG--TPAPQIATVQAGGTVT 81
chaetomium_globosum_24/1-226       40  ---------------ENHYSHG--PVEDVSSSKMTCYQRDVG--TGAAKTVSVKAGGTVG 81
chaetomium_thermohilum_14/1-23     40  ---------------ANHWSHG--PVTDVTSQEMTCFEKNPG--TPAPKTITVQAGNNVT 81
TYPE1:NCU02344/1-232               40  ---------------ENHYNRG--PVADVTSESMTCYELNPG--KGAPKTLSVAAGSNYT 81
myceliophthora_thermophilia_2/     42  -----------------TNYNS--PVTDLTSNDLRCNVGATG---AGTDTVTVRAGDSFT 80
sodaria_macrospora_4/1-217         40  -----------------TNYNS--PVTDLTSNDLRCNVGASA---SGVETLSVAAGGTVT 78
neurospora_tetrasperma_12/1-21     41  -----------------TNYNS--PVTDLTSKDLVCNVGASA---EGVETLSVAAGSQVT 79
TYPE1:NCU00836/1-218               41  -----------------TNYNS--PVTDLTSKDLVCNVGASA---EGVETLSVAAGSQVT 79
thievela_terestis_7/1-223          42  -----------------TNYNS--PVTDLTSDDLRCNVGAQG---AGTDTVTVKAGDQFT 80
podospora_anseria_5/1-221          42  -----------------NNHNS--PVTSLSDNNLRCNVGGAS--GASTSVVNVKAGDSVT 81
leptosphaeria_maculans_15/1-17     1   -------------------------------------------MPLPKDILNVKAGDKLS 17
glomerrela_graminic_9/1-232        38  ------------------DYDG--PQTDVTSQYIACNGPPNPTK-PTDKVITVTAGSTVT 77
glarea_lozoyensis_6/1-240          41  ------------------NYDG--PINDVSSKDVVCNGGPNPTT-PSSKIIDVKAGDTVK 80
TYPE2:NCU02240/1-235               38  ------------------SYDG--PITDVTSNDLACNGGPNPTT-PSDKIITVNAGSTVK 77
botryotinia_fuckeliana_2/1-234     35  ------------------TYDG--PINDVTTEYVACNGGPNPTT-PSSNIINVVAGSTVN 74
botryotinia_fuckeliana_12/1-24     41  ------------------TYDG--PINDVTTEYVACNGGPNPTT-PSSNIINVVAGSTVN 80
sclerotinia_sclerot_5/1-240        41  ------------------TYDG--PITDVTTQYVACNGGPNPTT-PSSNIINVVAGSTVK 80
Phaeospheraria_nodorum_9/1-227     34  ------------------SYDG--PINDVSSNDIVCNGGPNPTA-STSTVITLQAGSSAT 73
chaetomium_thermophilum_18/1-2     38  ------------------SYNG--PIEDVTSNSIACNGPPNPTT-PTDKVITVQAGSEVT 77
cholletotrichum_higginsianum_3     38  ------------------SYNG--PIEDVTSNSLACNGPPNPTS-PTSKVITVQAGQNVT 77
podospora_anseria_30/1-234         37  ------------------SYNG--PIENVDSASIACNGAPNPTT-PTSKVITVQAGQNVT 76
myceliophthora_thermophilia_10     38  ------------------SYNG--PIEDVTSNSIACNGPPNPTT-PTNKVITVRAGETVT 77
neurospora_crassa_1/1-236          38  ------------------TYNG--PIEDVTSASIACNGSPNTVA-STSKVITVQAGTNVT 77
TYPE2:NCU01050/1-226               38  ------------------TYNG--PIEDVTSASIACNGSPNTVA-STSKVITVQAGTNVT 77
neurospora_tetrasperma_1/1-236     38  ------------------TYNG--PIEDVTSASIACNGSPNTVA-STSKVIIVQAGTNVT 77
sodaria_macrospora_11/1-236        38  ------------------TYNG--PIEDVTSASIACNGSPNTVG-STSKVITVQAGTNVT 77
Paravalsa_indica_8/1-210           41  ------------------QNNN--PVTSVTSNDIACNAGAA----SSSGLCSVKPGDKVT 77
pyrenophora_trici_repentis_11/     38  ------------------KDNS--PITSITSSDMFCGRGPA----STSGVCEVAAGSSLT 74
leptosphaeria_maculans_11/1-22     38  ------------------ANNN--PISSLTSADMFCGIGPA----KSSGVCEVAAGSALT 74
Phaeospheraria_nodorum_14/1-22     38  ------------------KDNS--PIASLTSTDMFCGRGPA----TSSGVCEVAAGGSLT 74
pyrenochaeta_lyocope_1/1-227       38  ------------------KDNS--PIESLTSADMFCGRGPA----ASSGVCEVAAGSALT 74
pyrenophora_terestis_25/1-227      38  ------------------KDNS--PITSLTSPDMFCGRGPA----STSGVCEVAAGDSLT 74
glarea_lozoyensis_1/1-214          25  ---------------------A--PVTSVSSNDIRCNAGTK----PVTPLCTVPAGGTIT 58
myceliophthora_thermophilum_12     40  ------------------ASNS--PVTDVTSNAIRCNANPS----PARGKCPVKAGSTVT 76
chaetomium_thermophilum_7/1-24     41  ------------------GSNS--PVTNVNSPEIRCNAYPS----PAKGKCPVKAGSIVT 77
podospora_anseria_15/1-234         36  ------------------PSNS--PVTNVGGRDFVCNAGTR----GVAGKCPVRAGGTVT 72
thievela_terestis_10/1-235         36  ------------------LSNS--PVTNVGSRDMICNAGTR----PVSGKCPVKAGGTVT 72
podospora_anseria_24/1-240         41  ------------------QSNS--PITNYNSNDMRCNIIGT----RPQVKCPVRAGGTVT 77
pyrenophora_teres_17/1-233         34  ------------------NSNS--PVQSVTSNDIRCNANQG----PAASKCSVPAGGTVT 70
pyrenophora_trici_repentis__8/     34  ------------------NSNS--PVQSVTSNDIRCNANQG----PAASKCSVPAGGTVT 70
chaetomium_globosum_22/1-239       40  ------------------SSNS--PITDVSGTGVRCNVGTS----RPSGKCPVKAGSTVT 76
thievela_terestis_17/1-240         41  ------------------ASNS--PVTNVASDDIRCNVGTS----RPTVKCPVKAGSTVT 77
glomerrela_graminic_32/1-232       41  ------------------ASNS--PIQDPTSDDMRCNANAA----KAASTCAVKAGDVVT 77
verticillium_dahliae_23/1-243      39  ------------------GINN--PVEDVSSNDLTCGLVTH----TSTDVIEVEAGDEIG 75
verticilium_albo_atrum_15/1-21     33  ------------------RRKA--PVQKAPNH--------------------FEAGDEIG 53
glomerrela_graminic_17/1-244       40  ------------------NQNN--PTQDVTSQDIICGKVAT----SSQEVISVAPGDRIG 76
chaetomium_globosum_23/1-244       40  ------------------NNNN--PVQDVSSQNMICGQPGS----TSQTVIQVKAGDRIG 76
podospora_anseria_29/1-244         40  ------------------NNNN--PVQNVNDQNMACGQPGS----KSNTVVNVNAGDRIG 76
chaetomium_thermophilum_15/1-2     40  ------------------NTNY--PVEDVNSQDIICGQSGH----RSSTILNVRAGDRIG 76
sodaria_macrospora_16/1-245        40  ------------------GNNN--PVQDVSSSNMACGASGT----KSQTVINVAPGDRLG 76
```

```
myceliophthora_thermophilia_5/   40   ------------------NNNN--PVQNVNSQDMICGQSGS----TSNTIIEVKAGDRIG 76
thievela_terestis_2/1-244        40   ------------------SNNN--PVQDVNSQNMICGQSGS----KSQTVINVKAGDRIG 76
Paravalsa_indica_13/1-241        39   ------------------SNNN--PIQSTSDSGLACGASGS----KSSTVINVKAGDTIG 75
Paravalsa_indica_12/1-241        39   ------------------SNNN--PIQSTSDSGLACGASGS----KSSTVINVKAGDTIG 75
myceliophthora_thermophilia_6/   38   ------------------SSNS--PIQNVNDANMACNANIV---YHDNTIIKVPAGARVG 75
chaetomium_globosum_31/1-241     38   ------------------YSNY--PIQNVNDANIACNANIQ---IKDNNIIKIPAGARVG 75
podospora_anseria_4/1-241        38   ------------------YSNF--PIENVNHPDFACNTNIQ---LRDNTVIKVPAGARVG 75
sodaria_macrospora_2/1-240       39   ------------------PNNN--PVLNVQSGDIACNVAS----IKDSNVLTVPAGAKVG 75
neurospora_tetrasperma_16/1-24   39   ------------------ANNN--PVTDVMSSDIICNAVT----MKDSNVLTVPAGAKVG 75
neurospora_tetrasperma_14/1-24   39   ------------------ANNN--PVTDVMSSDIICNAVT----MKDSNVLTVPAGAKVG 75
TYPE2:NCU02916/1-241             39   ------------------ANNN--PVTDVMSSDIICNAVT----MKDSNVLTVPAGAKVG 75
4EIR/1-223                       23   ------------------TYNG--PIEDVTSASIACNGSPNTVA-STSKVITVQAGTNVT 62
pyrenophora_trici_repentis_22/   34   -------------------------------DGVAMHVGGTK---GVSRVQSVPDGALLT 60
verticillium_dahiae_24/1-222     39   ---------------ANNPVSN--PIEDVSSPLLGCYEKVGR---PVSDVQTVAPGTRIR 79
verticilium_albo_atrum_16/1-21   34   ---------------ANNPASN--PIEDVSSPLLSCYEKIGR---PVSAVQTVAPGTRIR 74
verticillium_dahliae_26/1-221    39   ---------------NNNPGAE--PVEDLSSTFLRCFEKPGR---PPSAVLPVTAGSTVG 79
3ZUD/1-228                       24   ---MSNPPEVIAWSTT-ATDLGFVDGTGYQTPDIICHRGAK----PGALTAPVSPGGTVE 76
TYPE3:NCU07898/1-239             40   ---MGSPPDRISRK-----IPGNGPVEDVTSLAIQCNADSA----PAKLHASAAAGSTVT 88
2YET/1-228                       24   ---MSNPPEVIAWSTT-ATDLGFVDGTGYQTPDIICHRGAK----PGALTAPVSPGGTVE 76
thermoascus_auranti_1/1-228      24   ---MSNPPEVIAWSTT-ATDLGFVDGTGYQTPDIICHRGAK----PGALTAPVSPGGTVE 76
4EIS/1-224                       25   ---MGSPPDRISRK-----IPGNGPVEDVTSLAIQCNADSA----PAKLHASAAAGSTVT 73
giberella_zeae_7/1-251           50   ---MQDPPVVAGWTIE-QADNGFVSPDKYDDPDIICHRDAT----PAKGHIELAAGDTLT 102
fusarium_oxysporum_3/1-252       51   ---MENPPVVAGWTIS-QRDNGFVSPEAYGDPDIICHRDAT----PAEGHIEVTAGDVIT 103
nectria_heamatococcuss_1/1-252   51   ---QRKPPVVLGWTIE-QRDNGFVSPDKYDHPDIICHRDAT----PAQGHVQVAAGDTIT 103
verticilium_albo_atrum_13/1-24   42   ---NPSPPIVPGWTAD-FPDLGFVEPAATGDPDIICHRSAT----NGGSHIPLAAGDTLT 94
verticillium_dahliae_4/1-241     42   ---NPSPPVVPGWTAD-FPDLGFVEPAATGDPDIICHRSAT----NGGSHIPVAAGDTLT 94
neurospora_tetrasperma__2/1-24   42   ---NSNPPTVIGWTID-QKDNGFVSPDAFDSGDIICHKSAT----PAGGHATVKAGDKIS 94
TYPE3:NCU07760/1-240             42   ---NSNPPTVIGWTID-QKDNGFVSPDAFDSGDIICHKSAK----PAGGHATVKAGDKIS 94
magna_porte_oryzae_16/1-243      42   ---QSQQPTVVGWSAT-NTDNGFVEPNNFGSPDIICHRGAQ----PAKGHARVKAGDRIL 94
podospora_anseria_31/1-244       44   ---QPNPPTVIGWSAL-QQDNGFVEPNNFGTTDIICHKSAA----PGGGSATVNAGDKIS 96
hypocrea_orientalis_1/1-246      45   ---ESDPPIVVGWTAA-DLDNGFVSPDAYQSPDIICHKNAT----NAKGHASVKAGDTIL 97
trichoderma_SP_SSL_1/1-246       45   ---ESDPPIVVGWTAA-DLDNGFVSPDAYQSPDIICHKNAT----NAKGHASVKAGDTIP 97
Hypocrea_virens_3/1-246          45   ---EPNPPIVVGWTAS-DTDNGFVAPDAYQSPDIICHRNAT----NARGHASVMAGSSVL 97
trichoderma_atroviride_2/1-246   45   ---MPDPPIVVGWTAA-DTDNGFVSPDAYQTPDIVCHKNGT----NAKGHASVKAGDSVL 97
hypocrea_rufa_1/1-246            45   ---ESNPPIVVGWTAA-DLDNGFVSPDAYQNPDIICHKNAT----NAKGHASVKARDTIL 97
hypocrea_rufa_2/1-246            45   ---ESNPPIVVGWTAA-DLDNGFVSPDAYQNPDIICHKNAT----NAKGHASVKAGDTIL 97
trichoderma_saturnusporum_1/1-   45   ---ESNPPIVVGWTAA-DLDNGFVSPDAYGSPDIICHKNAT----NAKGHASVRAGDTVL 97
aspergillus_kawachii_37/1-247    45   ---ESDPPKVAAWTTP-NTGNGFISPDAYRDPNIICHENAT----NAQAHVVVGAGEKIN 97
aspergillus_tereus_6/1-245       43   ---MADPPTVVAWGTP-NTGNGFIAPDAFTSDDMICHLNAT----NAKGFATVAAGDSIN 95
neosartorya_fischeri_4/1-247     45   ---NPNPPVVVAWQTP-NTANGFISPDAYGTNDIICHLNAT----NARGHAVVAAGDKIS 97
aspergillus_fumingatus_4/1-247   45   ---NPDPPVVVAWQTP-NTANGFISPDAYGTNDIICHLNAT----NARGHAVVAAGDKIS 97
aspergillus_tereus_10/1-245      44   ---NSNPPLVAAWRTP-NTANGFIAPDAFGTSDIICHLNAL----NGQGHIQVAAGDRIS 96
chaetomium_globosum_5/1-220      45   ---NPNPPTVVGWTEG-NSDNGFVAPDAFGNGDIICHKSAT----NAGGHAVVAAGDSVF 97
TYPE3:NCU05969/1-243             44   ---MANPPAVVGWKAN-NQDNGFVGPDAFSSPDIICHKDAT----NAKGHAVVKAGDKIS 96
2VTC/1-228                       25   KQNTGHFPNVAGWYAE-DLDLGFISPDQYTTPDIVCHKNAA----PGAISATAAAGSNIV 79
aspergillus_fumingatus_1/1-245   43   ---MSSPPDSIGWSET-ATDLGFVDGSGYSSGDIICHKDAK----NGAISAEIKAGGKVE 95
neosartorya_fischeri_1/1-245     43   ---MSSPPESIGWSET-ATDLGFVDGSGYSSGDIICHKSAK----NGAISAEIKAGGKVE 95
aspergillus_niger_1/1-245        43   ---ESDPPELIAWSTN-ATDDGYVSPTQYDSSNIVCHRGSA----PGALAAPVTPGGTVK 95
aspergillus_kawachii_40/1-245    43   ---ESDPPELIAWSTN-ATDDGYVSPTQYESSNIVCHRGSA----PGALEAPVAPGGTVK 95
aspergillus_tereus_5/1-232       43   ---MNDPPETIGWSTT-ATDLGFVDGTGYDTVDIACHKGSA----PGALTATVPAGSKIE 95
emmericella_nidulans_9/1-245     43   ---SNDPPAVVGWAED-ATDLGFVDGSGYTSGDIICHKDAT----NAQASATVAAGGTVE 95
penicillum_chrysogenum_2/1-245   43   ---NDNHPETIGWAEK-ATDLGFVDGSGYSGPDIICHKEAT----PGAISAEVKAGGDVE 95
aspergillus_niger_2/1-245        43   ---ESDPPETIAWSTT-ATDLGFVDGSEYSDPDIICHKSAK----PGAISADVKAGGTVE 95
aspergillus_niger_12/1-244       43   ---ESDPPETIAWSTT-ATDLGFVDGSEYSDPDIICHKSAK----PGAISADVKAGGTVE 95
aspergillus_kawachii_38/1-245    43   ---ESDPPETIAWSTE-ATDLGFVDGSEYAEPDIICHKSAK----PGAISADVKAGGTVE 95
zea_mys_1/1-245                  43   ---ESDPPETIAWSTE-ATDLGFVDGSEYAEADIICHKSAK----PGAISADVKAGGTVE 95
aspergillus_clavatus_6/1-241     43   ---SSNPPKTIGWSET-ATDLGFVDGTGYASGDIICHKNAK----PGALSADIKAGGKVE 95
aspergillus_tereus_8/1-241       43   ---SSDPPATIGWSTT-ATDLGFVDGTEYSEPDIICHKDAK----PGSLSAEITAGGKVE 95
aspergilus_oryzae_7/1-242        42   ---MSNPPQVIGWSTD-ATDLGFVDGSSYADADIICHKNAK----NGAISAEIAAGKQVE 94
aspergillus_favus_5/1-242        42   ---MPDPPKVIGWSTD-ATDNGFVDGSSYADADIICHKNAK----NGAISAEIAAGKQVE 94
glomerrela_graminic_4/1-242      45   ---MNPAPEVYGWSET-ATDNGFVAPSAYTDADIICHRGAK----NAALTAKVAAGDKLT 97
aspergillus_fumingatus_3/1-238   41   ---RPAKYNPTK-----WKNIR--DDMTPDMPDFRCNKGAF-TFAGQTGTAEVKAGSKLA 90
cholletotrichum_higginsianum_2   41   ---RQAKYNPTK-----WENVR--DGMTPDLPDFRCNKGAF-TFAAKTGTLEVKAGSKVA 90
glomerrela_graminic_7/1-238      41   ---RQAKYNPTK-----WENVR--DDMTPDVNDFRCNKGAF-TFAGKTRTMEVKAGSKVG 90
aspergillus_clavatus_5/1-238     41   ---RPNKYNPTK-----WNNVR--DNMTPDMSDFRCNKGAF-TFAGKTGVAEIKAGSKVA 90
aspergilus_oryzae_5/1-238        41   ---RPAKYNPTK-----WVNTR--DDMTPDMPDFRCNKGSF-TFAGQTGTAEVKAGSKLA 90
aspergillus_favus_3/1-238        41   ---RPAKYNPTK-----WVNTR--DDMTPDMPDFRCNKGSF-TFAGQTGTAEVKAGSKLA 90
aspergillus_niger_9/1-238        41   ---RPAKYNPTK-----WKNTR--DDMTPDMPDFRCNKGSF-TFAGQTGTAEVKAGSKLA 90
emmericella_nidulans__6/1-238    41   ---RPEKYNPTK-----WVNTR--DDMTPDMPDFRCNKGSF-TFAGQTDTAEVKAGSKLA 90
aspergillus_clavatus_1/1-238     41   ---RPAKYNPTK-----WVNIR--DDMTPDVTDFRCNKGAF-TFAGKTGTAEVKAGSKLA 90
neosartorya_fischeri_7/1-238     41   ---RQAKYNPTK-----WKNIR--DNMTPDMTDFRCNKGAF-TFAGKTGTAEVKAGSKLA 90
penicillum_chrysogenum_4/1-236   39   ---RPAKYNPTK-----WVNTR--DDMTPDMPDFRCNKGAF-TFAGQTGTAEVKAGSKVA 88
verticilium_albo_atrum_17/1-21   41   ---RPAKYNPTK-----WENVR--DDMTPDVNDFRCNKGAF-TFAGQTGTAEVKAGSKLA 90
verticillium_albo_atrum_17/1-2   41   ---PAAKYNPTK-----WENIR--DDMTPDVNDFRCKKGAF-TFASQTGTAEVKAGSKLA 90
pyrenophora_teres_22/1-230       42   ---NSYM------------PSF--TSEIVNSPELRCNKGAKP---GSTGTYTVKAGDKVG 82
Phaeospheraria_nodorum_4/1-230   42   ---NTYM------------PSF--TSEIVNSPDLRCNKGAVA---GNTATYTVKAGQTIG 82
pyrenophora_trici_repentis_23/   26   ---NNYM------------PSF--TSEIVNSPELRCNKGAKP---GSTGTQTVKAGDKIG 66
```

```
podospora_anseria_17/1-231        43  ---NSYN------------PAF---PDILTSDELRCNRGAKPG--GNVQTYEVKAGDKIG 83
glomerrela_graminic_16/1-230      42  ---NNYQ------------PSF--TSEIVNSPELRCNKGATT---GTAQTYTVAAGSKVG 82
glomerrela_graminic_31/1-230      42  ---NTYM------------PSF--TSEIVNSPELRCNKGATS---ATAQTMTVAAGSKLG 82
chaetomium_globosum_15/1-229      41  ---NSYM------------PTF---PDIVTSNDLRCNVGAKPG--GGVQTATVKAGDKIG 81
neosartorya_fischeri_2/1-235      44  ---NGY-------------MPT--LPSDILSNDFRCNKGSM-QSAASTKVYTVAPGTELG 85
aspergillus_tereus_12/1-235       44  ---NGY-------------MPT--LAGDILSNDFRCNQGSM-DSAASTKVYTVAPGTEIG 85
aspergillus_favus_7/1-235         44  ---NGY-------------QPT--LASEIVNNDFRCNKGSM-DSAAKTKVYTVAPGAEIG 85
aspergillus_fumingatus_6/1-235    44  ---NNY-------------MPT--LPSEILSNDFRCNKGSM-QSAANTKVYKVAPGTELG 85
4B5Q/1-217                        24  ---------------QNHYSNG--PVTDVTSPEFRCYELDLQNTAGQTQTATVSAGDTVG 67
3EJA/1-208                        19  ---------------DNWQDNG--YVGDVTSPQIRCFQATPS---PAPSVLNTTAGSTVT 59
3EII/1-208                        19  ---------------DNWQDNG--YVGDVTSPQIRCFQATPS---PAPSVLNTTAGSTVT 59

                                               130       140       150       160       170       180
                                      ....|....|....|....|....|....|....|....|....|....|....|....|
serpula_lacrymans_5/1-229         83  LALDQA----------------IYHPGVVNIYMAKAPANVS---AWDGSGAVWFKVHEVT 123
schizzophylum_commune_15/1-228    82  IKANGP----------------MYHQGVVNVYMADADGDAA---SYDGSGEKWFKVYEIP 122
schizzophylum_commune_16/1-228    72  A---RTNNYNTQAPVQDVTSPDFTCYTSETH---SANPDAA---SYDGSGQKWFKVYEIP 122
chaetomium_globosum_8/1-223       77  YSANPN----------------IYHPGPMQFYLARVPDGQDV-KSWTGDGAVWFKVYHEQ 119
pyrenophora_trici_repentis_13/    76  VSVNPN----------------AYHPGPFQSYLAKVPEGQDV-NTWDPTGAVWFRIYAEQ 118
pyrenophora_teres_11/1-209        76  VSVNPN----------------AYHPGPFQSYLAKVPEGQDV-NTWDPTGAVWFRIYAEQ 118
Phaeospheraria_nodorum_18/1-21    74  VNVNPN----------------AYHPGPFQSYLAKMPDGQDI-NTWDPTGAVWFRIYAEQ 116
podospora_anseria_18/1-223        77  YYVNPN----------------AYHPGPMQFYLARVPDGQDV-TRWDGSGAVWFKIYHEQ 119
thievela_terestis_18/1-224        78  YWANPD----------------VYHPGPVQFYMARVPDGEDI-NSWNGDGAVWFKVYEDH 120
myceliophthora_thermophilia_21    77  YHANPS----------------IYHPGPMQFYLARVPDGQDV-KSWTGEGAVWFKVYEEQ 119
TYPE1:NCU03328/1-229              81  YGAAPS----------------VYHPGPMQFYLARVPDGQDI-NSWTGEGAVWFKIYHEQ 123
emmericella_nidulans__3/1-229     84  FTVRTN----------------IGHPGPLLAYMAKAPGDAS---DFDGDGQVWFKIYEDG 124
aspergillus_tereus_4/1-228        83  FTVKTE----------------IGHPGPLLVYMAKAPTTAA---AFDGSGESWFKIYEDG 123
podospora_anseria_11/1-231        82  FQVGSS----------------VGHPGPLHFYMAKVPAGKTA-KTFDGKGAVWFKIYQDG 124
glomerrela_graminic__6/1-230      83  FAANSN----------------VGHPGPASIWMAKVPAGQTA-ATWDGAGSQWFKIYHEQ 125
arthrobotrys_oligospora_11/1-1    43  FRVDTS----------------ISHPGPLLFYLAKVPSGRTA-ANWDGDGAVWFKIWENR 85
pyrenophora_trici_repentis_20/    81  FTAATS----------------ISHPGTLQFYLAKVPSGKTA-ATWDGSGTEWFKIYSQG 123
Phaeospheraria_nodorum_28/1-22    79  FTAVSS----------------VSHPGPLQFYMAKVPSGKTA-ATFDGSGAVWFKVYSQG 121
myceliophthora_thermophilia_16    81  FSVDPS----------------IGHPGPLQFYMAKVPSGQTA-ATFDGTGAVWFKIYQDG 123
thievela_terestis_11/1-231        82  FTVDPS----------------IQHPGPLQFYMAKAPSGQTA-ATFQGTGNVWFKIYEDG 124
chaetomium_globosum_24/1-226      82  FTVDTS----------------IGHPGPLHFYLAKVPSGQTA-ATFDGKGAVWFKIYEDG 124
chaetomium_thermohilum_14/1-23    82  FTVDSN----------------IGHPGPLHFYMAKVPAGQTA-ATFNGKGPVWFKIYQDG 124
TYPE1:NCU02344/1-232              82  FVVGDN----------------IGHPGPLHFYMAKVPEGKTA-ATFDGKGAVWFKIYQDG 124
myceliophthora_thermophilia_2/    81  FTTDTP----------------VYHQGPTSIYMSKAPGSAS---DYD-GSGGWFKIKDWG 120
sodaria_macrospora_4/1-217        79  FKTDTP----------------VYHQGPTSIYLSKAPGSLS---SYD-GSGGWFKIKDFG 118
neurospora_tetrasperma_12/1-21    80  FKTDTA----------------VYHQGPTSVYLSKADGSLS---SYD-GSGGWFKIKDWG 119
TYPE1:NCU00836/1-218              80  FKTDTA----------------VYHQGPTSVYLSKADGSLS---DYD-GSGGWFKIKDWG 119
thievela_terestis_7/1-223         81  FTLDTP----------------VYHQGPISIYMSKAPGAAS---DYD-GSGGWFKIKDWG 120
podospora_anseria_5/1-221         82  FYTDQA----------------VYHQGPISLYMSKAPGSVK---DYD-GSGEWFKIYDWG 121
leptosphaeria_maculans_15/1-17    18  AEWHHTLDSKPE-TDKAD-PIDPGHLGPIMVYLAKVDDALT----TKVTGLKWFKIYEDG 71
glomerrela_graminic_9/1-232       78  AIWRHTLTSGPDD------VMDASHKGPTIAYLKKVNDAKT----DTGVGGGWYKIQEDG 127
glarea_lozoyensis_6/1-240         81  AVWRHTLTSTPA-NDAIY-VIDPSHKGPTIAYLKKVTDATT----DSGVGDGWFKIQEQG 134
TYPE2:NCU02240/1-235              78  AIWRHTLTSGADD------VMDASHKGPTLAYLKKVDDALT----DTGIGGGWFKIQEDG 127
botryotinia_fuckeliana_2/1-234    75  AIWRHTLDSTPA-NDATY-VLDPSHLGPVMAYMKKVTDATT----DVGYGPGWFKISEQG 128
botryotinia_fuckeliana_12/1-24    81  AIWRHTLDSTPA-NDATY-VLDPSHLGPVMAYMKKVTDATT----DVGYGPGWFKISEQG 134
sclerotinia_sclerot_5/1-240       81  AIWRHTLTSTPS-NDATY-VLDPSHLGPVMAYMKKVDDATT----DVGYGPGWFKISEQG 134
Phaeospheraria_nodorum_9/1-227    74  LTWRHTLTSGDND------IIDPSHKGPVMAYLKKVSDAKT----DSGVGGGWFKISEDA 123
chaetomium_thermophilum_18/1-2    78  AIWRYMLNSQG---SAPNDVMDSSHKGPTMAYLKKVNNATT----DSGVGDGWFKIQEDG 130
cholletotrichum_higginsianum_3    78  AIWRYMLSTTG---SGPADVMDSSHKGPTIAYLKKVGDATT----DTGVGNGWFKIQQDG 130
podospora_anseria_30/1-234        77  AIWRYMLSTTG---SAPNDIMDISHKGPTMAYLKKVNDATT----DSGVGGGWFKIQEDG 129
myceliophthora_thermophilia_10    78  AVWRYMLSTTG---SAPNDIMDSSHKGPTMAYLKKVDNATT----DSGVGGGWFKIQEDG 130
neurospora_crassa_1/1-236         78  AIWRYMLSTTG---DSPADVMDSSHKGPTIAYLKKVDNAAT----ASGVGNGWFKIQQDG 130
TYPE2:NCU01050/1-226              78  AIWRYMLSTTG---DSPADVMDSSHKGPTIAYLKKVDNAAT----ASGVGNGWFKIQQDG 130
neurospora_tetrasperma_1/1-236    78  AIWRYMLSTTG---DSPADVMDSSHKGPTIAYLKKVDNAAT----ASGVGNGWFKIQQDG 130
sodaria_macrospora_11/1-236       78  AIWRYMLSTTG---DSPADVMDSTHKGPTIAYLKKVDNAAT----DSGVGNGWFKIQQDG 130
Paravalsa_indica_8/1-210          78  VEI----------------------------YMAKVSDAKT----AVGSSAGWFKVSEMG 105
pyrenophora_trici_repentis_11/    75  VEMHAQPNARS---CSQP-AIGGNHYGPVMVYMAKVADAKT------ATSGSFFKVAEDG 124
leptosphaeria_maculans_11/1-22    75  VEMHAQPGDRK---CTQP-AIGGNHYGPVLIYMAKVADAKT------ATSGSFFKVAEDG 124
Phaeospheraria_nodorum_14/1-22    75  VEMHAQPGDRK---CTQP-AIGGAHYGPVLVYMAKVADAKT------ATSGSFFKVAEDG 124
pyrenochaeta_lyocope_1/1-227      75  VEMHAQPGDRS---CSNP-AIGGNHYGPVLIYMAKVSDAKT------ATSGSFFKVAEDG 124
pyrenophora_terestis_25/1-227     75  VEMHSQPNARS---CSQP-AIGGAHYGPVMVYMAKVADAKT------ATSGSFFKVAEDG 124
glarea_lozoyensis_1/1-214         59  VEMHAQPGDRV---CTKE-AIGGNHFGPVMIYMSKVASAAS-----DPGSGSWFKISEEG 109
myceliophthora_thermophilum_12    77  VEMHQQPGDRS---CSSE-AIGGAHYGPVMVYMSKVSDAAS-----ADGSSGWFKVFEDG 127
chaetomium_thermophilum_7/1-24    78  IEMHAQPGDRD---CSKE-AMGGAHHGPVLVYMTKVSDAAT-----ADGSTGWFKIFEDG 128
podospora_anseria_15/1-234        73  VEMHQQTGDRS---CSQE-AIGGAHYGPVQVYLSKVPDAST----ADGTSTGWFKILSNS 124
thievela_terestis_10/1-235        73  VEMHQQPGDRS---CNNE-AIGGAHWGPVQVYLSKVEDAST-----ADGSTGWFKIFADT 123
podospora_anseria_24/1-240        78  VEMHAQNGDRS---CSQE-AIGGAHHGPVSVYLTKVSDALT-----ADGSTGWFKIFDDG 128
pyrenophora_teres_17/1-233        71  IEMHQQNGDRS---CANE-AIGGAHYGPVLVYMSKVADAST-----ADGSAPFFKIFQDT 121
pyrenophora_trici_repentis__8/    71  IEMHQQNGDRS---CSNE-AIGGAHYGPVLVYMSKVADAST-----AEGSAPFFKIFQDT 121
chaetomium_globosum_22/1-239      77  VEMHQQNGDRS---CNNE-AIGGAHYGPVIVYMSKVSDAAS-----ADGSSGWFKVFQDG 127
thievela_terestis_17/1-240        78  IEMHQQPGDRS---CANE-AIGGDHYGPVMVYMSKVDDAVT-----ADGSSGWFKVFQDS 128
glomerrela_graminic_32/1-232      78  VEMHQHNDRD----CTKE-AIGGAHHGPVMVYMSSVADAAA-----ADGSEPFFKIFESG 127
verticillium_dahliae_23/1-243     76  AWYQHVLGGEQYPGDVDN-PIAASHKGPITAWLAKVEDAAT----SSHANLEWFKIAEDG 130
```

```
verticilium_albo_atrum_15/1-21     54  AWYQHILGGEQYPGDVDN-PIAASHKGPITAWLAKVDDAAT----SSHANLEWFKIAEDG 108
glomerrela_graminic_17/1-244       77  AWWQHVIGGAQFPGDPDN-PIASSHKGPITAWLAKVPSASG----GGVTGLQWFKVAEDT 131
chaetomium_globosum_23/1-244       77  AWYQHVIGGAQFPNDPDN-PIASSHKGPVMAYLAKVDNAAS----ASLNGLKWFKIWEDT 131
podospora_anseria_29/1-244         77  AYFGHVIGGAQFPNDRDH-PIAASHKGPVQAYLAKVDNAAT----ASSNGQKWFKIWHEG 131
chaetomium_thermophilum_15/1-2     77  TFWGHVIGGAQFPGDPDN-PIARSHKGPIQVYLAKVDNAAS----ASHTGLRWFKIWSEG 131
sodaria_macrospora_16/1-245        77  AFWGHVIGGPQFPNDQDN-PIAKSHKGPVIAYLAKVNDNAAS---ASSTGQQWFKIWQDG 132
myceliophthora_thermophilia_5/     77  AWYQHVIGGAQFPNDPDN-PIAKSHKGPVMAYLAKVDNAAT----ASKTGLKWFKIWEDT 131
thievela_terestis_2/1-244          77  SLWQHVIGGAQFSGDPDN-PIAHSHKGPVMAYLAKVDNAAS----ASQTGLKWFKIWQDG 131
Paravalsa_indica_13/1-241          76  TYWQHVIGGPQGSNDPDN-PIAKSHKGPIQIYLAKVDNAAS----ASSSGQRWFKVASEG 130
Paravalsa_indica_12/1-241          76  TYWQHVIGGPQGSNDPDN-PIAKSHKGPIQVYLAKVDNAAS----ASSSGQKWFKVASEG 130
myceliophthora_thermophilia_6/     76  AWWQHVIGGPQGANDPDN-PIAASHKGPIQVYLAKVDNAAT----ASPSGLKWFKVAERG 130
chaetomium_globosum_31/1-241       76  AWWQHEIGGPSGPNDQDN-PIAASHKGPITVYLAKVSNAAS----ASASGQQWFKIAERG 130
podospora_anseria_4/1-241          76  AWWGHEIGGAAGPNDPDH-PIAASHKGPIQVYLAKVNNAAN----AGTSGLQWFKVAEQG 130
sodaria_macrospora_2/1-240         76  HWWGHEFGGASGPNDLDN-PIAASHKGPVIVYLAKVDNAAT----TGTSGLKWFKVAEAG 130
neurospora_tetrasperma_16/1-24     76  HWWGHEIGGAAGPNDADN-PIAASHKGPIIVYLAKVDNAAT----TGTSGLKWFKVAEAG 130
neurospora_tetrasperma_14/1-24     76  HWWGHEIGGASGPNDADN-PIAASHKGPIIVYLTKVDNAAT----TGTSGLKWFKVAEAG 130
TYPE2:NCU02916/1-241               76  HFWGHEIGGAAGPNDADN-PIAASHKGPIMVYLAKVDNAAT----TGTSGLKWFKVAEAG 130
4EIR/1-223                         63  AIWRYMLSTTG---DSPADVMDSSHKGPTIAYLKKVDNAAT----ASGVGNGWFKIQQDG 115
pyrenophora_trici_repentis_22/     61  FEIRSWPNNP-----SKE-RLDRGHKGPCAVYLKKVNNAAT----DTAAGDGWFKIFDHG 110
verticillium_dahiae_24/1-222       80  FPSSAP----------------IGHPGPVLFYLARVPDGQDV-ISWTPTGNVWFKVDQYG 122
verticilium_albo_atrum_16/1-21     75  FPSSVP----------------IGHPGPVLFYLARVPDGQDV-NSWTPTGDVWFKVDQYG 117
verticillium_dahliae_26/1-221      80  FTSSNS----------------MGHPGPVLFYMARVPDGEDV-TTWDPVGDVWFKIDQHG 122
3ZUD/1-228                         77  LQWTPW---------------PDSHHGPVINYLAPCNGDCS---TVDKTQLEFFKIAESG 118
TYPE3:NCU07898/1-239               89  LRWTIW---------------PDSHVGPVITYMARCPDTGCQDWTPSASDKVWFKIKEGG 133
2YET/1-228                         77  LQWTPW---------------PDSHHGPVINYLAPCNGDCS---TVDKTQLEFFKIAESG 118
thermoascus_auranti_1/1-228        77  LQWTPW---------------PDSHHGPVINYLAPCNGDCS---TVDKTQLEFFKIAESG 118
4EIS/1-224                         74  LRWTIW---------------PDSHVGPVITYMARCPDTGCQDWTPSASDKVWFKIKEGG 118
giberella_zeae_7/1-251            103  LRWSGW---------------PENHSGPILNYLANCNGPCE---RVDKTKLEFFKIDGLG 144
fusarium_oxysporum_3/1-252        104  LRWSGW---------------PENHIGPVLNYLANCKGPCE---RVDKTELEFFKIDGLG 145
nectria_heamatococcuss_1/1-252    104  IKWSSW---------------PENHRGPVMDYLANCNGPCE---TVDKTKLEFFKIDGMG 145
verticilium_albo_atrum_13/1-24     95  LKWSPW---------------PESHKGPIIDYLANCNGDCT---TVDKTALRFFKIAEQG 136
verticillium_dahliae_4/1-241       95  LKWSPW---------------PESHKGPIIDYLANCNGDCT---TVDKTALRFFKIAEQG 136
neurospora_tetrasperma__2/1-24     95  LQWDQW---------------PESHKGPVIDYLAACDGDCE---SVDKTALKFFKIDGAG 136
TYPE3:NCU07760/1-240               95  LQWDQW---------------PESHKGPVIDYLAACDGDCE---SVDKTALKFFKIDGAG 136
magna_porte_oryzae_16/1-243        95  LQWDTW---------------PESHHGPVIDYLARCSGNCE---TVDKTSLRFFKIGAGT 136
podospora_anseria_31/1-244         97  IVWTPEW--------------PESHIGPVIDYLANCNGPCE---TVDKTSLRWFKIGGAG 139
hypocrea_orientalis_1/1-246        98  FQWVPV---------------PWPHPGPIVDYLANCNGDCE---TVDKTSLEFFKIDGVG 139
trichoderma_SP_SSL_1/1-246         98  LQWVPV---------------PWPHPGPIVDYLANCNGDCE---TVDKTSLEFFKIDGVG 139
Hypocrea_virens_3/1-246            98  IQWVPI---------------PWPHPGPVLDYLANCNGDCE---TVDKTTLEFFKIDGIG 139
trichoderma_atroviride_2/1-246     98  FQWVPV---------------PWPHKSTVVDYLANCNGPCE---TVDKTTLEFFKIDGIG 139
hypocrea_rufa_1/1-246              98  FQWVPV---------------PWPHPGPIVDYLANCNGDCE---TVDKTTLEFFKIDGVG 139
hypocrea_rufa_2/1-246              98  FQWVPV---------------PWPHPGPIVDYLANCNGDCE---TVDKTTLEFFKIDGVG 139
trichoderma_saturnusporum_1/1-     98  FQWVPL---------------PWPHPGPIVDYLANCNGDCE---TVDKTSLEFFKIDGVG 139
aspergillus_kawachii_37/1-247      98  IQWTAW---------------PDSHHGPVLDYLARCDGSCE---TVDKTDLEFFKIDGVG 139
aspergillus_tereus_6/1-245         96  LQWTEW---------------PESHHGPVLDYLAPCGTAGCE--TVDKTTLQFFKIDGVG 138
neosartorya_fischeri_4/1-247       98  IQWTTW---------------PDSHHGPVIDYLARCGSSCE---TVDKTTLEFFKIDGVG 139
aspergillus_fumingatus_4/1-247     98  IQWTAW---------------PDSHHGPVIDYLARCGSSCE---TVDKTTLEFFKIDGVG 139
aspergillus_tereus_10/1-245        97  LQWNTW---------------PESHHGPVLDYLADCGGSCE---TVDKTTLKFFKIDGVG 138
chaetomium_globosum_5/1-220        98  IQWDTW---------------PESHHGPVIDYLASCGDAGCE--TVDKTSLEFFKVAEAG 140
TYPE3:NCU05969/1-243               97  IQWETW---------------PESHKGPVIDYLANCGASGCE--TVDKTSLEFFKIDEVG 139
2VTC/1-228                         80  FQWGPGV--------------WPHPYGPIVTYVVECSGSCT---TVNKNNLRWVKIQEAG 122
aspergillus_fumingatus_1/1-245     96  FQWTEW---------------PESHHGPVITYMANCNGDCA---SVDKTTLEFFKIDESG 137
neosartorya_fischeri_1/1-245       96  FQWTEW---------------PESHHGPVITYMANCNGDCA---SVDKTSLEFFKIDESG 137
aspergillus_niger_1/1-245          96  MTWNTW---------------PDDHHGPVITYLANCNGSCS---DVDKTALQFFKIDAGG 137
aspergillus_kawachii_40/1-245      96  MTWNTW---------------PDDHHGPVITYLANCNGSCA---DVDKTALQFFKIDAGG 137
aspergillus_tereus_5/1-232         96  MQWNTW---------------PESHHGPVLNYLAPCNGDCA---QADKSSLEFFKIEAEG 137
emmericella_nidulans_9/1-245       96  LQWTEW---------------PESHHGPVIDYIASCNGDCT---TVDKTTLEWVKISESG 137
penicillum_chrysogenum_2/1-245     96  LQWTEW---------------PESHHGPVINYLANCNGDCS---KVDKKTLKWFKIAESG 137
aspergillus_niger_2/1-245          96  LQWTDW---------------PSSHHGPVLTYLANCNGDCS---DVTKTDLEFFKIDESG 137
aspergillus_niger_12/1-244         96  LQWTDW---------------PSSHHGPVLTYLANCNGDCS---DVTKTDLEFFKIDESG 137
aspergillus_kawachii_38/1-245      96  LQWTDW---------------PSSHHGPVLTYLANCNGDCS---DVTKTDLEFFKIDESG 137
zea_mys_1/1-245                    96  LQWTDW---------------PSSHHGPVLTYLANCNGDCS---DVTKTDLEFFKIDESG 137
aspergillus_clavatus_6/1-241       96  FQWTQW---------------PESHHGPVITYMANCNGDCA---SVDKSQLEFFKIEEKG 137
aspergillus_tereus_8/1-241         96  LQWTEW---------------PESHHGPVITYLANCNGDCS---SVDKTSLEFFKIDQKG 137
aspergilus_oryzae_7/1-242          95  LQWTAW---------------PESHKGPVITYLANCNGDCA---TVDKTQLEFFKIDEKG 136
aspergillus_favus_5/1-242          95  LQWTAW---------------PESHKGPVITYLANCNGDCA---TVDKTQLEFFKIDEKG 136
glomerrela_graminic_4/1-242        98  VFWDTW---------------PQSHVGPIIDYLAPCGDDCS---TVDKSSLEFFKIQEAG 139
aspergillus_fumingatus_3/1-238     91  MKLAVGA--------------TMQHPGPALVYMSKAPSSAK---TYE-GDGDWFKIHEEG 132
cholletotrichum_higginsianum_2     91  FKLGVGA--------------TMQHPGPAIVYMSKAPSTAK---AYE-GDGDWFKVYEES 132
glomerrela_graminic_7/1-238        91  FKLGVGA--------------TMQHPGPGLAYMSKAPTTAA---AYE-GDGDWFKIYEES 132
aspergillus_clavatus_5/1-238       91  LKLAVNA--------------HIQHPGPSLVYMSKAPNGVK---NYQ-GDGDWFKIFEDS 132
aspergilus_oryzae_5/1-238          91  MKLGVGA--------------TMKHPGPALVYMSKAPSTAK---TYQ-GDGDWFKIYEEG 132
aspergillus_favus_3/1-238          91  MKLGVGA--------------TMKHPGPALVYMSKAPSTAK---TYQ-GDGDWFKIYEEG 132
aspergillus_niger_9/1-238          91  MKLGVGA--------------TMKHPGPGLVYMSKAPGSAK---QYE-GDGDWFKIHEEG 132
emmericella_nidulans__6/1-238      91  MKLGVGA--------------TMQHPGPGLVYMSKAPGAAN---QYE-GDGDWFKIHEEG 132
aspergillus_clavatus_1/1-238       91  MKLAVGA--------------TFQHPGPGLVYMSKAPSAAK---AYQ-GDNEWFKIHEES 132
neosartorya_fischeri_7/1-238       91  LKLAVGA--------------TMQHPGPALVYMSKAPSSAK---SYE-GDGDWFKIHEEG 132
```

```
penicillium_chrysogenum_4/1-236   89  MKLAVGA--------------TMQHPGPALVYMSKAPTTAK---EYE-GDGEWFKIHQES 130
verticilium_albo_atrum_17/1-21    91  VKLAVGA--------------TMQHPGPALVYMSKAPATAK---AYE-GNGEWFKIFEES 132
verticillium_albo_atrum_17/1-2    91  VKLVVGA--------------TMQHPGPALVYMSKAPTTAK---SYE-GDGEWFKIFEES 132
pyrenophora_teres_22/1-230        83  FKLSFNE--------------KIEHPGCGFVYVSKAPSKVN---TYD-GSGEWTKVMQSC 124
Phaeospheraria_nodorum_4/1-230    83  FKLAFGE--------------KIEHPGPGFVYIAKAPGAVK---SYD-GSGDWVKVMESG 124
pyrenophora_trici_repentis_23/    67  FKLAYDE--------------LIEHPGCGFVYISKAPGKVN---NYD-GSGEWTKVMQSC 108
podospora_anseria_17/1-231        84  FKVFNNE--------------FIEHPGPGFIYMSKAPGSVA---TYD-GSGEWFKVYETG 125
glomerrela_graminic_16/1-230      83  FKLWYNE--------------FIEHPGPGFVYMSKVSGSLN---GYD-GSGDWFKVYETG 124
glomerrela_graminic_31/1-230      83  FKLWFNE--------------FIEHPGPGMVYMSKAPGDLN---SYD-GSGDWFKVFESG 124
chaetomium_globosum_15/1-229      82  FKLFNNE--------------FIEHPGPGFVYMSLAPNGVA---NYD-GSGDWFKVYETG 123
neosartorya_fischeri_2/1-235      86  FQLAYGA--------------EMKHPGPLQIYMSKAPGDVK---SYD-GSGDWFKVHQEG 127
aspergillus_tereus_12/1-235       86  FGLAYGA--------------SMKHPGPLQVYMSKAPGDVK---EYD-GSGDWFKVYQEG 127
aspergillus_favus_7/1-235         86  FQLAYGA--------------SMKHPGPLQIYMSKAPGDVK---TYD-GSGDWFKVYQEG 127
aspergillus_fumingatus_6/1-235    86  FQLAYGA--------------EMKHPGPLQIYMSKAPGDVR---SYD-GSGDWFKVHQEG 127
4B5Q/1-217                        68  FKANSA----------------IYHPGYLDVMMSPASPAANS--PEAGTGQTWFKIYEEK 109
3EJA/1-208                        60  YWANPD----------------VYHPGPVQFYMARVPDGEDI-NSWNGDGAVWFKVYEDH 102
3EII/1-208                        60  YWANPD----------------VYHPGPVQFYMARVPDGEDI-NSWNGDGAVWFKVYEDH 102

                                                190       200       210       220       230       240
                                      ....|....|....|....|....|....|....|....|....|....|....|....|
serpula_lacrymans_5/1-229         124 AITN--------GGTSISWPSVGMSQVTFTLPASIPDGQYFVRAEDIALHVAESYGGAQF 175
schizzophylum_commune_15/1-228    123 AVTD--------GGSTIEFPGTNITEITFDIPKALPSGEYLVRAEHIALHNAANKGGAQF 174
schizzophylum_commune_16/1-228    123 PVTN--------GGSSISFPGSDIDTITFTIPKSLPSGDYLIRAEHIALHSASSFGGAQF 174
chaetomium_globosum_8/1-223       120 PNFG----------SQLTWPSNGKSSFDVSIPSCIKAGKYLLRAEHIGLHVAQSSGGAQF 169
pyrenophora_trici_repentis_13/    119 PKFG----------SQLTWLS--AANYNINIPSCIAPGKYLMRNEHIAIHTAGTRGGAQF 166
pyrenophora_teres_11/1-209        119 PKFG----------SQLTWLS--AASYNINIPSCIAPGKYLMRNEHIALHTAGSRGGAQF 166
Phaeospheraria_nodorum_18/1-21    117 PKFG----------SQLTWLA--AANYDIKIPSCIAPGKYLMRNEHIAIHNAGTKGGAQF 164
podospora_anseria_18/1-223        120 PTFG----------QQLGWPSLNKGSFPVTIPRCIRSGYYLLRAEHIALHSASSPGGAQF 169
thievela_terestis_18/1-224        121 PTFG----------AQLTWPSTGKSSFAVPIPPCIKSGYYLLRAEQIGLHVAQSVGGAQF 170
myceliophthora_thermophilia_21    120 PQFG----------AQLTWPSNGKSSFEVPIPSCIRAGNYLLRAEHIALHVAQSQGGAQF 169
TYPE1:NCU03328/1-229              124 PTFG----------SQLTWSSNGKSSFPVKIPSCIKSGSYLLRAEHIGLHVAQSSGAAQF 173
emmericella_nidulans__3/1-229     125 PTVT---------DDGLTWPSDGATNVNFTIPSSLPDGDYLLRVEHIALHGAGTEGGAQF 175
aspergillus_tereus_4/1-228        124 PKFN---------ADGLTWPTEGATQVTFDLPAALPDGDYLVRVEHIGLHSANTEGGAQF 174
podospora_anseria_11/1-231        125 PSGL--------GTGSIKWPSDGKTEVSVQIPSCIANGEYLLRVEHIALHSAGSVGGAQL 176
glomerrela_graminic__6/1-230      126 PKV---------TSNGLEWASLNALVLSAKIPSCLASGEYLVRVEHLALHAAGDTGGAQF 176
arthrobotrys_oligospora_11/1-1    86  PTI---------TSSSISWPS-GQTQVFVTIPSCIPAGEYLLRVEHIALHSAGSAGGAQL 135
pyrenophora_trici_repentis_20/    124 PSF---------SGGQLTWPSNGKTEVDVTLPKSLPSGEYLLRGEHIALHSAGSAGGAQF 174
Phaeospheraria_nodorum_28/1-22    122 ATF---------SGGQMTFASAGKTQVTFPLPKSLPSGEYLLRVEHIALHSAGTSGGAQF 172
myceliophthora_thermophilia_16    124 PNGL--------GTDSITWPSAGKTEVSVTIPSCIEDGEYLLRVEHIALHSASSVGGAQF 175
thievela_terestis_11/1-231        125 PSGL--------GTSNITWPSSGKTEVSVKIPSCIAPGDYLLRVEHIALHSASTVGGAQF 176
chaetomium_globosum_24/1-226      125 PSGL--------GTGNLKWPSDGKTQVSVKIPSCVQNGD-----TPPPKHLTNILPLTEL 171
chaetomium_thermohilum_14/1-23    125 PGGL--------GTSSLTWPSYGKTEVSVQIPHCIQDGDYLLRVEHIALHSASSIGGAQL 176
TYPE1:NCU02344/1-232              125 PMGL--------GTGQLTWPSAGATEVSVKLPSCLESGEYLLRVEHIGLHSAGSVGGAQL 176
myceliophthora_thermophilia_2/    121 ADFS-----------SGQATWTLASDYTATIPECIPPGDYLLRIQQLGIHNPWPAGIPQF 169
sodaria_macrospora_4/1-217        119 ATFP-------------GGGWTLSDTYTFTIPSCIPSGDYLLRIQQIGIHNPWPAGIPQF 165
neurospora_tetrasperma_12/1-21    120 ATFP-------------GGEWTLSDTYTFTIPSCIPSGDYLLRIQQIGIHNPWPAGVPQF 166
TYPE1:NCU00836/1-218              120 ATFP-------------GGEWTLSDTYTFTIPSCIPSGDYLLRIQQIGIHNPWPAGVPQF 166
thievela_terestis_7/1-223         121 PTFN----------ADGTATWDMAGSYTYNIPTCIPDGDYLLRIQSLAIHNPWPAGIPQF 170
podospora_anseria_5/1-221         122 PTIN-----------GGQSSWPMRSSYQANLPRCIPNGEYLLRIQSLAIHNPG--STPQF 168
leptosphaeria_maculans_15/1-17    72  MDAN--------GEWAVTRLYNKKGLVDFAIPSCIPSGHYLLRAEIIALHGAGSYPGAQF 123
glomerrela_graminic_9/1-232       128 FSN---------GVWGTERVINNAGKHNITIPKCIANGQYLLRAEMIALHSASSYPGAQL 178
glarea_lozoyensis_6/1-240         135 YNPS-------TGSWGVTDLIANAGLHSITIPSCLANGQYLLRAELIALHSAGSSQGAQL 187
TYPE2:NCU02240/1-235              128 YNN---------GQWGTSTVITNGGFQYIDIPACIPSGQYLLRAEMIALHAASSTAGAQL 178
botryotinia_fuckeliana_2/1-234    129 LNVA-------TQGWATTDLINNAGVQSITIPSCIANGQYLLRAELIALHSAGGSPGAQL 181
botryotinia_fuckeliana_12/1-24    135 LNVA-------TQGWATTDLINNAGVQSITIPSCIANGQYLLRAELIALHSAGGSQGAQL 187
sclerotinia_sclerot_5/1-240       135 LNVA-------TQGWATTDLINNAGVQSITIPSCIANGQYLLRAELIALHAASGLQGAQL 187
Phaeospheraria_nodorum_9/1-227    124 FDG---------SKWGVDRLIANKGVQTVKIPACIAPGQYLLRGELIALHSASSSMGAQF 174
chaetomium_thermophilum_18/1-2    131 FDG---------TTWGTERVINGQGRHKIKIPECIEPGQYLLRAEMLALHGASNYPGAQF 181
cholletotrichum_higginsianum_3    131 FSN---------GVWGTEKVINGQGRHSIKIPECIAPGQYLLRAEMIALHGAGSYPGAQF 181
podospora_anseria_30/1-234        130 YNN---------GVWGTEKVINGQGRHSIKIPSCIAPGQYLLRAEMLALHGAGNYPGAQF 180
myceliophthora_thermophilia_10    131 LTN---------GVWGTERVINGQGRHNIKIPECIAPGQYLLRAEMLALHGASNYPGAQF 181
neurospora_crassa_1/1-236         131 MDSS--------GVWGTERVINGKGRHSIKIPECIAPGQYLLRAEMIALHAASNYPGAQF 182
TYPE2:NCU01050/1-226              131 MDSS--------GVWGTERVINGKGRHSIKIPECIAPGQYLLRAEMIALHAASNYPGAQF 182
neurospora_tetrasperma_1/1-236    131 MDSS--------GVWGTERVINGKGRHSIKIPECIAPGQYLLRAEMIALHAASNYPGAQF 182
sodaria_macrospora_11/1-236       131 MDAN--------GVWGTERVINGKGRQSIKIPECIAPGQYLLRAEMIALHSAGNYPGAQF 182
Paravalsa_indica_8/1-210          106 LASNN------PNYYGSQVLNDNCGHYTFTVPNV-APGNYLLRAEVIALHVASSVGGAQF 158
pyrenophora_trici_repentis_11/    125 YKGTT-------ATWGTEILNANCGKRAFTVPKNIASGDYLVRAEVIALHAG--AGQSQP 175
leptosphaeria_maculans_11/1-22    125 YTGTT-------ASWGTEILNANCGKRAFTVPKNIASGDYLVRAEAIALHAG--AGNPQP 175
Phaeospheraria_nodorum_14/1-22    125 YSGTT-------ASWGTEILNANCGKRAFTVPKNIASGDYLVRAEALALHAG--VGQPQP 175
pyrenochaeta_lyocope_1/1-227      125 HAGTV-------ATWGTEILNANCGKRAFTVPKSLASGDYLVRAEVLALHAG--AGNAQP 175
pyrenophora_terestis_25/1-227     125 YKGTT-------ATWGTEILNANCGKRAFTVPKNIASGDYLVRAEAIALHAG--ANQPQP 175
glarea_lozoyensis_1/1-214         110 YNPST-------KKWGTDSLNANCGKRTATIPSSLAPGDYLVRAEAIALHSASSAGGAQF 162
myceliophthora_thermophilum_12    128 WAKNPSGGSGDDDYWGTKDLNSCCGKMNVKIPADLPSGDYLLRAEALALHTAGSAGGAQF 187
chaetomium_thermophilum_7/1-24    129 WAKNPSGKVGDDDFWGTKDLNTCCGKMDVKIPEDIPSGDYLLRAEAIALHVGGQPGGAQL 188
podospora_anseria_15/1-234        125 WSKKAGGRVGDDDNWGSKDLNACCGKMDVKIPADIPSGDYLLRAESLALHAAGPSGGGQF 184
thievela_terestis_10/1-235        124 WSKKAGSSVGDDDNWGTRDLNACCGKMQVKIPADIPSGDYLLRAEALALHTAGQVGGAQF 183
podospora_anseria_24/1-240        129 WRKNPSGRVGDDDFWGTKDLNACCGKMNVKIPSDIPSGDYLLRAEAIALHAAGGAGGAQP 188
```

```
pyrenophora_teres_17/1-233          122 WAKNTGGGGGSDDYWGTKDLNKNCGKMDVKIPANLASGDYLLRAEAIALHASSGLGGAQF 181
pyrenophora_trici_repentis__8/      122 WAKNTGGGGGSDDYWGTKDLNKNCGKMDVKIPANLASGDYLLRAEAIALHAASGLGGAQF 181
chaetomium_globosum_22/1-239        128 WAKKSGGGSGDDDYWGVKDLNACCGKMDVKIPSDIAPGDYLLRAEVIALHTAGGAGGAQL 187
thievela_terestis_17/1-240          129 WAKNPSGSTGDDDYWGTKDLNSCCGKMNVKIPEDIEPGDYLLRAEVIALHVAASSGGAQF 188
glomerrela_graminic_32/1-232        128 YFANN-------NTWGNDLINEGCGKQNVVIPADIAPGDYLLRAETVALHAAGSVDGAQY 180
verticillium_dahliae_23/1-243       131 FDNTR-------GIWGVDNLLQQDGWTYFGIPECIAPGDYILRVELLALHSAYVPRGSQF 183
verticilium_albo_atrum_15/1-21      109 FDNTR-------GIWGVDNLLQQDGWTYFDVPECIAPGDYLLRVELLALHSAYVPKGSQF 161
glomerrela_graminic_17/1-244        132 LDTGS-------GQWAVDRMIGGGGWSYFDLPRCIAPGDYLLRVELLALHSAYSQKGAQF 184
chaetomium_globosum_23/1-244        132 FDTGS-------RVWGVDNLLKNNGWVYFNLPQCVASGQYLLRVEVLALHSAGKQGQAQF 184
podospora_anseria_29/1-244          132 FDQGS-------RKWGVDTVIQNQGWTYFNIPQCIAPGQYLLRVELLALHSANQQGQAQF 184
chaetomium_thermophilum_15/1-2      132 FDVGS-------KRWGVDTMINNGGWYYFNLPQCIADGQYLMRVELLALHSAGNYRGAQF 184
sodaria_macrospora_16/1-245         133 FETSS-------RKWGVDNMLQNNGWTYFNLPNCIAPGQYLLRVEIIALHSAKNSGQAQF 185
myceliophthora_thermophilia_5/      132 FNPST-------KTWGVDNLINNNGWVYFNLPQCIADGNYLLRVEVLALHSAYSQGQAQF 184
thievela_terestis_2/1-244           132 FDTSS-------KTWGVDNLIKNNGWVYFHLPQCLAPGQYLLRVEVLALHSAYQQGQAQF 184
Paravalsa_indica_13/1-241           131 LSGG---------RWAVDTMIANNGMWSFKLPSCIAPGQYLMRGELLALHSAGSVNGEQF 181
Paravalsa_indica_12/1-241           131 LNGG---------KWAVDTMIANNGMWSFKLPSCIAPGQYLLRGELLALHSAYSQNGEQF 181
myceliophthora_thermophilia_6/      131 LNNG---------VWAVDELIANNGWHYFDLPSCVAPGQYLMRVELLALHSASSPGGAQF 181
chaetomium_globosum_31/1-241        131 VNNG---------VWAVDEMISNNGWHYFDVPSCVAPGQYLMRVELLALHSASTAGGAQF 181
podospora_anseria_4/1-241           131 LNNG---------VWAVDNMISNGGWHYFDMPSCVAPGHYLMRVELLALHSASVRGAAQF 181
sodaria_macrospora_2/1-240          131 FSGG---------KWAVDDLIANNGWSYFDMPTCIAPGQYLMRAEIIALHNAGSSQGAQF 181
neurospora_tetrasperma_16/1-24      131 LSNG---------KWAVDDLIANDGWSYFNMPTCIAPGQYLMRAELIALHNAGSQAGAQF 181
neurospora_tetrasperma_14/1-24      131 LSNG---------KWAVDDLIANDGWSYFNMPTCIAPGQYLMRAEIIALHNAGSQAGAQF 181
TYPE2:NCU02916/1-241                131 LSNG---------KWAVDDLIANNGWSYFDMPTCIAPGQYLMRAELIALHNAGSQAGAQF 181
4EIR/1-223                          116 MDSS--------GVWGTERVINGKGRHSIKIPECIAPGQYLLRAEMIALHAASNYPGAQF 167
pyrenophora_trici_repentis_22/      111 YNSAT-------DRWCTDEIIDNNGLLSVNLPKGLKGGDYLARPEILALHAAK-DGDPQE 162
verticillium_dahiae_24/1-222        123 NTP-----------GMNSQFAVEMTEISTIIPASLRPGNYLLRAEHIALHSY---QKPQF 168
verticilium_albo_atrum_16/1-21      118 NTP-----------GVNNQFAVEMTEVFTTIPASLRPGNYLLRAEHIALHSY---QKPQF 163
verticillium_dahliae_26/1-221       123 DLG-----------GPYPAFETEMREISTTIPKTLPNGDYLLRAEHIGLHAY---GTPQF 168
3ZUD/1-228                          119 LINDDN----PPGIWASDNLIAANNSWTVTIPTTIAPGNYVLRHEIIALHSAQNQDGAQN 174
TYPE3:NCU07898/1-239                134 REGTS-------NVWAATPLMTAPANYEYAIPSCLKPGYYLVRHEIIALHSAYSYPGAQF 186
2YET/1-228                          119 LINDDN----PPGIWASDNLIAANNSWTVTIPTTIAPGNYVLRHEIIALHSAQNQDGAQN 174
thermoascus_auranti_1/1-228         119 LINDDN----PPGIWASDNLIAANNSWTVTIPTTIAPGNYVLRHEIIALHSAQNQDGAQN 174
4EIS/1-224                          119 REGTS-------NVWAATPLMTAPANYEYAIPSCLKPGYYLVRHEIIALHSAYSYPGAQF 171
giberella_zeae_7/1-251              145 LLEQGT-----PGRYADKVLQDNGDRWNVRIPKNIAPGNYVLRHEIIALHNALDKGGAQN 199
fusarium_oxysporum_3/1-252          146 QLEQGT-----PGRYADSVLQENGDRWNVRIPENIASGNYVLRHEIIALHNALERGGAQN 200
nectria_heamatococcuss_1/1-252      146 LISQDR-----PGKYADGALRENGYTWSVRIPSNIAPGNYVLRHEIIALHSGLERNGAQN 200
verticilium_albo_atrum_13/1-24      137 LLDAA------NSNWAADELIAAGEVWEVTIPTDIAAGSYVLRHEIIALHSGGQPNGAQF 190
verticillium_dahliae_4/1-241        137 LLDAA------SSNWAADELIAAGEVWEVTIPTDIAAGSYVLRHEIIALHSGGQPNGAQF 190
neurospora_tetrasperma__2/1-24      137 YDATN--------GWASDVLIKDGNSWVVEIPENIKPGNYVLRHEIIALHSAGQANGAQN 188
TYPE3:NCU07760/1-240                137 YDATN--------GWASDTLIKDGNSWVVEIPESIKPGNYVLRHEIIALHSAGQANGAQN 188
magna_porte_oryzae_16/1-243         137 YISGSP-----PGYWASDELIKNGFSWVVQIPASIAPGNYVLRHEIIALHGAPNANGAQA 191
podospora_anseria_31/1-244          140 YNPNT-------RTWAADDLRANGNSWLVQIPADLKAGNYVLRHEIIALHGGSSPNGAQA 192
hypocrea_orientalis_1/1-246         140 LISGGD-----PGNWASDVLIANNNTWVVKIPEDLAPGNYVLRHEIIALHSAGQADGAQN 194
trichoderma_SP_SSL_1/1-246          140 LISGGD-----PGNWASDVLIANNNTWVVKIPEDLAPGNYVLRHEIIALHSAGQADGAQN 194
Hypocrea_virens_3/1-246             140 LISGGN-----PGRWASDVLIGNNGTWVVQIPADLETGNYVLRHELIALHSAGSVDGAQN 194
trichoderma_atroviride_2/1-246      140 LLSGGN-----PGTWGSDVLIGNNNTWVIQIPEDLQTGNYVLRHELIALHSAEQADGAQN 194
hypocrea_rufa_1/1-246               140 LLSGGD-----PGTWASDVLISNNNTWVVKIPDNLAPGNYVLRHEIIALHSAGQANGAQN 194
hypocrea_rufa_2/1-246               140 LLSGGD-----PGTWASDVLISNNNTWVVKIPDNLAPGNYVLRHEIIALHSAGQANGAQN 194
trichoderma_saturnusporum_1/1-      140 LISGGD-----PGNWASDVLIANNNTWVVKIPDDLAPGNYVLRHEIIALHSAGQANGAQN 194
aspergillus_kawachii_37/1-247       140 LVSDSE----VPGTWGTDQLINNNNSWMVEIPPSIAAGNYVLRHELIALHGAEEEDGAQN 195
aspergillus_tereus_6/1-245          139 LVDDAA----PPGVWAADQLIANNNSWLVQIPADIAPGDYVLRHEMIALHGAFAEDGAQN 194
neosartorya_fischeri_4/1-247        140 LVDGST----PPGVWGDDQLIADNNSWLVEIPPTIAPGYYVLRHELIALHGAGSQNGAQN 195
aspergillus_fumingatus_4/1-247      140 LVDGSN----PPGVWGDDQLIADNNSWLVEIPPTIAPGYYVLRHELIALHGAGSQNGAQN 195
aspergillus_tereus_10/1-245         139 LVDDTT----PPGIWGDDQLIANNNTWLVEIPSSIAPGNYVLRHELIALHGAGSANGAQN 194
chaetomium_globosum_5/1-220         141 LIDGTN----APGFWASDQLIANNNSWMVKIPEGIAPGNYVLRHEIIALHSGGQPNGAQN 196
TYPE3:NCU05969/1-243                140 LVDGQK--------WGSDQLIANNNSWLVEIPPTIAPGFYVLRHEIIALHSAGQPNGAQN 191
2VTC/1-228                          123 INYNT-------QVWAQQDLINQGNKWTVKIPSSLRPGNYVFRHELLAAHGASSANGMQN 175
aspergillus_fumingatus_1/1-245      138 LISDSN----VPGTWASDNLIANNNSWTVTVPSSIAAGNYVMRHEIIALHSAGNQNGAQN 193
neosartorya_fischeri_1/1-245        138 LISDSN----VPGTWASDNLIANNNSWTVTVPSSIAAGNYVMRHEIIALHSAGNQNGAQN 193
aspergillus_niger_1/1-245           138 LIDDSE----IPGTWATDKLIDNNYTRSITIPSDIEAGNYVLRHEIIALHGAEDLDGAQN 193
aspergillus_kawachii_40/1-245       138 LIDDSE----IPGTWATDKLIDNNYTRSITIPSDIEAGSYVLRHEIIALHGAEDLDGAQN 193
aspergillus_tereus_5/1-232          138 LIDGSS----PPGQWATDELISNNNTAVVTIPASIASGNYVLRHEIIGLHSAGNLNGAQN 193
emmericella_nidulans_9/1-245        138 LVDGSS----APGTWASDNLISNNNSWTVTIPSSLAAGGYVLRHEIIALHSAGNENGAQN 193
penicillum_chrysogenum_2/1-245      138 LIDGSN----APGKWASDELIANNNSASVTIPSSIAAGNYVLRHEIIALHSAGQENGAQN 193
aspergillus_niger_2/1-245           138 LISDTE----VPGTWATDNLISNNNSWTVTIPSTLEAGNYVLRHEIIALHSAENKDGAQN 193
aspergillus_niger_12/1-244          138 LISDTE----VPGTWATDNLISNNNSWTVTIPSTLEAGNYVLRHEIIALHSAENKDGAQN 193
aspergillus_kawachii_38/1-245       138 LISDTE----VPGTWATDNLISNNNSWTVTIPSTLESGNYVLRHEIIALHSAGNKDGAQN 193
zea_mys_1/1-245                     138 LISDTE----VPGTWATDNLISNNNSWTVTIPSTLEAGNYVLRHEIIALHSAENKDGAQN 193
aspergillus_clavatus_6/1-241        138 LIGS--------GHWASDDLIANNNSWTVTVPSSLAAGNYVMRHEIIGLHSAGNANGAQN 189
aspergillus_tereus_8/1-241          138 LIEGN--------TWASDQLISNNNSWTVTIPSSIASGNYVLRHEIIGLHSAGNKDGAQN 189
aspergilus_oryzae_7/1-242           137 LISGSD------NTWASDNLISSNNSWTVTIPSSIAAGNYVMRHEIIALHSAGNKDGAQN 190
aspergillus_favus_5/1-242           137 LISGSD------NTWATDNLIASNNSWTVTIPSSIAAGNYVMRHEIIGLHSAGQKDGAQN 190
glomerrela_graminic_4/1-242         140 LNSG---------KWPTDELIANNNSWAVTVPTSLKPGKYVWRHEIIALHSAGQEDGAQN 190
aspergillus_fumingatus_3/1-238      133 VCNKN------ADFTKDAWCTWDKDRIEFTIPADLPDGEYLIRPEHIGLHGAH-DGQAEF 185
cholletotrichum_higginsianum_2      133 VCNKG------GDFKGNAWCTWDKNTVEFTVPKDTPNGEYLIRVEHIGVHGAH-VGQAEF 185
glomerrela_graminic_7/1-238         133 VCNKN------GAFTSDAWCTWDKNSIEFTIPKDTPDGEYLLRIEHIGVHGAH-VGQAEF 185
aspergillus_clavatus_5/1-238        133 VCNKN------ADFTREAWCSYNKDRVEFTIPADTPDGEYLIRPEHIGVHGAH-AGQAEF 185
```

```
aspergilus_oryzae_5/1-238        133 ICDKN------KDVKSDAWCSYDKDRVEFTIPKDLADGEYLIRAEHIGVHGAH-AGEAEF 185
aspergillus_favus_3/1-238        133 ICDKN------KDVKSDAWCSYDKDRVEFTIPKDLADGEYLIRAEHIGVHGAH-AGEAEF 185
aspergillus_niger_9/1-238        133 ICDQS------KDIKTDAWCTWDKDRIEFTVPADLPDGEYLIRPEHIGVHGAH-DGQAEF 185
emmericella_nidulans__6/1-238    133 ICDTS------KDIKTDAWCTWDKDRIEFTIPADLPDGEYLIRSEHIGVHGAH-DGQAEF 185
aspergillus_clavatus_1/1-238     133 VCNKN------ADFTRDAWCTWGKDRIEFTIPADLPDGEYLIRPEHIGVHGAH-VGQAEF 185
neosartorya_fischeri_7/1-238     133 VCNKN------ADFTKNAWCTWDKDRIEFTIPSDLPDGEYLIRPEHIGLHGAH-DGQAEF 185
penicillum_chrysogenum_4/1-236   131 VCDKN------KDFTKDAWCTWDKDRIEFTIPASLPDGEYLIRPEHIGVHGAA-GGEAEF 183
verticilium_albo_atrum_17/1-21   132 ---------------------------------DTPDGEYLIRPEHIGVHGAH-VGQAEF 158
verticillium_albo_atrum_17/1-2   132 ---------------------------------DTPDGEYLFRPEHIGVHGSH-AGQAEF 158
pyrenophora_teres_22/1-230       125 LKNPS------TPGVDTAWENWQQDRLEWTIQKNIPAGEYLVRVEHIAIHEGH-VGKAQF 177
Phaeospheraria_nodorum_4/1-230   125 LKNPS------TPGVDTAWDSWQKDRLEWKIQNSIPAGEYLVRVEHIAIHEGH-VGKAQF 177
pyrenophora_trici_repentis_23/   109 LKNPS------TPGVDTAWESWQKDRLEWTIQRSVPAGEYLVRVEHIAIHEGH-VG---- 157
podospora_anseria_17/1-231       126 LCRGG-------GNVDTNWCSWQKDRLEFTIPPKTPPGEYLVRIEHIGLHEGH-VRRAQF 177
glomerrela_graminic_16/1-230     125 LCGGQ-------PNVDTSWCTWQKDRIEFTIPNRIPPGDYLVRVEHIGLHEAH-VGKAQF 176
glomerrela_graminic_31/1-230     125 LCGSN-------PSVDTDWCTWQKDRIEFTLPAATPPGDYLVRVEHIAIHEGH-VGKAQF 176
chaetomium_globosum_15/1-229     124 LCRGN-------AASDGNWCSYNKDRLEFVIPPKTPPGEYLVRVEHIGLHEGF-KNRAQF 175
neosartorya_fischeri_2/1-235     128 LCADTS-----KGIVDEDWCTWGKDTASFKIPEDTPAGQYLVRVEHIGLHRGF-LGESEF 181
aspergillus_tereus_12/1-235      128 VCSDIS-----DGLVDTDWCTWGKDTVKFKIPSDMPPGQYLVRVEHIGLHRGF-SGNSEF 181
aspergillus_favus_7/1-235        128 VCEDIS-----DGLKDTDWCTWGKDTASFKIPKDTPPGQYLVRVEHIGLHRGF-SGNSEF 181
aspergillus_fumingatus_6/1-235   128 LCADTS-----KGIKDEDWCTWGKDTASFKIPQDTPAGQYLVRVEHIGLHRGF-LGEAEF 181
4B5Q/1-217                       110 PQFE--------NGQLVF--DTTQQEVTFTIPKSLPSGQYLLRIEQIALHVASSYGGAQF 159
3EJA/1-208                       103 PTFG----------AQLTWPSTGKSSFAVPIPPCIKSGYYLLRAEQIGLHVAQSVGGAQF 152
3EII/1-208                       103 PTFG----------AQLTWPSTGKSSFAVPIPPCIKSGYYLLRAEQIGLHVAQSVGGAQF 152

                                              250       260       270       280       290       300
                                     ....|....|....|....|....|....|....|....|....|....|....|....|
serpula_lacrymans_5/1-229        176 YLSCAQINVQNGGNGTP--GPLVSIPGVYTGYEPGILINIYSPI------PANYT---QP 224
schizzophylum_commune_15/1-228   175 YLSCAQVKVTGGGNGTP--GPLVSIPGVYDGNEPGIKLNIYSPI------PANYS---QP 223
schizzophylum_commune_16/1-228   175 YLSCAQVKVTGGGNGSP--GPLVSIPGVYTGNEPGILINIYYPV------PANYT---MP 223
chaetomium_globosum_8/1-223      170 YISCAQIEVTGGGSTEP--SNKVSFPGAYKASDPGILININWPI------PTSYQ---NP 218
pyrenophora_trici_repentis_13/   167 YLSCAQIEVTGGGSKTP--TNLVAFPGAYSATDPGILININYPI------PTSYK---NP 215
pyrenophora_teres_11/1-209       167 YL-----------SKAP--TNLVAFPGAYSATDPGILININYPI------PTSYK---NP 204
Phaeospheraria_nodorum_18/1-21   165 YLSCAQLEVTGGGSTAP--KNLVAFPGAYSATDPGILININYPI------PTSYK---NP 213
podospora_anseria_18/1-223       170 YISCAQIGVTGGGNTEP--SNKVSFPGAYSASDPGIQININWPI------PTSYR---NP 218
thievela_terestis_18/1-224       171 YISCAQLSVTGGGSTEP--PNKVAFPGAYSATDPGILINIYYPV------PTSYQ---NP 219
myceliophthora_thermophilia_21   170 YISCAQLQVTGGGSTEP--SQKVSFPGAYKSTDPGILININYPV------PTSYQ---NP 218
TYPE1:NCU03328/1-229             174 YISCAQLSITGGGSTEPGANYKVSFPGAYKASDPGILININYPV------PTSYK---NP 224
emmericella_nidulans__3/1-229    176 YLSCGQVSVTGGGNGDP--APLVAFPGAYDPTDPGILINIYWPV------PTNYT---PP 224
aspergillus_tereus_4/1-228       175 YISCGQVTVTGGGDGTP--GPLVAFPGAYSPTDPGILIDIYYPV------PTEYT---PP 223
podospora_anseria_11/1-231       177 YLSCAQISVTGGSGTLN-TGQLVSFPGAYKATDPGILFQLYWPT------PTSYT---NP 226
glomerrela_graminic__6/1-230     177 YLSCAQVSLSGGGSTTP--SGLVSFPGAYKATDPGILFKLYWPI------PTSYT---NP 225
arthrobotrys_oligospora_11/1-1   136 YLACGQINVTGGGSKAG--TPLVSFPGAYKATDPGLLINIYWPI------PTSYT---NP 184
pyrenophora_trici_repentis_20/   175 YLSCAQLKVENGGNGSP--GPKVAFPGAYKATDPGIMINIYYPV------PTSYT---AP 223
Phaeospheraria_nodorum_28/1-22   173 YISCAQIKVENGGNGSP--TDLVAFPGAYKATDPGILINVYYPV------PTSYT---PP 221
myceliophthora_thermophilia_16   176 YIACAQLSVTGGSGTLN-TGSLVSLPGAYKATDPGILFQLYWPI------PTEYI---NP 225
thievela_terestis_11/1-231       177 YLACAQLTVTGGTGTLN-TGELVAFPGAYSATDPGILFQLYWPI------PTSYT---NP 226
chaetomium_globosum_24/1-226     172 YISCAQLSVTGGSGTIN-TGSLVSFPGAYKATDPGILFQLYWPI------PTSYV---NP 221
chaetomium_thermohilum_14/1-23   177 YIACAQLTVTGGTGTLN-TGQLVSFPGAYKATDPGILFQLYWPP------PTSYI---NP 226
TYPE1:NCU02344/1-232             177 YIACAQLNVTGGTGTINTSGKLVSFPGAYKATDPGLLFNLYYPA------PTSYT---NP 227
myceliophthora_thermophilia_2/   170 YISCAQITVTGGGSANP--GPTVSIPGAFKETDPGYTVNIYNN-------FHNYT---VP 217
sodaria_macrospora_4/1-217       166 YLSCAHISVTGGGSAS---PATVSIPGAFKETDPGYTANIYSN-------FNSYT---VP 212
neurospora_tetrasperma_12/1-21   167 YLSCAHISVTGGGSAS---PATVSIPGAFKETDPGYTVNIYSN-------FNNYT---VP 213
TYPE1:NCU00836/1-218             167 YLSCAHISVTGGGSAS---PATVSIPGAFKETDPGYTVNIYSN-------FNNYT---VP 213
thievela_terestis_7/1-223        171 YISCAQITVTGGGNGNP--GPTALIPGAFKDTDPGYTVNIYTN-------FHNYT---VP 218
podospora_anseria_5/1-221        169 YISCAQINVSGGGNSSP--SPTVKIPGAFKATDPGYTANIYNN-------LQSYT---VP 216
leptosphaeria_maculans_15/1-17   124 YMECAQINVTGGGNAN---PPTISFPGAYKGSDPGIKFQLYWPK------PTSYS---IP 171
glomerrela_graminic_9/1-232      179 YMECAQINVVGGTAAKT--PSTVSFPGAYKGTDPGITLSIYYPP------VTNYV---IP 227
glarea_lozoyensis_6/1-240        188 YMECAQINVTGGSGTAA--PSTVAFPGAYKSTDPGILINIYQT-------LPSYK---IP 235
TYPE2:NCU02240/1-235             179 YMECAQINIVGGTGGTALPSTTYSIPGIYKATDPGLLVNIYSMS-----PSSTYT---IP 230
botryotinia_fuckeliana_2/1-234   182 YMECAQINVSGGTGTST--PSTVSFPGAYGQSDPGILINIYQT-------LTTYT---IP 229
botryotinia_fuckeliana_12/1-24   188 YMECAQINVSGGTGTST--PSTVSFPGAYGQSNPGILINIYQT-------LTTYT---IP 235
sclerotinia_sclerot_5/1-240      188 YMECAQINVSGGTGTSS--PSTVSFPGAYAQNDPGILINIYQT-------LSSYP---IP 235
Phaeospheraria_nodorum_9/1-227   175 YMECAQINIVGGSADKT--PATVSFPGAYKQNDVGIIYNLYSG-------QKTYT---SP 222
chaetomium_thermophilum_18/1-2   182 YMECAQLNIVGGTGTKK--PSTVSFPGAYSGNDPGVKINIWWPP------VTSYK---VP 230
cholletotrichum_higginsianum_3   182 YMECAQINVVGGTGTKT--PSTVSFPGAYKSSDPGVTISIWWPP------VTNYV---IP 230
podospora_anseria_30/1-234       181 YMECAQLNIVGGTGSKT--PSTVAFPGAYSGSHPGVKISIYWPP------VTNYQ---IP 229
myceliophthora_thermophilia_10   182 YMECAQLNIVGGTGSKT--PSTVSFPGAYKGTDPGVKINIYWPP------VTSYQ---IP 230
neurospora_crassa_1/1-236        183 YMECAQLNVVGGTGAKT--PSTVSFPGAYSGSDPGVKISIYWPP------VTSYT---VP 231
TYPE2:NCU01050/1-226             183 YMECAQLNVVGGTGAKT--PSTVSFPGAYSGSDPGVKISIYWPP------VT-------- 226
neurospora_tetrasperma_1/1-236   183 YMECAQLNVVGGTGAKT--PSTVSFPGAYSGSDPGVKISIYWPP------VTSYT---VP 231
sodaria_macrospora_11/1-236      183 YMECAQLNVVGGTGAKT--PSTVSFPGAYSGSDPGVKINIYWPP------VTSYT---VP 231
Paravalsa_indica_8/1-210         159 YMTCFQLSVGGSGTGS---PSTVKLPGAYGASDPGILYTLWMS-------SKIYT---IP 205
pyrenophora_trici_repentis_11/   176 YVSCFQVKVTGGGSAT---PAGVSFPGGYKTSDSLFTQSIYNS-------DFKYV---SP 222
leptosphaeria_maculans_11/1-22   176 YVSCFQVKVTGGGSAN---PTGVSFPGGYKISDPLFTKSIYDS-------SFNAN----- 220
Phaeospheraria_nodorum_14/1-22   176 YVSCFQVKVTGGGSAN---PAGVSFPGAYKLSDGLFTKAIYDS-------SFKYV---SP 222
pyrenochaeta_lyocope_1/1-227     176 YVSCFQVKVTNGGSAS---PSGVSFPGAYKNSDGLFTKSIWDS-------SFKYV---SP 222
pyrenophora_terestis_25/1-227    176 YVSCFQVKVTGGGSAT---PAGVSFPGGYKTSDSLFTQSIYNS-------DFKYT---SP 222
```

```
glarea_lozoyensis_1/1-214          163 YMTCYQVTVTGTGTAN---PAGVKFPGAYKANDPGIQINIYNN-------LQSYT---IP 209
myceliophthora_thermophilum_12     188 YMTCYQLTVTGSGSAS---PPTVSFPGAYKATDPGILVNIHAP-------LSGYT---VP 234
chaetomium_thermophilum_7/1-24     189 YMTCYQLTVEGGGNAN---PATVKFPGAYSATDPGILVNIHSA-------MNNYV---VP 235
podospora_anseria_15/1-234         185 YMTCYQITVSGGGNAS---PATVRLPGAYGASEG--QVNIHAA-------LTSYT---AP 229
thievela_terestis_10/1-235         184 YMSCYQITVSGGGSAS---PATVKFPGAYSANDPGIHINIHAA-------VSNYV---AP 230
podospora_anseria_24/1-240         189 YMTCYQITVSGGGSAS---PPTVSIPGHFKASDPGVQVNIHGA-------MTNYV---IP 235
pyrenophora_teres_17/1-233         182 YMTCFQITVTGGGSSS---PAGVSFPGAYKATDPGIQINIYQN-------LASYV---AP 228
pyrenophora_trici_repentis__8/     182 YMTCYQITVTGGGSSS---PAGVSFPGAYKAADPGIQINIYQN-------LASYA---AP 228
chaetomium_globosum_22/1-239       188 YMTCYQLSVTGGGSAT---PATVSFPGAYKSSDPGILVNIHSA-------MSGYT---VP 234
thievela_terestis_17/1-240         189 YMSCYQLTVTGSGSAT---PSTVNFPGAYSASDPGILINIHAP-------MSTYV---VP 235
glomerrela_graminic_32/1-232       181 YMSCYQLKVEGSGSAK---PTGVTFPGAYKATDPGLLINIYNK-------ITNYV---VP 227
verticillium_dahliae_23/1-243      184 YTSCVNLRVTRGGNFAP--EETFAIPGVYEANDPAITIMIYGNTGKPDNNLKPYT---PA 238
verticilium_albo_atrum_15/1-21     162 YTSCVNLRVTRGGNFAP--AETFAIPGVYEANDPAITIMIYGNT------GEAGQ---QF 210
glomerrela_graminic_17/1-244       185 YISCANVRVTGSGTFSP--SSTVSFPGAYQQSDPSIVINIYGAGGVPNNGGKAYS---AP 239
chaetomium_globosum_23/1-244       185 YQSCAQINVSGSGSLSP--SDTVSFPGAYSASDPGILTSIYGSTGKPDNDGKAYT---AP 239
podospora_anseria_29/1-244         185 YKSCAQINISGSGSFTP--SQTVSFPGAYQANHPGILTSIYGLTGQPDNGGKPYS---IP 239
chaetomium_thermophilum_15/1-2     185 YQSCAQINVSGGGSFTP--SQTVSFPGAYSQNDPGILVNIYGLTGQPDNGGKPYQ---AP 239
sodaria_macrospora_16/1-245        186 YQSCAQINVSGSGSYTP--SSTVSFPGAYKSNDPGILINIYGKLGQPDMDGKPYT---VP 240
myceliophthora_thermophilia_5/     185 YQSCAQINVSGGGSFTP--ASTVSFPGAYSASDPGILINIYGATGQPDNNGQPYT---AP 239
thievela_terestis_2/1-244          185 YQSCAQINVSGSGSFSP--SQTVSIPGVYSATDPSILINIYGSTGQPDNGGKAYN---PP 239
Paravalsa_indica_13/1-241          182 YISCAQINVTSGGSFSP--SSTVSFPGAYSSSDPGILISIYGSSGQPDNGGRAYT---PP 236
Paravalsa_indica_12/1-241          182 YISCAQINVTSGGSFSP--SSTVSFPGAYSPTDPGILINIYGSSGQPDNGGKAYT---PP 236
myceliophthora_thermophilia_6/     182 YMGCAQIEVTGSGTNSG--SDFVSFPGAYSANDPGILLSIYDSSGKPNNGGRSYP---IP 236
chaetomium_globosum_31/1-241       182 YMECAQVEITGSGTNSG--SNFVSFPGAYPANHPGIVVSIYDSTGKPTMNGRTYQ---IP 236
podospora_anseria_4/1-241          182 YMECAQIEITGSGTNTG--SNFVSFPGAYTADHPGILVSIYDLQGRPTNGGRPYT---IP 236
sodaria_macrospora_2/1-240         182 YIGCAQINVTGGGNAS---PSTVSFPGAYSASDPGILINIYGTGGSTNNGGRAYQ---IP 235
neurospora_tetrasperma_16/1-24     182 YIGCAQINVTGGGSASP--SNTVSFPGAYSASDPGILINIYGGSGTTDNGGKPYQ---IP 236
neurospora_tetrasperma_14/1-24     182 YIGCAQINVTGGGSTSP--SNTVSFPGAYSASDPGILINIYGGSGKTDNGGKPYQ---IP 236
TYPE2:NCU02916/1-241               182 YIGCAQINVTGGGSASP--SNTVSFPGAYSASDPGILINIYGGSGKTDNGGKPYQ---IP 236
4EIR/1-223                         168 YMECAQLNVVGGTGAKT--PSTVSFPGAYSGSDPGVKISIYWPP------VTAYT---VP 216
pyrenophora_trici_repentis_22/     163 YTGCAQIFLQSSGNLVP--ESTVSIPGIMKYNTPPTDFDIYNTP------ASKYQ---IP 211
verticillium_dahiae_24/1-222       169 SLACAQLQVTGSGTDFP--ESFVSFPGAYKADDAGLLFDIYTST------TKSYP---YP 217
verticilium_albo_atrum_16/1-21     164 SLACAQLQVTGSGPDFP--ESFVSFPGAYKADDVGLLFDIYAGT------NNPYP---YP 212
verticillium_dahliae_26/1-221      169 YIACAQVEVSGGGNGTP--GPLVAFPGEYSKEDPGLAVNIYAAS-------QRYE---YP 216
3ZUD/1-228                         175 YPQCINLQVTGGGSDN---PAGTLGTALYHDTDPGILINIYQK-------LSSYI---IP 221
TYPE3:NCU07898/1-239               187 YPGCHQLQVTGSGTKTP-SSGLVSFPGAYKSTDPGVTYDAYQA--------ATYT---IP 234
2YET/1-228                         175 YPQCINLQVTGGGSDN---PAGTLGTALYHDTDPGILINIYQK-------LSSYI---IP 221
thermoascus_auranti_1/1-228        175 YPQCINLQVTGGGSDN---PAGTLGTALYHDTDPGILINIYQK-------LSSYI---IP 221
4EIS/1-224                         172 YPGCHQLQVTGSGTKTP-SSGLVSFPGAYKSTDPGVTYDAYQA--------ATYT---IP 219
giberella_zeae_7/1-251             200 YPQCFNLKITGDGSDS---PSGYLGTELYDAADPGILVNVYSS-------SVDYE---VP 246
fusarium_oxysporum_3/1-252         201 YPQCFNLRITGDGSDS---PSGYLGTELYDVEDPGILVNVYSS-------SVDYE---VP 247
nectria_heamatococcuss_1/1-252     201 YPQCFNLKITGSGSDN---PPGYLGTELYDANDPGILVNIYGN-------LPNYQ---VP 247
verticilium_albo_atrum_13/1-24     191 YPQCINLEISGGGSAS---PSGVAGTSLYTETDPGVLFNIYTA--------TEYP---IP 236
verticillium_dahliae_4/1-241       191 YPQCINLEISGGGSAS---PSGVAGTSLYTETDPGVLFNIYTA--------TEYP---IP 236
neurospora_tetrasperma__2/1-24     189 YPQCFNLKVEGSGSTV---PAGVAGTELYKATDAGILFDIYKN-------DISYP---VP 235
TYPE3:NCU07760/1-240               189 YPQCFNLKVEGSGSTV---PAGVAGTELYKATDAGILFDIYKN-------DISYP---VP 235
magna_porte_oryzae_16/1-243        192 YPQCFNLEITGSGSTQ---PAGVAGTSLYTANDPGILFQLYQA-------PISYR---VP 238
podospora_anseria_31/1-244         193 YPQCLNLRIVGNGNNS---PAGVAGTSLYRANDAGILFNPYVA-------SPNYP---VP 239
hypocrea_orientalis_1/1-246        195 YPQCFNLAVSGSGSLQ---PSGVKGTALYHSDDPGVLINIYTS-------PLAYT---IP 241
trichoderma_SP_SSL_1/1-246         195 YPQCFNLAVPGSGSLQ---PSGVKGTALYHSDDPGVLINIYTS-------PLAYT---IP 241
Hypocrea_virens_3/1-246            195 YPQCFNLAVTGTGSLQ---PTGVLGTKLYQESDPGILFNIYTS-------PLTYT---IP 241
trichoderma_atroviride_2/1-246     195 YPQCFNLAVTGTGSLQ---PSGVLATDLYHETDPGILFNIYTS-------PLTYI---IP 241
hypocrea_rufa_1/1-246              195 YPQCFNIAVSGSGSLQ---PSGVLGTDLYHATDPGVPINIYTS-------PLNYI---IP 241
hypocrea_rufa_2/1-246              195 YPQCFNIAVSGSGSLQ---PSGVLGTDLYHATDPGVLINIYTS-------PLNYI---IP 241
trichoderma_saturnusporum_1/1-     195 YPQCFNLAVSGSGSLK---PSGVKGTALYHATDPGVLINIYTS-------PLNYI---IP 241
aspergillus_kawachii_37/1-247      196 YPQCFNLQVTGTGTAT---PSGVKGTELYTATENGILVNIYST-------LTTYT---VP 242
aspergillus_tereus_6/1-245         195 YMQCFNLRVTGGGSQS---PAGVPATELYTSTDPGILVDIYNS--------LTYT---VP 240
neosartorya_fischeri_4/1-247       196 YPQCFNLQITGSGTAQ---PSGVKGTKLYSPTDPGILVNIYNS-------LSTYI---VP 242
aspergillus_fumingatus_4/1-247     196 YPQCFNLQITGSGTAQ---PSGVKGTELYSPTDPGILVNIYNA-------LSTYI---VP 242
aspergillus_tereus_10/1-245        195 YPQCFNLQITGSGTVK---PSGVLGTALYSPTDPGILVNIYNS--------LNYI---VP 240
chaetomium_globosum_5/1-220        197 YPQCFNLQSSSADHRA---RSAITGSA--------------------------------- 220
TYPE3:NCU05969/1-243               192 YPQCFNIQVTGSGTEK---PAGVKGTALYKPDDAGISVNIYQS-------LSSYS---IP 238
2VTC/1-228                         176 YPQCVNIAVTGSGTKAL--PAGTPATQLYKPTDPGILFNPYTT-------ITSYT---IP 223
aspergillus_fumingatus_1/1-245     194 YPQCINLKVTGGGSDK---PAGTLGTALYKNTDAGILVNIYQS-------LSSYE---IP 240
neosartorya_fischeri_1/1-245       194 YPQCINLKVTGGGSDK---PAGTLGTALYKNTDAGILVNIYQS-------LSSYD---IP 240
aspergillus_niger_1/1-245          194 YPQCINLNVTGSGTAT---PSGTLGTALYMDTDPGIYIDIWQS-------ISSYT---IP 240
aspergillus_kawachii_40/1-245      194 YPQCINLNVTGSGTAT---PSGTLGTALYKDTDPGIYIDIWQS-------ISSYT---IP 240
aspergillus_tereus_5/1-232         194 YPQCINIEITGGGSTK---PSGVSATIFYKNTDPGIKFR--------------------- 229
emmericella_nidulans_9/1-245       194 YPQCVNLEVTGGGSAS---PSGTVGTELYTPTDPGILVNIYTS-------LDSYT---IP 240
penicillum_chrysogenum_2/1-245     194 YPQCLNLKVTGGGSDV---PEGVVGTELYKPDDAGILVSIYNQ-------LKDYT---IP 240
aspergillus_niger_2/1-245          194 YPQCLNLKVTGSGSST---YSGTKGEALYKDTDPGILVNIYET-------LSSYD---IP 240
aspergillus_niger_12/1-244         194 YPQCLNLKVTGSGSST---YSGTKGEALYKDTDPGILVNIYET-------LSSYD---IP 240
aspergillus_kawachii_38/1-245      194 YPQCLNLKVTGSGSST---YSGTKGEALYKDTDPGILVNIYQS-------LSSYD---IP 240
zea_mys_1/1-245                    194 YPQCLNLKVTGSGSST---YSGTKGEALYKDTDPGILVNIYET-------LSSYD---IP 240
aspergillus_clavatus_6/1-241       190 YPQCINLKVTGGGSDK---PVGTLGTKLYKATDPGILVNIYGS-------LTSYT---IP 236
aspergillus_tereus_8/1-241         190 YPQCMNLKVTGGGSDK---PAGTLGTALYKDTDPGILVNIYQT-------LSSYV---IP 236
aspergilus_oryzae_7/1-242          191 YPQCLNFKVTGGGSDK---PEGTLGTALYKDTDPGIEINIYQT-------LSSYT---IP 237
```

```
aspergillus_favus_5/1-242          191 YPQCLNFKVTGGGSDK---PEGTLGTALYKDTDPGIQINIYQT-------LTSYT---IP 237
glomerrela_graminic_4/1-242        191 YPQCLNIEVTGSGSEE---PAGVKGTALYTPKDAGILVSVYNN-------LSKYQ---IP 237
aspergillus_fumingatus_3/1-238     186 YYTCAQVKVVGGGTGTP--GPTIKFPGGYKKDDPSFNFSLWNG-------YKDYP---MP 233
cholletotrichum_higginsianum_2     186 YNSCAQVKITGGGNGTP--GPMIKFPGGYKKSDPSFNFSIYGG-------YKAYP---MP 233
glomerrela_graminic_7/1-238        186 YNSCAQIKVTGGGNGTP--GPMIRFPGGYKKTDPSFNFSIYNG-------YKDYP---MP 233
aspergillus_clavatus_5/1-238       186 YYSCAQVKIVGGGKGKP--GPMIKFPGGYKKTDPSFNFSIYNG-------YKPYP---MP 233
aspergilus_oryzae_5/1-238          186 YYECAQVKVVGGGNGTP--GPTVKFPGAYKKTDPSFTYSVWGG-------YKDYP---MP 233
aspergillus_favus_3/1-238          186 YYECAQVKVVGGGNGTP--GPTVKFPGAYKKTDPSFTYSVWGG-------YKDYP---MP 233
aspergillus_niger_9/1-238          186 YYECAQVKVTGGGNGNP--GPTIKFPGGYKKDDPSFNFSIWGG-------MKDYP---MP 233
emmericella_nidulans__6/1-238      186 YYECAQVKVTGGGNGNP--QDTIKFPGGYQKDDPSFNFSVWGG-------MKDYP---MP 233
aspergillus_clavatus_1/1-238       186 YNTCAQVKVVGGGNGTP--GPTVKFPGAYKKNDPSFNFSIYQG-------YKEYP---MP 233
neosartorya_fischeri_7/1-238       186 YYTCAQVKVVGGGNGNP--GPTIKFPGGYKKDDPSFNFSLWNG-------YKDYP---MP 233
penicillum_chrysogenum_4/1-236     184 YYSCAQVKVVGGGNGTP--GPTVKFPGAYKKDDPSFNFSIWNG-------YKEYP---MP 231
verticilium_albo_atrum_17/1-21     159 YYTCVQVKVTGGGNGTP--GPTVKFPGAYKKDDPSFNFSIYGG-------VKDYP---MP 206
verticillium_albo_atrum_17/1-2     159 YYTCVQVKVTGGRNGTP--GPTVKFPGAYKKDDSSFNFSIYGG-------VKDYP---MP 206
pyrenophora_teres_22/1-230         178 YMECFQLNIQSSGTGKL--GPTVKIPGLYSAQDPGIAFNKWVN-------PKSYV---VP 225
Phaeospheraria_nodorum_4/1-230     178 YMECFQLKIESSGTGKL--GPTAKIPGIYKATDPGIAFDKWTN-------PKSYT---MP 225
pyrenophora_trici_repentis_23/     157 --ECFQLNIQSSGTGKL--GPTVKIPGLYSAQDPGIAFNKWVN-------PKSYV---VP 203
podospora_anseria_17/1-231         178 YITCAQLRITGPGGNP--SPLVRIPGLYNANDPGIAYNKWTNN------PAAYR---MP 226
glomerrela_graminic_16/1-230       177 YMECAHLKITGSGGTP--GPLVKIPGIYKASDPGIAYNKWTNT------PAQYI---MP 225
glomerrela_graminic_31/1-230       177 YMECAHIKVTGNGAGTP--GPLVKIPGMYSSTDPGIAYNKWTGN------PAPYV---IP 225
chaetomium_globosum_15/1-229       176 YMECAQLKITGPGGTP--GPLAKIPGIYKASDPGIAYDKWKSN------PAPYQ---MP 224
neosartorya_fischeri_2/1-235       182 YFTCAQIEVTGSGSGSP--SPVVKIPGVYKPEDPNIHFNIWYPT------PTAYN---LP 230
aspergillus_tereus_12/1-235        182 YFTCAQIEVTGSGTGTP--AEVAKIPGIYKPDDANIHFNIYYPT------PTAYD---LP 230
aspergillus_favus_7/1-235          182 YFTCAQIEVTGSGSGVP--GPLVKIPGMYKPEDPNIHFDIYYPV------PTSYD---LP 230
aspergillus_fumingatus_6/1-235     182 YFTCAQIEVTGSGSGSP--SPTVKIPGVYKPDDPNVHFNIWYPT------PTAYS---LP 230
4B5Q/1-217                         160 YIGCAQLNVENGGNGTP--GPLVSIPGVYTGYEPGILINIYNL-------PKNFTGYPAP 210
3EJA/1-208                         153 YISCAQLSVTGGGSTEP--PNKVAFPGAYSATDPGILINIYYPV------PTSYQ---NP 201
3EII/1-208                         153 YISCAQLSVTGGGSTEP--PNKVAFPGAYSATDPGILINIYYPV------PTSYQ---NP 201


                                       ....|..
serpula_lacrymans_5/1-229          225 GPAVW-- 229
schizzophylum_commune_15/1-228     224 GPAVW-- 228
schizzophylum_commune_16/1-228     224 GPAVW-- 228
chaetomium_globosum_8/1-223        219 GPPVF-- 223
pyrenophora_trici_repentis_13/     216 GPPTF-- 220
pyrenophora_teres_11/1-209         205 GPATF-- 209
Phaeospheraria_nodorum_18/1-21     214 GPDTF-- 218
podospora_anseria_18/1-223         219 GPPVF-- 223
thievela_terestis_18/1-224         220 GPAVF-- 224
myceliophthora_thermophilia_21     219 GPAVF-- 223
TYPE1:NCU03328/1-229               225 GPSVF-- 229
emmericella_nidulans__3/1-229      225 GPKVW-- 229
aspergillus_tereus_4/1-228         224 GPAVW-- 228
podospora_anseria_11/1-231         227 GPAPV-- 231
glomerrela_graminic__6/1-230       226 GPAPF-- 230
arthrobotrys_oligospora_11/1-1     185 GPKIF-- 189
pyrenophora_trici_repentis_20/     224 GPAVW-- 228
Phaeospheraria_nodorum_28/1-22     222 GPKVW-- 226
myceliophthora_thermophilia_16     226 GPAPV-- 230
thievela_terestis_11/1-231         227 GPAPV-- 231
chaetomium_globosum_24/1-226       222 GPPVV-- 226
chaetomium_thermohilum_14/1-23     227 GPAPV-- 231
TYPE1:NCU02344/1-232               228 GPAVA-- 232
myceliophthora_thermophilia_2/     218 GPAVF-- 222
sodaria_macrospora_4/1-217         213 GPAEF-- 217
neurospora_tetrasperma_12/1-21     214 GPEVF-- 218
TYPE1:NCU00836/1-218               214 GPEVF-- 218
thievela_terestis_7/1-223          219 GPEVF-- 223
podospora_anseria_5/1-221          217 GPKVF-- 221
leptosphaeria_maculans_15/1-17     172 GPRPF-- 176
glomerrela_graminic_9/1-232        228 GPQKF-- 232
glarea_lozoyensis_6/1-240          236 GPPVF-- 240
TYPE2:NCU02240/1-235               231 GPAKF-- 235
botryotinia_fuckeliana_2/1-234     230 GPTPF-- 234
botryotinia_fuckeliana_12/1-24     236 GPTPF-- 240
sclerotinia_sclerot_5/1-240        236 GPTPF-- 240
Phaeospheraria_nodorum_9/1-227     223 GPSVF-- 227
chaetomium_thermophilum_18/1-2     231 GPPVF-- 235
cholletotrichum_higginsianum_3     231 GPDVF-- 235
podospora_anseria_30/1-234         230 GPSVF-- 234
myceliophthora_thermophilia_10     231 GPGVF-- 235
neurospora_crassa_1/1-236          232 GPSVF-- 236
TYPE2:NCU01050/1-226               226 ------- 226
neurospora_tetrasperma_1/1-236     232 GPSVF-- 236
sodaria_macrospora_11/1-236        232 GPSVF-- 236
```

Paravalsa_indica_8/1-210	206	GPSSV--	210
pyrenophora_trici_repentis_11/	223	GPAVY--	227
leptosphaeria_maculans_11/1-22	220	--ALY--	223
Phaeospheraria_nodorum_14/1-22	223	GPAVY--	227
pyrenochaeta_lyocope_1/1-227	223	GPAVW--	227
pyrenophora_terestis_25/1-227	223	GPAVY--	227
glarea_lozoyensis_1/1-214	210	GPAVW--	214
myceliophthora_thermophilum_12	235	GPAVY--	239
chaetomium_thermophilum_7/1-24	236	GPPVY--	240
podospora_anseria_15/1-234	230	GPAVY--	234
thievela_terestis_10/1-235	231	GPAVY--	235
podospora_anseria_24/1-240	236	GPAVY--	240
pyrenophora_teres_17/1-233	229	GPAVI--	233
pyrenophora_trici_repentis__8/	229	GPAVI--	233
chaetomium_globosum_22/1-239	235	GPAVY--	239
thievela_terestis_17/1-240	236	GPTVY--	240
glomerrela_graminic_32/1-232	228	GPAVY--	232
verticillium_dahliae_23/1-243	239	GPRPI--	243
verticilium_albo_atrum_15/1-21	210	-------	210
glomerrela_graminic_17/1-244	240	GPRPI--	244
chaetomium_globosum_23/1-244	240	GPRPI--	244
podospora_anseria_29/1-244	240	GPAPI--	244
chaetomium_thermophilum_15/1-2	240	GPAPI--	244
sodaria_macrospora_16/1-245	241	GPAPI--	245
myceliophthora_thermophilia_5/	240	GPAPI--	244
thievela_terestis_2/1-244	240	GPAPI--	244
Paravalsa_indica_13/1-241	237	GPAVI--	241
Paravalsa_indica_12/1-241	237	GPAVI--	241
myceliophthora_thermophilia_6/	237	GPRPI--	241
chaetomium_globosum_31/1-241	237	GPAPI--	241
podospora_anseria_4/1-241	237	GPAPL--	241
sodaria_macrospora_2/1-240	236	GPPAV--	240
neurospora_tetrasperma_16/1-24	237	GPALF--	241
neurospora_tetrasperma_14/1-24	237	GPALF--	241
TYPE2:NCU02916/1-241	237	GPALF--	241
4EIR/1-223	217	GPSVFTC	223
pyrenophora_trici_repentis_22/	212	GPPVA--	216
verticilium_dahiae_24/1-222	218	GPASP--	222
verticilium_albo_atrum_16/1-21	213	GPAVW--	217
verticillium_dahliae_26/1-221	217	GPEVW--	221
3ZUD/1-228	222	GPPLYTG	228
TYPE3:NCU07898/1-239	235	GPAVF--	239
2YET/1-228	222	GPPLYTG	228
thermoascus_auranti_1/1-228	222	GPPLYTG	228
4EIS/1-224	220	GPAVF--	224
giberella_zeae_7/1-251	247	GPTIC--	251
fusarium_oxysporum_3/1-252	248	GPTIV--	252
nectria_heamatococcuss_1/1-252	248	GPTIV--	252
verticilium_albo_atrum_13/1-24	237	GPPLY--	241
verticilium_dahliae_4/1-241	237	GPPLY--	241
neurospora_tetrasperma__2/1-24	236	GPSLI--	240
TYPE3:NCU07760/1-240	236	GPSLI--	240
magna_porte_oryzae_16/1-243	239	GPPMM--	243
podospora_anseria_31/1-244	240	GPALI--	244
hypocrea_orientalis_1/1-246	242	GPSVV--	246
trichoderma_SP_SSL_1/1-246	242	GPSVV--	246
Hypocrea_virens_3/1-246	242	GPTVV--	246
trichoderma_atroviride_2/1-246	242	GPTVV--	246
hypocrea_rufa_1/1-246	242	GPTVV--	246
hypocrea_rufa_2/1-246	242	GPTVV--	246
trichoderma_saturnusporum_1/1-	242	GPTVV--	246
aspergillus_kawachii_37/1-247	243	GPTAY--	247
aspergillus_tereus_6/1-245	241	GPSMI--	245
neosartorya_fischeri_4/1-247	243	GPTLI--	247
aspergillus_fumingatus_4/1-247	243	GPTLI--	247
aspergillus_tereus_10/1-245	241	GPTPI--	245
chaetomium_globosum_5/1-220	220	-------	220
TYPE3:NCU05969/1-243	239	GPALI--	243
2VTC/1-228	224	GPALW--	228
aspergillus_fumingatus_1/1-245	241	GPALY--	245
neosartorya_fischeri_1/1-245	241	GPALY--	245
aspergillus_niger_1/1-245	241	GPTLY--	245
aspergillus_kawachii_40/1-245	241	GPTLY--	245
aspergillus_tereus_5/1-232	229	--HLL--	232
emmericella_nidulans_9/1-245	241	GPALW--	245
penicillum_chrysogenum_2/1-245	241	GPALY--	245
aspergillus_niger_2/1-245	241	GPAMY--	245

aspergillus_niger_12/1-244	241	GPAI---	244
aspergillus_kawachii_38/1-245	241	GPAMY--	245
zea_mys_1/1-245	241	GPAMY--	245
aspergillus_clavatus_6/1-241	237	GPALW--	241
aspergillus_tereus_8/1-241	237	GPALY--	241
aspergilus_oryzae_7/1-242	238	GPALY--	242
aspergillus_favus_5/1-242	238	GPALY--	242
glomerrela_graminic_4/1-242	238	GPALP--	242
aspergillus_fumingatus_3/1-238	234	GPTVW--	238
cholletotrichum_higginsianum_2	234	GPAVW--	238
glomerrela_graminic_7/1-238	234	GPAVW--	238
aspergillus_clavatus_5/1-238	234	GPKVW--	238
aspergilus_oryzae_5/1-238	234	GPQVW--	238
aspergillus_favus_3/1-238	234	GPQVW--	238
aspergillus_niger_9/1-238	234	GPAVW--	238
emmericella_nidulans__6/1-238	234	GPAVY--	238
aspergillus_clavatus_1/1-238	234	GPAVW--	238
neosartorya_fischeri_7/1-238	234	GPAVW--	238
penicillum_chrysogenum_4/1-236	232	GPEVW--	236
verticilium_albo_atrum_17/1-21	207	GPAVW--	211
verticillium_albo_atrum_17/1-2	207	GPAVW--	211
pyrenophora_teres_22/1-230	226	GPALW--	230
Phaeospheraria_nodorum_4/1-230	226	GPAKY--	230
pyrenophora_trici_repentis_23/	204	GPPLW--	208
podospora_anseria_17/1-231	227	GPAVW--	231
glomerrela_graminic_16/1-230	226	GPKVW--	230
glomerrela_graminic_31/1-230	226	GPAVW--	230
chaetomium_globosum_15/1-229	225	GPAVW--	229
neosartorya_fischeri_2/1-235	231	GPSVW--	235
aspergillus_tereus_12/1-235	231	GPSVW--	235
aspergillus_favus_7/1-235	231	GPSVW--	235
aspergillus_fumingatus_6/1-235	231	GPSVW--	235
4B5Q/1-217	211	GPAVWQG	217
3EJA/1-208	202	GPAVFSC	208
3EII/1-208	202	GPAVFSC	208
