# Supplementary material for: Bioinformatic characterization of type-specific sequence and structural features in auxiliary activity family 9 proteins
Source: Biotechnol Biofuels. 2016 Nov 9;9:239. doi: 10.1186/s13068-016-0655-2 (PMC5101804; doi:10.1186/s13068-016-0655-2)
Supplement: Supplementary file 3 — Additional file 3. Multiple sequence alignment of the Type 1 PMO sequences used in this study. [file 13068_2016_655_MOESM3_ESM.pdf]

```
                                            10        20        30        40        50        60
                                   ....|....|....|....|....|....|....|....|....|....|....|....|
aspergillus_fumingatus_3/1-238  1  MKLS--LLASV-ALVPFVSAHYFFDVLVIDGKETRS-NEFVRSNTRPAKYNPTKWKNIRD  56
neosartorya_fischeri_7/1-238    1  MKLS--LLAAV-ALVPFVSAHYFFDVLVIDGQETKS-NEFVRSNTRQAKYNPTKWKNIRD  56
aspergillus_clavatus_1/1-238    1  MKLS--LLALS-AIAPLVSAHYFFDVLVIDGKETKS-NEFVRSNTRPAKYNPTKWVNIRD  56
penicillum_chrysogenum_4/1-236  1  MKFS--LVALA-AIAPMVSAHYFFDTIVIDGKEAS---DSVRSNTRPAKYNPTKWVNTRD  54
aspergilus_oryzae_5/1-238       1  MKLS--FLALA-AIAPFVSAHYFFDTLIVDGKESSP-NQYVRSNTRPAKYNPTKWVNTRD  56
aspergillus_favus_3/1-238       1  MKLS--FLALA-AIAPFVSAHYFFDTLIVDGKESSP-NQYVRSNTRPAKYNPTKWVNTRD  56
aspergillus_niger_9/1-238       1  MKLT--LLTTA-LIAPLVSAHYFFDTLVIDGQETTP-NQYVRSNTRPAKYNPTKWKNTRD  56
emmericella_nidulans__6/1-238   1  MKLS--LLAAA-AIAPMVSAHYFFDTLVIDGQETTP-NQYVRSNTRPEKYNPTKWVNTRD  56
verticilium_albo_atrum_17/1-21  1  MRFS--ILAVA-ALTPLASAHYFFDKLIIDGVETRS-NEFVRSNTRPAKYNPTKWENVRD  56
verticillium_dahliae_25/1-211   1  MNFC--ILAAA-ALTPLASAHYFFDKLIIDDVETRS-NEFVRSSTPAAKYNPTKWENIRD  56
aspergillus_clavatus_5/1-238    1  MKLS--VITAV-ALVPFVSAHYFFDKLIVNGKETAS-FQYVRSNTRPNKYNPTKWNNVRD  56
colletotrichum_higginsianum_24  1  MKFS--AVVLA-AIAPLVSAHYFFDTLIVDGKATKS-FEYVRSNTRQAKYNPTKWENVRD  56
glomerrela_graminic_7/1-238     1  MKFS--AVALS-AVAPLASAHYFFDSFIVDGVATKS-FEYVRDNTRQAKYNPTKWENVRD  56
neosartorya_fischeri_2/1-235    1  MKLTSSILFSLASVTPLVSGHYVFSKLIVDGKPTQD-FEYIRKNTNGY--------MPTL  51
aspergillus_fumingatus_6/1-235  1  MKLTASILFSLASVTPLVSGHYVFSKLIVDGKPTQD-FEYIRRNTNNY--------MPTL  51
aspergillus_tereus_12/1-235     1  MKSFSPSLLLLASVAPLVSGHYVFSKFLVDGSVSQD-FEYIRRNSNGY--------MPTL  51
aspergillus_favus_7/1-235       1  MKLNLASLSFLASIAPLVSGHYVFSKLIVDGQTTKD-FEYIRENSNGY--------QPTL  51
podospora_anseria_17/1-231      1  MKLS-SFTILA-GLAAQAQAHYIFNILIVNGQRIGGEYTYVRRNSNSYN-------PAF-  50
chaetomium_globosum_15/1-229    1  MK---SFFVAA-ALAASAEAHYIFNILMVNDQRMGGEYTYVRRNSNSYM-------PTF-  48
glomerrela_graminic_16/1-230    1  MRISTFLPVLA---VPFVQCHYIFSQLIVNGNAVGSDYTYMRKNSNNYQ-------PSFT  50
glomerrela_graminic_31/1-230    1  MKGLLSFGLLA---APLVQGHYIFSQLFVDDKAAGGDYTYIRKNTNTYM-------PSFT  50
Phaeospheraria_nodorum_4/1-230  1  MVKAT-AVLAL-LAPL-TQAHYIFNQLLVDNKAIGGDYAYTRKNTNTYM-------PSFT  50
pyrenophora_teres_22/1-230      1  MKAV--ALFSL-LAPMVADAHYIFSSLIVNGAQVGGDFAYARKNSNSYM-------PSFT  50
pyrenophora_trici_repentis_23/  1  MKAV--ALFAL-VAPMVADAHYIFNLLTV----------------NNYM-------PSFT  34
podospora_anseria_5/1-221       1  MKVFPIVTALT-LGVADVSAHYIFQQFGVGSTKFGV-FEHIRKNNN----------HNSP  48
myceliophthora_thermophilia_2/  1  MKGLLGAAALS-LAVSDVSAHYIFQQLTTGGVKHAV-YQYIRKNTN----------YNSP  48
thievela_terestis_7/1-223       1  MKGLFSAAALS-LAVGQASAHYIFQQLSINGNQFPV-YQYIRKNTN----------YNSP  48
sodaria_macrospora_4/1-217      1  MK-LTVAAAL--LAASEASAHYIFQQVGTGTTVNPT-WKYIRQHTN----------YNSP  46
neurospora_tetrasperma_12/1-21  1  MK-LSVAAALS-LAASEASAHYIFQQVGAGTSVNPV-WKYIRKHTN----------YNSP  47
TYPE1:NCU00836/1-218            1  MK-LSVAAALS-LAASEASAHYIFQQVGAGTSVNPV-WKYIRKHTN----------YNSP  47
schizzophylum_commune_15/1-228  1  MRVPTL-LAAA-PLATTALAHYTLPVLVVNGEPSGE-WVNIRRTNNYY--------SQQP  49
schizzophylum_commune_16/1-228  1  MRLSSL-IATA-SLAASALAHYTLPTLIVNGQPSGE-WVNIRRTNNYN--------TQAP  49
serpula_lacrymans_5/1-229       1  MKSITLLSIAA-VLLPSVSAHYRWTSLVVGSTITTA-YEYVRQNTNDN--------S--P  48
4B5Q/1-217                      1  --------------------HYTFPDFIEPSGTVTGDWVYVRETQNHY--------SNGP  32
emmericella_nidulans__3/1-229   1  MKSGLL-FTTA-SLALTASAHYVFPALVQDGAATGD-WKYVRDWTGSY--------GNGP  49
aspergillus_tereus_4/1-228      1  MKGSYL-LGSA-MLAATSYAHYVFPALIKDGEATPD-WKYVRQWTGSY--------TNSP  49
verticillium_dahiae_24/1-222    1  MKTA---VVLG-LLAPFAFGNYLFRSITVNRGRSAD-WEFVRETANNP--------VSNP  47
verticillium_albo_atrum_16/1-21 1  --------MLG-LLAPFAFGNYLFRSITVNRGRSAD-WEFVRETANNP--------ASNP  42
verticillium_dahliae_26/1-221   1  MKSS---ALLP-LLSSGVLAHYFFPNTVVNGVQSAE-WEFIRETNNNP--------GAEP  47
pyrenophora_trici_repentis_20/  1  MKYSLA--ALL-AVASTASAHYTLPELTVKGVKTGQ-WAYVRKTSNYQ--------SNGP  48
Phaeospheraria_nodorum_28/1-22  1  MKTAFL--SLL-AAASTASAHYTFPGFISGSTVTSP-WEYVRKT--TN--------SNGP  46
thievela_terestis_11/1-231      1  MKLTTS--VAL-LAAAGAQAHYTFPQTDINGQLSGE-WVTIRETTNHY--------SHGP  48
chaetomium_thermophilum_14/1-2  1  MKFTTP--LAL-LAVVGVQAHYTFPRTKVNGVLSGE-YETVRLTANHW--------SHGP  48
pyrenophora_trici_repentis_13/  1  MKTSA--LLIA-AGATLASAHYTLPSI----NGDGT-WVHVRQAKNWQ--------DNGF  44
myceliophthora_thermophilia_16  1  MKLTSS--LAV-LAAAGAQAHYTFPRAGTGGSLSGE-WEVVRMTENHY--------SHGP  48
podospora_anseria_11/1-231      1  MKASTT--LAV-LAAAGAQAHYTFPGTKYNGVAQPQ-WDTVRITQNHY--------SNGP  48
chaetomium_globosum_24/1-226    1  MKFASS--VAL-LAAAGAQAHYTFPKTVVDGVTSAE-WETIRITENHY--------SHGP  48
TYPE1:NCU02344/1-232            1  MKFSSA--LAF-LAAAGAQAHYTFPKGYSTGAVSGE-YEHIRMTENHY--------NRGP  48
arthrobotrys_oligospora_11/1-1  1  M------------------------------------------TTNKY--------SHGP  10
glomerrela_graminic__6/1-230    1  MKFSAV--LVA-LAAASAEAHYTFGRLVYGGTTYPE-WQYVRKTLNFY--------SNGP  48
pyrenophora_teres_11/1-209      1  MKTSA--LILA-AGATLASAHYTLPSI----NSDGT-WVHVRQAKNWQ--------DNGF  44
Phaeospheraria_nodorum_18/1-21  1  MKTST--VLLA--AASVASAHYTLPIV----NGDSA-WTHVRQAKNWQ--------DNGF  43
thievela_terestis_18/1-224      1  MLANGA-IVFL-AAALGVSGHYTWPRV----NDGAD-WQQVRKADNWQ--------DNGY  45
3EJA/1-208                      1  --------------------HYTWPRVNDGADW-----QQVRKADNWQ--------DNGY  27
podospora_anseria_18/1-223      1  MLASL--ALVL-STALSATAHYTLPRV----GNGAD-WQHVRRADNWQ--------NNGF  44
TYPE1:NCU03328/1-229            1  MLPSI--SLLL-AAALGTSAHYTFPKVWANSGTTAD-WQYVRRADNWQ--------NNGF  48
chaetomium_globosum_8/1-223     1  MLTTT--FALL-GAALGASAHYTLPKV----GSGSD-WQHVRRADNWQ--------NNGF  44
myceliophthora_thermophilia_21  1  MLTTT--FALL-TAALGVSAHYTLPRV----GTGSD-WQHVRRADNWQ--------NNGF  44

                                            70        80        90       100       110       120
                                   ....|....|....|....|....|....|....|....|....|....|....|....|
aspergillus_fumingatus_3/1-238 57  DMTPDMPDFRCNKGAFTFAGQTGTAEVKAGSKLAMKLAVGATMQHPGPALVYMSKAPSSA 116
neosartorya_fischeri_7/1-238   57  NMTPDMTDFRCNKGAFTFAGKTGTAEVKAGSKLALKLAVGATMQHPGPALVYMSKAPSSA 116
aspergillus_clavatus_1/1-238   57  DMTPDVTDFRCNKGAFTFAGKTGTAEVKAGSKLAMKLAVGATFQHPGPGLVYMSKAPSAA 116
penicillum_chrysogenum_4/1-236 55  DMTPDMPDFRCNKGAFTFAGQTGTAEVKAGSKVAMKLAVGATMQHPGPALVYMSKAPTTA 114
aspergilus_oryzae_5/1-238      57  DMTPDMPDFRCNKGSFTFAGQTGTAEVKAGSKLAMKLGVGATMKHPGPALVYMSKAPSTA 116
aspergillus_favus_3/1-238      57  DMTPDMPDFRCNKGSFTFAGQTGTAEVKAGSKLAMKLGVGATMKHPGPALVYMSKAPSTA 116
aspergillus_niger_9/1-238      57  DMTPDMPDFRCNKGSFTFAGQTGTAEVKAGSKLAMKLGVGATMKHPGPGLVYMSKAPGSA 116
emmericella_nidulans__6/1-238  57  DMTPDMPDFRCNKGSFTFAGQTDTAEVKAGSKLAMKLGVGATMQHPGPGLVYMSKAPGAA 116
verticilium_albo_atrum_17/1-21 57  DMTPDVNDFRCNKGAFTFAGQTGTAEVKAGSKLAVKLAVGATMQHPGPALVYMSKAPATA 116
verticillium_dahliae_25/1-211  57  DMTPDVNDFRCKKGAFTFASQTGTAEVKAGSKLAVKLVVGATMQHPGPALVYMSKAPTTA 116
aspergillus_clavatus_5/1-238   57  NMTPDMSDFRCNKGAFTFAGKTGVAEIKAGSKVALKLAVNAHIQHPGPSLVYMSKAPNGV 116
colletotrichum_higginsianum_24 57  GMTPDLPDFRCNKGAFTFAAKTGTLEVKAGSKVAFKLGVGATMQHPGPAIVYMSKAPSTA 116
glomerrela_graminic_7/1-238    57  DMTPDVNDFRCNKGAFTFAGKTRTMEVKAGSKVGFKLGVGATMQHPGPGLAYMSKAPTTA 116
neosartorya_fischeri_2/1-235   52  PSDILSNDFRCNKGSMQSAASTKVYTVAPGTELGFQLAYGAEMKHPGPLQIYMSKAPGDV 111
```

```
aspergillus_fumingatus_6/1-235   52  PSEILSNDFRCNKGSMQSAANTKVYKVAPGTELGFQLAYGAEMKHPGPLQIYMSKAPGDV 111
aspergillus_tereus_12/1-235      52  AGDILSNDFRCNQGSMDSAASTKVYTVAPGTEIGFGLAYGASMKHPGPLQVYMSKAPGDV 111
aspergillus_favus_7/1-235        52  ASEIVNNDFRCNKGSMDSAAKTKVYTVAPGAEIGFQLAYGASMKHPGPLQIYMSKAPGDV 111
podospora_anseria_17/1-231       51  PDILTSDELRCNRGAKP-GGNVQTYEVKAGDKIGFKVFNNEFIEHPGPGFIYMSKAPGSV 109
chaetomium_globosum_15/1-229     49  PDIVTSNDLRCNVGAKP-GGGVQTATVKAGDKIGFKLFNNEFIEHPGPGFVYMSLAPNGV 107
glomerrela_graminic_16/1-230     51  SEIVNSPELRCNKGATT--GTAQTYTVAAGSKVGFKLWYNEFIEHPGPGFVYMSKVSGSL 108
glomerrela_graminic_31/1-230     51  SEIVNSPELRCNKGATS--ATAQTMTVAAGSKLGFKLWFNEFIEHPGPGMVYMSKAPGDL 108
Phaeospheraria_nodorum_4/1-230   51  SEIVNSPDLRCNKGAVA--GNTATYTVKAGQTIGFKLAFGEKIEHPGPGFVYIAKAPGAV 108
pyrenophora_teres_22/1-230       51  SEIVNSPELRCNKGAKP--GSTGTYTVKAGDKVGFKLSFNEKIEHPGCGFVYVSKAPSKV 108
pyrenophora_trici_repentis_23/   35  SEIVNSPELRCNKGAKP--GSTGTQTVKAGDKIGFKLAYDELIEHPGCGFVYISKAPGKV 92
podospora_anseria_5/1-221        49  VTSLSDNNLRCNVGGAS-GASTSVVNVKAGDSVTFYTDQA--VYHQGPISLYMSKAPGSV 105
myceliophthora_thermophilia_2/   49  VTDLTSNDLRCNVGATG--AGTDTVTVRAGDSFTFTTDTP--VYHQGPTSIYMSKAPGSA 104
thievela_terestis_7/1-223        49  VTDLTSDDLRCNVGAQG--AGTDTVTVKAGDQFTFTLDTP--VYHQGPISIYMSKAPGAA 104
sodaria_macrospora_4/1-217       47  VTDLTSNDLRCNVGASA--SGVETLSVAAGGTVTFKTDTP--VYHQGPTSIYLSKAPGSL 102
neurospora_tetrasperma_12/1-21   48  VTDLTSKDLVCNVGASA--EGVETLSVAAGSQVTFKTDTA--VYHQGPTSVYLSKADGSL 103
TYPE1:NCU00836/1-218             48  VTDLTSKDLVCNVGASA--EGVETLSVAAGSQVTFKTDTA--VYHQGPTSVYLSKADGSL 103
schizzophylum_commune_15/1-228   50  VTDVTSPDFTCYTTETQ--ATAETAEVAAGSSVSIKANGP--MYHQGVVNVYMADADGDA 105
schizzophylum_commune_16/1-228   50  VQDVTSPDFTCYTSETH--ATATTAEVAAGSSVSIKANGP--MYHAGVVNVYMASANPDA 105
serpula_lacrymans_5/1-229        49  VTDVTSTDLRCNVGGLASGATTSTYTVSAGSVVGLALDQA--IYHPGVVNIYMAKAPANV 106
4B5Q/1-217                       33  VTDVTSPEFRCYELDLQNTAGQTQTATVSAGDTVGFKANSA-IYHPGYLDVMMSPASPAA 91
emmericella_nidulans__3/1-229    50  VEDVTSLDIRCNKDASTNGNATETLPVKAGEEIGFTVRTN--IGHPGPLLAYMAKAPGDA 107
aspergillus_tereus_4/1-228       50  VTDVDSLDIRCNVDATT-GNNTSTLGVAAGSTIGFTVKTE--IGHPGPLLVYMAKAPTTA 106
verticillium_dahiae_24/1-222     48  IEDVSSPLLGCYEKVGR--PVSDVQTVAPGTRIRFPSSAP--IGHPGPVLFYLARVPDGQ 103
verticilium_albo_atrum_16/1-21   43  IEDVSSPLLSCYEKIGR--PVSAVQTVAPGTRIRFPSSVP--IGHPGPVLFYLARVPDGQ 98
verticillium_dahliae_26/1-221    48  VEDLSSTFLRCFEKPGR--PPSAVLPVTAGSTVGFTSSNS--MGHPGPVLFYMARVPDGE 103
pyrenophora_trici_repentis_20/   49  VTDVTSNAIRCYELSPG--TGSKTYTVNAGDTVGFTAATS--ISHPGTLQFYLAKVPSGK 104
Phaeospheraria_nodorum_28/1-22   47  VTDVTSNQIRCYELSPG--TGSKTYTVNAGDTVGFTAVSS--VSHPGPLQFYMAKVPSGK 102
thievela_terestis_11/1-231       49  VTDVTSDQIRCYELNPG-TPAPQIATVQAGGTVTFTVDPS--IQHPGPLQFYMAKAPSGQ 105
chaetomium_thermophilum_14/1-2   49  VTDVTSQEMTCFEKNPG-TPAPKTITVQAGNNVTFTVDSN--IGHPGPLHFYMAKVPAGQ 105
pyrenophora_trici_repentis_13/   45  VGDVTSSDIRCNQLRP---GTSGALSVAAGSSVKVSVNPN--AYHPGPFQSYLAKVPEGQ 99
myceliophthora_thermophilia_16   49  VTDVTSPEMTCYQSGV--QGAPQTVQVKAGSQFTFSVDPS--IGHPGPLQFYMAKVPSGQ 104
podospora_anseria_11/1-231       49  VTDVNSPLMTCYERDPG-VGAPNTLAVTAGSTVTFQVGSS--VGHPGPLHFYMAKVPAGK 105
chaetomium_globosum_24/1-226     49  VEDVSSSKMTCYQRDVG-TGAAKTVSVKAGGTVGFTVDTS--IGHPGPLHFYLAKVPSGQ 105
TYPE1:NCU02344/1-232             49  VADVTSESMTCYELNPG-KGAPKTLSVAAGSNYTFVVGDN--IGHPGPLHFYMAKVPEGK 105
arthrobotrys_oligospora_11/1-1   11  VTDVTSSDMRCYK-DPA-APIASTANVAAGSQVGFRVDTS--ISHPGPLLFYLAKVPSGR 66
glomerrela_graminic__6/1-230     49  TDGVSSTQIRCYEADAKDRGTVQTLPVTAGSTIGFAANSN--VGHPGPASIWMAKVPAGQ 106
pyrenophora_teres_11/1-209       45  VGDVTSSDIRCNQLRS---GTSGALSVAAGSSVKVSVNPN--AYHPGPFQSYLAKVPEGQ 99
Phaeospheraria_nodorum_18/1-21   44  VGDVTSSAIRCNQLSP---GKS-TLSVAAGSSVKVNVNPN--AYHPGPFQSYLAKMPDGQ 97
thievela_terestis_18/1-224       46  VGDVTSPQIRCFQATPS--PAPSVLNTTAGSTVTYWANPD--VYHPGPVQFYMARVPDGE 101
3EJA/1-208                       28  VGDVTSPQIRCFQATPS--PAPSVLNTTAGSTVTYWANPD--VYHPGPVQFYMARVPDGE 83
podospora_anseria_18/1-223       45  VGSVTSPQIRCFQNSVA--GASQTYNVSAGSQLTYYVNPN--AYHPGPMQFYLARVPDGQ 100
TYPE1:NCU03328/1-229             49  VDNVNSQQIRCFQSTHS--PAQSTLSVAAGTTITYGAAPS--VYHPGPMQFYLARVPDGQ 104
chaetomium_globosum_8/1-223      45  VGDVNSAQIRCFQSSSA--GAQDVYTVSAGSTVKYSANPN--IYHPGPMQFYLARVPDGQ 100
myceliophthora_thermophilia_21   45  VGDVNSEQIRCFQATPA--GAQDVYTVQAGSTVTYHANPS--IYHPGPMQFYLARVPDGQ 100

                                              130       140       150       160       170       180
                                     ....|....|....|....|....|....|....|....|....|....|....|....|
aspergillus_fumingatus_3/1-238   117 K--TYE-GDGDWFKIHEEGVCNKN-ADFTKDAWCTWDKDRIEFTIPADLPDGEYLIRPEH 172
neosartorya_fischeri_7/1-238     117 K--SYE-GDGDWFKIHEEGVCNKN-ADFTKNAWCTWDKDRIEFTIPSDLPDGEYLIRPEH 172
aspergillus_clavatus_1/1-238     117 K--AYQ-GDNEWFKIHEESVCNKN-ADFTRDAWCTWGKDRIEFTIPADLPDGEYLIRPEH 172
penicillum_chrysogenum_4/1-236   115 K--EYE-GDGEWFKIHQESVCDKN-KDFTKDAWCTWDKDRIEFTIPASLPDGEYLIRPEH 170
aspergilus_oryzae_5/1-238        117 K--TYQ-GDGDWFKIYEEGICDKN-KDVKSDAWCSYDKDRVEFTIPKDLADGEYLIRAEH 172
aspergillus_favus_3/1-238        117 K--TYQ-GDGDWFKIYEEGICDKN-KDVKSDAWCSYDKDRVEFTIPKDLADGEYLIRAEH 172
aspergillus_niger_9/1-238        117 K--QYE-GDGDWFKIHEEGICDQS-KDIKTDAWCTWDKDRIEFTVPADLPDGEYLIRPEH 172
emmericella_nidulans__6/1-238    117 N--QYE-GDGDWFKIHEEGICDTS-KDIKTDAWCTWDKDRIEFTIPADLPDGEYLIRSEH 172
verticilium_albo_atrum_17/1-21   117 K--AYE-GNGEWFKIFEES---------------------------DTPDGEYLIRPEH 145
verticillium_dahliae_25/1-211    117 K--SYE-GDGEWFKIFEES---------------------------DTPDGEYLFRPEH 145
aspergillus_clavatus_5/1-238     117 K--NYQ-GDGDWFKIFEDSVCNKN-ADFTREAWCSYNKDRVEFTIPADTPDGEYLIRPEH 172
colletotrichum_higginsianum_24   117 K--AYE-GDGDWFKVYEESVCNKG-GDFKGNAWCTWDKNTVEFTVPKDTPNGEYLIRVEH 172
glomerrela_graminic_7/1-238      117 A--AYE-GDGDWFKIYEESVCNKN-GAFTSDAWCTWDKNSIEFTIPKDTPDGEYLLRIEH 172
neosartorya_fischeri_2/1-235     112 K--SYD-GSGDWFKVHQEGLCADTSKGIVDEDWCTWGKDTASFKIPEDTPAGQYLVRVEH 168
aspergillus_fumingatus_6/1-235   112 R--SYD-GSGDWFKVHQEGLCADTSKGIKDEDWCTWGKDTASFKIPQDTPAGQYLVRVEH 168
aspergillus_tereus_12/1-235      112 K--EYD-GSGDWFKVYQEGVCSDISDGLVDTDWCTWGKDTVKFKIPSDMPPGQYLVRVEH 168
aspergillus_favus_7/1-235        112 K--TYD-GSGDWFKVYQEGVCEDISDGLKDTDWCTWGKDTASFKIPKDTPPGQYLVRVEH 168
podospora_anseria_17/1-231       110 A--TYD-GSGEWFKVYETGLCRGG--GNVDTNWCSWQKDRLEFTIPPKTPPGEYLVRIEH 164
chaetomium_globosum_15/1-229     108 A--NYD-GSGDWFKVYETGLCRGN--AASDGNWCSYNKDRLEFVIPPKTPPGEYLVRVEH 162
glomerrela_graminic_16/1-230     109 N--GYD-GSGDWFKVYETGLCGGQ--PNVDTSWCTWQKDRIEFTIPNRIPPGDYLVRVEH 163
glomerrela_graminic_31/1-230     109 N--SYD-GSGDWFKVFESGLCGSN--PSVDTDWCTWQKDRIEFTLPAATPPGDYLVRVEH 163
Phaeospheraria_nodorum_4/1-230   109 K--SYD-GSGDWVKVMESGLKNPS-TPGVDTAWDSWQKDRLEWKIQNSIPAGEYLVRVEH 164
pyrenophora_teres_22/1-230       109 N--TYD-GSGEWTKVMQSCLKNPS-TPGVDTAWENWQQDRLEWTIQKNIPAGEYLVRVEH 164
pyrenophora_trici_repentis_23/   93  N--NYD-GSGEWTKVMQSCLKNPS-TPGVDTAWESWQKDRLEWTIQRSVPAGEYLVRVEH 148
podospora_anseria_5/1-221        106 K--DYD-GSGEWFKIYDWGPTIN------GGQSSWPMRSSYQANLPRCIPNGEYLLRIQS 156
myceliophthora_thermophilia_2/   105 S--DYD-GSGGWFKIKDWGADFS------SGQATWTLASDYTATIPECIPPGDYLLRIQQ 155
thievela_terestis_7/1-223        105 S--DYD-GSGGWFKIKDWGPTFN-----ADGTATWDMAGSYTYNIPTCIPDGDYLLRIQS 156
sodaria_macrospora_4/1-217       103 S--SYD-GSGGWFKIKDFGATFP--------GGGWTLSDTYTFTIPSCIPSGDYLLRIQQ 151
neurospora_tetrasperma_12/1-21   104 S--SYD-GSGGWFKIKDWGATFP--------GGEWTLSDTYTFTIPSCIPSGDYLLRIQQ 152
TYPE1:NCU00836/1-218             104 S--DYD-GSGGWFKIKDWGATFP--------GGEWTLSDTYTFTIPSCIPSGDYLLRIQQ 152
```

```
schizzophylum_commune_15/1-228  106 A--SYDGSGEKWFKVYEIPAVTD---GGSTIEFPGTNITEITFDIPKALPSGEYLVRAEH 160
schizzophylum_commune_16/1-228  106 A--SYDGSGQKWFKVYEIPPVTN---GGSSISFPGSDIDTITFTIPKSLPSGDYLIRAEH 160
serpula_lacrymans_5/1-229       107 S--AWDGSGAVWFKVHEVTAITN---GGTSISWPSVGMSQVTFTLPASIPDGQYFVRAED 161
4B5Q/1-217                      92  NS-PEAGTGQTWFKIYEEKPQFE---NGQLVF--DTTQQEVTFTIPKSLPSGQYLLRIEQ 145
emmericella_nidulans__3/1-229   108 S--DFDGDGQVWFKIYEDGPTVT----DDGLTWPSDGATNVNFTIPSSLPDGDYLLRVEH 161
aspergillus_tereus_4/1-228      107 A--AFDGSGESWFKIYEDGPKFN----ADGLTWPTEGATQVTFDLPAALPDGDYLVRVEH 160
verticillium_dahiae_24/1-222    104 DVISWTPTGNVWFKVDQYGNTP------GMNSQFAVEMTEISTIIPASLRPGNYLLRAEH 157
verticilium_albo_atrum_16/1-21  99  DVNSWTPTGDVWFKVDQYGNTP------GVNNQFAVEMTEVFTTIPASLRPGNYLLRAEH 152
verticillium_dahliae_26/1-221   104 DVTTWDPVGDVWFKIDQHGDLG------GPYPAFETEMREISTTIPKTLPNGDYLLRAEH 157
pyrenophora_trici_repentis_20/  105 TAATWDGSGTEWFKIYSQGPSF----SGGQLTWPSNGKTEVDVTLPKSLPSGEYLLRGEH 160
Phaeospheraria_nodorum_28/1-22  103 TAATFDGSGAVWFKVYSQGATF----SGGQMTFASAGKTQVTFPLPKSLPSGEYLLRVEH 158
thievela_terestis_11/1-231      106 TAATFQGTGNVWFKIYEDGPSGL---GTSNITWPSSGKTEVSVKIPSCIAPGDYLLRVEH 162
chaetomium_thermophilum_14/1-2  106 TAATFNGKGPVWFKIYQDGPGGL---GTSSLTWPSYGKTEVSVQIPHCIQDGDYLLRVEH 162
pyrenophora_trici_repentis_13/  100 DVNTWDPTGAVWFRIYAEQPKFG-----SQLTWLS--AANYNINIPSCIAPGKYLMRNEH 152
myceliophthora_thermophilia_16  105 TAATFDGTGAVWFKIYQDGPNGL---GTDSITWPSAGKTEVSVTIPSCIEDGEYLLRVEH 161
podospora_anseria_11/1-231      106 TAKTFDGKGAVWFKIYQDGPSGL---GTGSIKWPSDGKTEVSVQIPSCIANGEYLLRVEH 162
chaetomium_globosum_24/1-226    106 TAATFDGKGAVWFKIYEDGPSGL---GTGNLKWPSDGKTQVSVKIPSCVQNGD-----TP 157
TYPE1:NCU02344/1-232            106 TAATFDGKGAVWFKIYQDGPMGL---GTGQLTWPSAGATEVSVKLPSCLESGEYLLRVEH 162
arthrobotrys_oligospora_11/1-1  67  TAANWDGDGAVWFKIWENRPTI----TSSSISWPS-GQTQVFVTIPSCIPAGEYLLRVEH 121
glomerrela_graminic__6/1-230    107 TAATWDGAGSQWFKIYHEQPKV----TSNGLEWASLNALVLSAKIPSCLASGEYLVRVEH 162
pyrenophora_teres_11/1-209      100 DVNTWDPTGAVWFRIYAEQPKFG-----SQLTWLS--AASYNINIPSCIAPGKYLMRNEH 152
Phaeospheraria_nodorum_18/1-21  98  DINTWDPTGAVWFRIYAEQPKFG-----SQLTWLA--AANYDIKIPSCIAPGKYLMRNEH 150
thievela_terestis_18/1-224      102 DINSWNGDGAVWFKVYEDHPTFG-----AQLTWPSTGKSSFAVPIPPCIKSGYYLLRAEQ 156
3EJA/1-208                      84  DINSWNGDGAVWFKVYEDHPTFG-----AQLTWPSTGKSSFAVPIPPCIKSGYYLLRAEQ 138
podospora_anseria_18/1-223      101 DVTRWDGSGAVWFKIYHEQPTFG-----QQLGWPSLNKGSFPVTIPRCIRSGYYLLRAEH 155
TYPE1:NCU03328/1-229            105 DINSWTGEGAVWFKIYHEQPTFG-----SQLTWSSNGKSSFPVKIPSCIKSGSYLLRAEH 159
chaetomium_globosum_8/1-223     101 DVKSWTGDGAVWFKVYHEQPNFG-----SQLTWPSNGKSSFDVSIPSCIKAGKYLLRAEH 155
myceliophthora_thermophilia_21  101 DVKSWTGEGAVWFKVYEEQPQFG-----AQLTWPSNGKSSFEVPIPSCIRAGNYLLRAEH 155

                                             190       200       210       220       230       240
                                    ....|....|....|....|....|....|....|....|....|....|....|....|
aspergillus_fumingatus_3/1-238  173 IGLHGAH-DGQAEFYYTCAQVKVVGGGTGTP--GPTIKFPGGYKKDDPSFNFSLWNG-YK 228
neosartorya_fischeri_7/1-238    173 IGLHGAH-DGQAEFYYTCAQVKVVGGGNGNP--GPTIKFPGGYKKDDPSFNFSLWNG-YK 228
aspergillus_clavatus_1/1-238    173 IGVHGAH-VGQAEFYNTCAQVKVVGGGNGTP--GPTVKFPGAYKKNDPSFNFSIYQG-YK 228
penicillum_chrysogenum_4/1-236  171 IGVHGAA-GGEAEFYYSCAQVKVVGGGNGTP--GPTVKFPGAYKKDDPSFNFSIWNG-YK 226
aspergilus_oryzae_5/1-238       173 IGVHGAH-AGEAEFYYECAQVKVVGGGNGTP--GPTVKFPGAYKKTDPSFTYSVWGG-YK 228
aspergillus_favus_3/1-238       173 IGVHGAH-AGEAEFYYECAQVKVVGGGNGTP--GPTVKFPGAYKKTDPSFTYSVWGG-YK 228
aspergillus_niger_9/1-238       173 IGVHGAH-DGQAEFYYECAQVKVTGGGNGNP--GPTIKFPGGYKKDDPSFNFSIWGG-MK 228
emmericella_nidulans__6/1-238   173 IGVHGAH-DGQAEFYYECAQVKVTGGGNGNP--QDTIKFPGGYQKDDPSFNFSVWGG-MK 228
verticilium_albo_atrum_17/1-21  146 IGVHGAH-VGQAEFYYTCVQVKVTGGGNGTP--GPTVKFPGAYKKDDPSFNFSIYGG-VK 201
verticillium_dahliae_25/1-211   146 IGVHGSH-AGQAEFYYTCVQVKVTGGRNGTP--GPTVKFPGAYKKDDSSFNFSIYGG-VK 201
aspergillus_clavatus_5/1-238    173 IGVHGAH-AGQAEFYYSCAQVKIVGGGKGKP--GPMIKFPGGYKKTDPSFNFSIYNG-YK 228
colletotrichum_higginsianum_24  173 IGVHGAH-VGQAEFYNSCAQVKITGGGNGTP--GPMIKFPGGYKKSDPSFNFSIYGG-YK 228
glomerrela_graminic_7/1-238     173 IGVHGAH-VGQAEFYNSCAQIKVTGGGNGTP--GPMIRFPGGYKKTDPSFNFSIYNG-YK 228
neosartorya_fischeri_2/1-235    169 IGLHRGF-LGESEFYFTCAQIEVTGSGSGSP--SPVVKIPGVYKPEDPNIHFNIWYPTPT 225
aspergillus_fumingatus_6/1-235  169 IGLHRGF-LGEAEFYFTCAQIEVTGSGSGSP--SPTVKIPGVYKPDDPNVHFNIWYPTPT 225
aspergillus_tereus_12/1-235     169 IGLHRGF-SGNSEFYFTCAQIEVTGSGTGTP--AEVAKIPGIYKPDDANIHFNIYYPTPT 225
aspergillus_favus_7/1-235       169 IGLHRGF-SGNSEFYFTCAQIEVTGSGSGVP--GPLVKIPGMYKPEDPNIHFDIYYPVPT 225
podospora_anseria_17/1-231      165 IGLHEGH-VRRAQFYITCAQLRITGPGGGNP--SPLVRIPGLYNANDPGIAYNKWTNNPA 221
chaetomium_globosum_15/1-229    163 IGLHEGF-KNRAQFYMECAQLKITGPGGGTP--GPLAKIPGIYKASDPGIAYDKWKSNPA 219
glomerrela_graminic_16/1-230    164 IGLHEAH-VGKAQFYMECAHLKITGSGGGTP--GPLVKIPGIYKASDPGIAYNKWTNTPA 220
glomerrela_graminic_31/1-230    164 IAIHEGH-VGKAQFYMECAHIKVTGNGAGTP--GPLVKIPGMYSSTDPGIAYNKWTGNPA 220
Phaeospheraria_nodorum_4/1-230  165 IAIHEGH-VGKAQFYMECFQLKIESSGTGKL--GPTAKIPGIYKATDPGIAFDKWTN-PK 220
pyrenophora_teres_22/1-230      165 IAIHEGH-VGKAQFYMECFQLNIQSSGTGKL--GPTVKIPGLYSAQDPGIAFNKWVN-PK 220
pyrenophora_trici_repentis_23/  149 IAIHEGH-VG------ECFQLNIQSSGTGKL--GPTVKIPGLYSAQDPGIAFNKWVN-PK 198
podospora_anseria_5/1-221       157 LAIHNPG--STPQFYISCAQINVSGGGNSSP--SPTVKIPGAFKATDPGYTANIYNN-LQ 211
myceliophthora_thermophilia_2/  156 LGIHNPWPAGIPQFYISCAQITVTGGGSANP--GPTVSIPGAFKETDPGYTVNIYNN-FH 212
thievela_terestis_7/1-223       157 LAIHNPWPAGIPQFYISCAQITVTGGGNGNP--GPTALIPGAFKDTDPGYTVNIYTN-FH 213
sodaria_macrospora_4/1-217      152 IGIHNPWPAGIPQFYLSCAHISVTGGGSAS---PATVSIPGAFKETDPGYTANIYSN-FN 207
neurospora_tetrasperma_12/1-21  153 IGIHNPWPAGVPQFYLSCAHISVTGGGSAS---PATVSIPGAFKETDPGYTVNIYSN-FN 208
TYPE1:NCU00836/1-218            153 IGIHNPWPAGVPQFYLSCAHISVTGGGSAS---PATVSIPGAFKETDPGYTVNIYSN-FN 208
schizzophylum_commune_15/1-228  161 IALHNAANKGGAQFYLSCAQVKVTGGGNGTP--GPLVSIPGVYDGNEPGIKLNIYSPIPA 218
schizzophylum_commune_16/1-228  161 IALHSASSFGGAQFYLSCAQVKVTGGGNGSP--GPLVSIPGVYTGNEPGILINIYYPVPA 218
serpula_lacrymans_5/1-229       162 IALHVAESYGGAQFYLSCAQINVQNGGNGTP--GPLVSIPGVYTGYEPGILINIYSPIPA 219
4B5Q/1-217                      146 IALHVASSYGGAQFYIGCAQLNVENGGNGTP--GPLVSIPGVYTGYEPGILINIYNL-PK 202
emmericella_nidulans__3/1-229   162 IALHGAGTEGGAQFYLSCGQVSVTGGGNGDP--APLVAFPGAYDPTDPGILINIYWPVPT 219
aspergillus_tereus_4/1-228      161 IGLHSANTEGGAQFYISCGQVTVTGGGDGTP--GPLVAFPGAYSPTDPGILIDIYYPVPT 218
verticillium_dahiae_24/1-222    158 IALHSY---QKPQFSLACAQLQVTGSGTDFP--ESFVSFPGAYKADDAGLLFDIYTSTTK 212
verticilium_albo_atrum_16/1-21  153 IALHSY---QKPQFSLACAQLQVTGSGPDFP--ESFVSFPGAYKADDVGLLFDIYAGTNN 207
verticillium_dahliae_26/1-221   158 IGLHAY---GTPQFYIACAQVEVSGGGNGTP--GPLVAFPGEYSKEDPGLAVNIYAAS-Q 211
pyrenophora_trici_repentis_20/  161 IALHSAGSAGGAQFYLSCAQLKVENGGNGSP--GPKVAFPGAYKATDPGIMINIYYPVPT 218
Phaeospheraria_nodorum_28/1-22  159 IALHSAGTSGGAQFYISCAQIKVENGGNGSP--TDLVAFPGAYKATDPGILINVYYPVPT 216
thievela_terestis_11/1-231      163 IALHSASTVGGAQFYLACAQLTVTGGTGTLN-TGELVAFPGAYSATDPGILFQLYWPIPT 221
chaetomium_thermophilum_14/1-2  163 IALHSASSIGGAQLYIACAQLTVTGGTGTLN-TGQLVSFPGAYKATDPGILFQLYWPPPT 221
pyrenophora_trici_repentis_13/  153 IAIHTAGTRGGAQFYLSCAQIEVTGGGSKTP--TNLVAFPGAYSATDPGILININYPIPT 210
myceliophthora_thermophilia_16  162 IALHSASSVGGAQFYIACAQLSVTGGSGTLN-TGSLVSLPGAYKATDPGILFQLYWPIPT 220
podospora_anseria_11/1-231      163 IALHSAGSVGGAQLYLSCAQISVTGGSGTLN-TGQLVSFPGAYKATDPGILFQLYWPTPT 221
```

```
chaetomium_globosum_24/1-226    158 PPKHLTNILPLTELYISCAQLSVTGGSGTIN-TGSLVSFPGAYKATDPGILFQLYWPIPT 216
TYPE1:NCU02344/1-232            163 IGLHSAGSVGGAQLYIACAQLNVTGGTGTINTSGKLVSFPGAYKATDPGLLFNLYYPAPT 222
arthrobotrys_oligospora_11/1-1  122 IALHSAGSAGGAQLYLACGQINVTGGSKAG--TPLVSFPGAYKATDPGLLINIYWPIPT 179
glomerrela_graminic__6/1-230    163 LALHAAGDTGGAQFYLSCAQVSLSGGSTTP--SGLVSFPGAYKATDPGILFKLYWPIPT 220
pyrenophora_teres_11/1-209      153 IALHTAGSRGGAQFYL-----------SKAP--TNLVAFPGAYSATDPGILININYPIPT 199
Phaeospheraria_nodorum_18/1-21  151 IAIHNAGTKGGAQFYLSCAQLEVTGGSTAP--KNLVAFPGAYSATDPGILININYPIPT 208
thievela_terestis_18/1-224      157 IGLHVAQSVGGAQFYISCAQLSVTGGSTEP--PNKVAFPGAYSATDPGILINIYYPVPT 214
3EJA/1-208                      139 IGLHVAQSVGGAQFYISCAQLSVTGGSTEP--PNKVAFPGAYSATDPGILINIYYPVPT 196
podospora_anseria_18/1-223      156 IALHSASSPGGAQFYISCAQIGVTGGNTEP--SNKVSFPGAYSASDPGIQININWPIPT 213
TYPE1:NCU03328/1-229            160 IGLHVAQSSGAAQFYISCAQLSITGGSTEPGANYKVSFPGAYKASDPGILININYPVPT 219
chaetomium_globosum_8/1-223     156 IGLHVAQSSGGAQFYISCAQIEVTGGSTEP--SNKVSFPGAYKASDPGILININWPIPT 213
myceliophthora_thermophilia_21  156 IALHVAQSQGGAQFYISCAQLQVTGGSTEP--SQKVSFPGAYKSTDPGILININYPVPT 213
```

```
                                             250
                                    ....|....|....|
aspergillus_fumingatus_3/1-238  229 DYP---MPGPTVW-- 238
neosartorya_fischeri_7/1-238    229 DYP---MPGPAVW-- 238
aspergillus_clavatus_1/1-238    229 EYP---MPGPAVW-- 238
penicillum_chrysogenum_4/1-236  227 EYP---MPGPEVW-- 236
aspergilus_oryzae_5/1-238       229 DYP---MPGPQVW-- 238
aspergillus_favus_3/1-238       229 DYP---MPGPQVW-- 238
aspergillus_niger_9/1-238       229 DYP---MPGPAVW-- 238
emmericella_nidulans__6/1-238   229 DYP---MPGPAVY-- 238
verticilium_albo_atrum_17/1-21  202 DYP---MPGPAVW-- 211
verticillium_dahliae_25/1-211   202 DYP---MPGPAVW-- 211
aspergillus_clavatus_5/1-238    229 PYP---MPGPKVW-- 238
colletotrichum_higginsianum_24  229 AYP---MPGPAVW-- 238
glomerrela_graminic_7/1-238     229 DYP---MPGPAVW-- 238
neosartorya_fischeri_2/1-235    226 AYN---LPGPSVW-- 235
aspergillus_fumingatus_6/1-235  226 AYS---LPGPSVW-- 235
aspergillus_tereus_12/1-235     226 AYD---LPGPSVW-- 235
aspergillus_favus_7/1-235       226 SYD---LPGPSVW-- 235
podospora_anseria_17/1-231      222 AYR---MPGPAVW-- 231
chaetomium_globosum_15/1-229    220 PYQ---MPGPAVW-- 229
glomerrela_graminic_16/1-230    221 QYI---MPGPKVW-- 230
glomerrela_graminic_31/1-230    221 PYV---IPGPAVW-- 230
Phaeospheraria_nodorum_4/1-230  221 SYT---MPGPAKY-- 230
pyrenophora_teres_22/1-230      221 SYV---VPGPALW-- 230
pyrenophora_trici_repentis_23/  199 SYV---VPGPPLW-- 208
podospora_anseria_5/1-221       212 SYT---VPGPKVF-- 221
myceliophthora_thermophilia_2/  213 NYT---VPGPAVF-- 222
thievela_terestis_7/1-223       214 NYT---VPGPEVF-- 223
sodaria_macrospora_4/1-217      208 SYT---VPGPAEF-- 217
neurospora_tetrasperma_12/1-21  209 NYT---VPGPEVF-- 218
TYPE1:NCU00836/1-218            209 NYT---VPGPEVF-- 218
schizzophylum_commune_15/1-228  219 NYS---QPGPAVW-- 228
schizzophylum_commune_16/1-228  219 NYT---MPGPAVW-- 228
serpula_lacrymans_5/1-229       220 NYT---QPGPAVW-- 229
4B5Q/1-217                      203 NFTGYPAPGPAVWQG 217
emmericella_nidulans__3/1-229   220 NYT---PPGPKVW-- 229
aspergillus_tereus_4/1-228      219 EYT---PPGPAVW-- 228
verticillium_dahiae_24/1-222    213 SYP---YPGPASP-- 222
verticilium_albo_atrum_16/1-21  208 PYP---YPGPAVW-- 217
verticillium_dahliae_26/1-221   212 RYE---YPGPEVW-- 221
pyrenophora_trici_repentis_20/  219 SYT---APGPAVW-- 228
Phaeospheraria_nodorum_28/1-22  217 SYT---PPGPKVW-- 226
thievela_terestis_11/1-231      222 SYT---NPGPAPV-- 231
chaetomium_thermophilum_14/1-2  222 SYI---NPGPAPV-- 231
pyrenophora_trici_repentis_13/  211 SYK---NPGPPTF-- 220
myceliophthora_thermophilia_16  221 EYI---NPGPAPV-- 230
podospora_anseria_11/1-231      222 SYT---NPGPAPV-- 231
chaetomium_globosum_24/1-226    217 SYV---NPGPPVV-- 226
TYPE1:NCU02344/1-232            223 SYT---NPGPAVA-- 232
arthrobotrys_oligospora_11/1-1  180 SYT---NPGPKIF-- 189
glomerrela_graminic__6/1-230    221 SYT---NPGPAPF-- 230
pyrenophora_teres_11/1-209      200 SYK---NPGPATF-- 209
Phaeospheraria_nodorum_18/1-21  209 SYK---NPGPDTF-- 218
thievela_terestis_18/1-224      215 SYQ---NPGPAVF-- 224
3EJA/1-208                      197 SYQ---NPGPAVFSC 208
podospora_anseria_18/1-223      214 SYR---NPGPPVF-- 223
TYPE1:NCU03328/1-229            220 SYK---NPGPSVF-- 229
chaetomium_globosum_8/1-223     214 SYQ---NPGPPVF-- 223
myceliophthora_thermophilia_21  214 SYQ---NPGPAVF-- 223
```
