# Supplementary material for: Bioinformatic characterization of type-specific sequence and structural features in auxiliary activity family 9 proteins
Source: Biotechnol Biofuels. 2016 Nov 9;9:239. doi: 10.1186/s13068-016-0655-2 (PMC5101804; doi:10.1186/s13068-016-0655-2)
Supplement: Supplementary file 4 — Additional file 4. Multiple sequence alignment of the Type 2 PMO sequences used in this study. [file 13068_2016_655_MOESM4_ESM.pdf]

```
                                          10        20        30        40        50        60
                                 ....|....|....|....|....|....|....|....|....|....|....|....|
chaetomium_globosum_23/1-244   1 MKLSL--VSLLGAALSVEGHAIFQKVSVNGADQGSLTGLRAPNNNNPVQDVSSQNMICGQ 58
myceliophthora_thermophilia_5/ 1 MKLSL--FSVLATALTVEGHAIFQKVSVNGADQGSLTGLRAPNNNNPVQNVNSQDMICGQ 58
thievela_terestis_2/1-244      1 MKFSL--VSLLAYGLSVEAHSIFQRVSVNGQDQGLLTGLRAPSNNNPVQDVNSQNMICGQ 58
chaetomium_thermophilum_15/1-2 1 MKLSL--ASLLTAALSVQGHAIFQKVSINGQDHGQLTGLRAPNTNYPVEDVNSQDIICGQ 58
podospora_anseria_29/1-244     1 MKFSF--IALLACGLTVDAHAIFQKISVNGQDKGQLTGIRAPNNNNPVQNVNDQNMACGQ 58
sodaria_macrospora_16/1-245    1 MKISL--LSLITYGLAVDAHAIFQKLSVNGKDNGQLTGIRAPGNNNPVQDVSSSNMACGA 58
Paravalsa_indica_13/1-241      1 MKWAA--STLLFAA-AANAHTIFQKVFVNGADQGLLVGLRAPSNNNPIQSTSDSGLACGA 57
Paravalsa_indica_12/1-241      1 MKWAA--STLLFAA-AANAHTIFQKVFVNGADQGLLVGLRAPSNNNPIQSTSDSGLACGA 57
glomerrela_graminic_17/1-244   1 MKLAF--LPAIAFASMAHAHAIFQKVSVNGKEYGSLAGLRAPNQNNPTQDVTSQDIICGK 58
verticillium_dahliae_23/1-243  1 MKYA---ISALALAALSQGHAIFQVVSVNGVEAPPLSGLRAPGINNPVEDVSSNDLTCGL 57
verticilium_albo_atrum_15/1-21 1 MR---------NLGAIWEYGSSSEVGSAPISEGPGSERTPFPRRKAPVQKAPNH------ 45
myceliophthora_thermophilia_6/ 1 MKPF----SLVALATAVSGHAIFQRVSVNGQDQGQLKGVRAPSSNSPIQNVNDANMACNA 56
chaetomium_globosum_31/1-241   1 MKAF----SLVTLATAVSGHAIFQRLSVNGQDQGQLKGIRAPYSNYPIQNVNDANIACNA 56
podospora_anseria_4/1-241      1 MKAF----TLVSLAASVSAHSIFQKVSVNGVDQGQLKGVRAPYSNFPIENVNHPDFACNT 56
sodaria_macrospora_2/1-240     1 MKTG---AILTALVASASAHTIFQKVSVNGADQGQLKGIRAPPNNNPVLNVQSGDIACNV 57
TYPE2:NCU02916/1-241           1 MKTG---SILAALVASASAHTIFQKVSVNGADQGQLKGIRAPANNNPVTDVMSSDIICNA 57
neurospora_tetrasperma_16/1-24 1 MKTG---SILAALVASASAHTIFQKVSVNGADQGQLKGIRAPANNNPVTDVMSSDIICNA 57
neurospora_tetrasperma_14/1-24 1 MKTG---SILAALVASASAHTIFQKVSVNGADQGQLKGIRAPANNNPVTDVMSSDIICNA 57
leptosphaeria_maculans_15/1-17 1 M----------------------------------------------------------- 1
botryotinia_fuckeliana_2/1-234 1 MAMAK-FIVTSYPESRAVVKAILTKP------SDTSYAIRTPTYDGPINDVTTEYVACNG 53
botryotinia_fuckeliana_12/1-24 1 MKLQL-IIPFSFLISYVSAHTIFMKLQSGGTLYNTSYAIRTPTYDGPINDVTTEYVACNG 59
sclerotinia_sclerot_5/1-240    1 MKLQF-LIPSSFLLSYVSAHTIFTQLESGGTLYNTSYAIRDPTYDGPITDVTTQYVACNG 59
glarea_lozoyensis_6/1-240      1 MKTQS-YLSFAALLPFASAHTIFSQLQVGSTTYPISYGIRTPNYDGPINDVSSKDVVCNG 59
Phaeospheraria_nodorum_9/1-227 1 MKLSTTL----FFATAASAHTIFVSVNGGKV----GDGVRVPSYDGPINDVSSNDIVCNG 52
TYPE2:NCU02240/1-235           1 MKVLSLL----AAASAASAHTIFVQLEADGTTYPVSYGIRTPSYDGPITDVTSNDLACNG 56
glomerrela_graminic_9/1-232    1 MRLLNLL----AAAGFCQAHTIFVSLDADGVNSGISQGVRTPDYDGPQTDVTSQYIACNG 56
chaetomium_thermophilum_18/1-2 1 MKLLAPL----ALVGAASAHTIFVSLEVNGVNHGVGNGVRVPSYNGPIEDVTSNSIACNG 56
myceliophthora_thermophilia_10 1 MKVLAPL----ILAGAASAHTIFSSLEVGGVNQGIGQGVRVPSYNGPIEDVTSNSIACNG 56
podospora_anseria_30/1-234     1 MKFAPI-----LLASAASAHTIFSSLEVNGVNHGVGGGVRVPSYNGPIENVDSASIACNG 55
colletotrichum_higginsianum_35 1 MKVLLSL----LTASLASAHTIFSSLEVGGVNQGQGNGVRIPSYNGPIEDVTSNSLACNG 56
sodaria_macrospora_11/1-236    1 MKVLAPL----VLASAASAHTIFSSLEVGGVNQGLGQGVRVPTYNGPIEDVTSASIACNG 56
neurospora_tetrasperma_1/1-236 1 MKVLAPL----VLASAASAHTIFSSLEVNGVNQGLGEGVRVPTYNGPIEDVTSASIACNG 56
4EIR/1-223                     1 -------------------HTIFSSLEVNGVNQGLGEGVRVPTYNGPIEDVTSASIACNG 41
neurospora_crassa_1/1-236      1 MKVLAPL----VLASAASAHTIFSSLEVNGVNQGLGEGVRVPTYNGPIEDVTSASIACNG 56
TYPE2:NCU01050/1-226           1 MKVLAPL----VLASAASAHTIFSSLEVNGVNQGLGEGVRVPTYNGPIEDVTSASIACNG 56
pyrenophora_trici_repentis_22/ 1 MKTQSVLLAALASAPAALAHTVFTDFFVDGMPQG-------------------DGVAMHV 41
Paravalsa_indica_8/1-210       1 MRLAIALVLGATYVTGVFGHATFQQLWVNGVDQGS-YCVRLPQNNNPVTSVTSNDIACNA 59
leptosphaeria_maculans_11/1-22 1 MKAGLILL---TAAASVSAHSTWQDLWVGSEDKAT-SCTRTVANNNPISSLTSADMFCGI 56
Phaeospheraria_nodorum_14/1-22 1 MKSGLVLL---ATVASVSAHSTWQQLWVGSDDKAG-TCVRTVKDNSPIASLTSTDMFCGR 56
pyrenochaeta_lyocope_1/1-227   1 MKSAIVLL---AAAASVSAHSTWQQLWVGSSDKAA-TCVRTVKDNSPIESLTSADMFCGR 56
pyrenophora_trici_repentis_11/ 1 MKYATIFL---AAAATVSAHSTWQQLWVGSEDKAG-TCVRTVKDNSPITSITSSDMFCGR 56
pyrenophora_terestis_25/1-227  1 MKYATIFL---ATAATVSAHSTWQELWVGTSDKAG-TCVRTVKDNSPITSLTSPDMFCGR 56
glarea_lozoyensis_1/1-214      1 MGNASQSL-----RKQVSSHPNAQHPSIPA---------------APVTSVSSNDIRCNA 40
glomerrela_graminic_32/1-232   1 MKSIAALALTASMANMVAGHAIFQQLWVNGKDEAD-SCVRMPASNSPIQDPTSDDMRCNA 59
pyrenophora_teres_17/1-233     1 MKSTFILAGVTAFATQVAAHATFQDL-------WS-TCARLPNSNSPVQSVTSNDIRCNA 52
pyrenophora_trici_repentis__8/ 1 MKNTFILVGLIALATQVVAHATFQDL-------WS-TCARLPNSNSPVQSVTSNDIRCNA 52
podospora_anseria_15/1-234     1 MKSTFFAALTALAAKEIAAHATFQQL-----WHGS-SCARLPPSNSPVTNVGGRDFVCNA 54
thievela_terestis_10/1-235     1 MRTTFAAALAAFAAQEVAGHAIFQQL-----WHGS-SCVRMPLSNSPVTNVGSRDMICNA 54
podospora_anseria_24/1-240     1 MKSFTATALAALLAQQAAAHSTFQQLWVDGTDFGS-QCARLPQSNSPITNYNSNDMRCNI 59
chaetomium_thermophilum_7/1-24 1 MKTLSLAALAALWAQKAAAHAMFQQLWVDGVDYGT-QCARVPGSNSPVTNVNSPEIRCNA 59
thievela_terestis_17/1-240     1 MKSFTIAALAALWAQEAAAHATFQDLWIDGVDYGS-QCVRLPASNSPVTNVASDDIRCNV 59
myceliophthora_thermophilia_12 1 MKSFTLTTLAAL-AGNAAAHATFQALWVDGVDYGA-QCARLPASNSPVTDVTSNAIRCNA 58
chaetomium_globosum_22/1-239   1 MKSFTVATLA-ALAGNAAAHATFQQLWVDGVDYGS-QCARVPSSNSPITDVSGTGVRCNV 58
```

```
                                           70        80        90       100       110       120
                                  ....|....|....|....|....|....|....|....|....|....|....|....|
chaetomium_globosum_23/1-244   59 PGS---TSQTVIQVKAGDRIGAWYQHVIGGAQFPNDPDNPIASSHKGPVMAYLAKVDNAA 115
myceliophthora_thermophilia_5/ 59 SGS---TSNTIIEVKAGDRIGAWYQHVIGGAQFPNDPDNPIAKSHKGPVMAYLAKVDNAA 115
thievela_terestis_2/1-244      59 SGS---KSQTVINVKAGDRIGSLWQHVIGGAQFSGDPDNPIAHSHKGPVMAYLAKVDNAA 115
chaetomium_thermophilum_15/1-2 59 SGH---RSSTILNVRAGDRIGTFWGHVIGGAQFPGDPDNPIARSHKGPIQVYLAKVDNAA 115
podospora_anseria_29/1-244     59 PGS---KSNTVVNVNAGDRIGAYFGHVIGGAQFPNDRDHPIAASHKGPVQAYLAKVDNAA 115
sodaria_macrospora_16/1-245    59 SGT---KSQTVINVAPGDRLGAFWGHVIGGPQFPNDQDNPIAKSHKGPVIAYLAKVNDNA 115
Paravalsa_indica_13/1-241      58 SGS---KSSTVINVKAGDTIGTYWQHVIGGPQGSNDPDNPIAKSHKGPIQIYLAKVDNAA 114
Paravalsa_indica_12/1-241      58 SGS---KSSTVINVKAGDTIGTYWQHVIGGPQGSNDPDNPIAKSHKGPIQVYLAKVDNAA 114
glomerrela_graminic_17/1-244   59 VAT---SSQEVISVAPGDRIGAWWQHVIGGAQFPGDPDNPIASSHKGPITAWLAKVPSAS 115
verticillium_dahliae_23/1-243  58 VTH---TSTDVIEVEAGDEIGAWYQHVLGGEQYPGDVDNPIAASHKGPITAWLAKVEDAA 114
verticilium_albo_atrum_15/1-21 45 -------------FEAGDEIGAWYQHILGGEQYPGDVDNPIAASHKGPITAWLAKVDDAA 92
myceliophthora_thermophilia_6/ 57 NIV--YHDNTIIKVPAGARVGAWWQHVIGGPQGANDPDNPIAASHKGPIQVYLAKVDNAA 114
chaetomium_globosum_31/1-241   57 NIQ--IKDNNIIKIPAGARVGAWWQHEIGGPSGPNDQDNPIAASHKGPITVYLAKVSNAA 114
podospora_anseria_4/1-241      57 NIQ--LRDNTVIKVPAGARVGAWWGHEIGGAAGPNDPDHPIAASHKGPIQVYLAKVNNAA 114
sodaria_macrospora_2/1-240     58 AS---IKDSNVLTVPAGAKVGHWWGHEFGGASGPNDLDNPIAASHKGPVIVYLAKVDNAA 114
TYPE2:NCU02916/1-241           58 VT---MKDSNVLTVPAGAKVGHFWGHEIGGAAGPNDADNPIAASHKGPIMVYLAKVDNAA 114
neurospora_tetrasperma_16/1-24 58 VT---MKDSNVLTVPAGAKVGHWWGHEIGGAAGPNDADNPIAASHKGPIIVYLAKVDNAA 114
neurospora_tetrasperma_14/1-24 58 VT---MKDSNVLTVPAGAKVGHWWGHEIGGASGPNDADNPIAASHKGPIIVYLTKVDNAA 114
leptosphaeria_maculans_15/1-17 1  -----PLPKDILNVKAGDKLSAEWHHTLDSKPETDKAD-PIDPGHLGPIMVYLAKVDDAL 55
```

```
botryotinia_fuckeliana_2/1-234       54  GPNPTTPSSNIINVVAGSTVNAIWRHTLDSTPANDATY-VLDPSHLGPVMAYMKKVTDAT  112
botryotinia_fuckeliana_12/1-24       60  GPNPTTPSSNIINVVAGSTVNAIWRHTLDSTPANDATY-VLDPSHLGPVMAYMKKVTDAT  118
sclerotinia_sclerot_5/1-240          60  GPNPTTPSSNIINVVAGSTVKAIWRHTLTSTPSNDATY-VLDPSHLGPVMAYMKKVDDAT  118
glarea_lozoyensis_6/1-240            60  GPNPTTPSSKIIDVKAGDTVKAVWRHTLTSTPANDAIY-VIDPSHKGPTIAYLKKVTDAT  118
Phaeospheraria_nodorum_9/1-227       53  GPNPTASTSTVITLQAGSSATLTWRHTLTSGDND-----IIDPSHKGPVMAYLKKVSDAK  107
TYPE2:NCU02240/1-235                 57  GPNPTTPSDKIITVNAGSTVKAIWRHTLTSGADD-----VMDASHKGPTLAYLKKVDDAL  111
glomerrela_graminic_9/1-232          57  PPNPTKPTDKVITVTAGSTVTAIWRHTLTSGPDD-----VMDASHKGPTIAYLKKVNDAK  111
chaetomium_thermophilum_18/1-2       57  PPNPTTPTDKVITVQAGSEVTAIWRYMLNSQGSAPND--VMDSSHKGPTMAYLKKVNNAT  114
myceliophthora_thermophilia_10       57  PPNPTTPTNKVITVRAGETVTAVWRYMLSTTGSAPND--IMDSSHKGPTMAYLKKVDNAT  114
podospora_anseria_30/1-234           56  APNPTTPTSKVITVQAGQNVTAIWRYMLSTTGSAPND--IMDISHKGPTMAYLKKVNDAT  113
colletotrichum_higginsianum_35       57  PPNPTSPTSKVITVQAGQNVTAIWRYMLSTTGSGPAD--VMDSSHKGPTIAYLKKVGDAT  114
sodaria_macrospora_11/1-236          57  SPNTVGSTSKVITVQAGTNVTAIWRYMLSTTGDSPAD--VMDSTHKGPTIAYLKKVDNAA  114
neurospora_tetrasperma_1/1-236       57  SPNTVASTSKVIIVQAGTNVTAIWRYMLSTTGDSPAD--VMDSSHKGPTIAYLKKVDNAA  114
4EIR/1-223                           42  SPNTVASTSKVITVQAGTNVTAIWRYMLSTTGDSPAD--VMDSSHKGPTIAYLKKVDNAA  99
neurospora_crassa_1/1-236            57  SPNTVASTSKVITVQAGTNVTAIWRYMLSTTGDSPAD--VMDSSHKGPTIAYLKKVDNAA  114
TYPE2:NCU01050/1-226                 57  SPNTVASTSKVITVQAGTNVTAIWRYMLSTTGDSPAD--VMDSSHKGPTIAYLKKVDNAA  114
pyrenophora_trici_repentis_22/       42  GGTK--GVSRVQSVPDGALLTFEIRSWPNNP----SKE-RLDRGHKGPCAVYLKKVNNAA  94
Paravalsa_indica_8/1-210             60  GAA---SSSGLCSVKPGDKVTVEI----------------------------YMAKVSDAK  89
leptosphaeria_maculans_11/1-22       57  GPA---KSSGVCEVAAGSALTVEMHAQPGDRKCTQP---AIGGNHYGPVLIYMAKVADAK  110
Phaeospheraria_nodorum_14/1-22       57  GPA---TSSGVCEVAAGGSLTVEMHAQPGDRKCTQP---AIGGAHYGPVLVYMAKVADAK  110
pyrenochaeta_lyocope_1/1-227         57  GPA---ASSGVCEVAAGSALTVEMHAQPGDRSCSNP---AIGGNHYGPVLIYMAKVSDAK  110
pyrenophora_trici_repentis_11/       57  GPA---STSGVCEVAAGSSLTVEMHAQPNARSCSQP---AIGGNHYGPVMVYMAKVADAK  110
pyrenophora_terestis_25/1-227        57  GPA---STSGVCEVAAGDSLTVEMHSQPNARSCSQP---AIGGAHYGPVMVYMAKVADAK  110
glarea_lozoyensis_1/1-214            41  GTK---PVTPLCTVPAGGTITVEMHAQPGDRVCTKE---AIGGNHFGPVMIYMSKVASAA  94
glomerrela_graminic_32/1-232         60  NAA---KAASTCAVKAGDVVTVEMHQHNDRD-CTKE---AIGGAHHGPVMVYMSSVADAA  112
pyrenophora_teres_17/1-233           53  NQG---PAASKCSVPAGGTVTIEMHQQNGDRSCANE---AIGGAHYGPVLVYMSKVADAS  106
pyrenophora_trici_repentis__8/       53  NQG---PAASKCSVPAGGTVTIEMHQQNGDRSCSNE---AIGGAHYGPVLVYMSKVADAS  106
podospora_anseria_15/1-234           55  GTR---GVAGKCPVRAGGTVTVEMHQQTGDRSCSQE---AIGGAHYGPVQVYLSKVPDAS  108
thievela_terestis_10/1-235           55  GTR---PVSGKCPVKAGGTVTVEMHQQPGDRSCNNE---AIGGAHWGPVQVYLSKVEDAS  108
podospora_anseria_24/1-240           60  IGT---RPQVKCPVRAGGTVTVEMHAQNGDRSCSQE---AIGGAHHGPVSVYLTKVSDAL  113
chaetomium_thermophilum_7/1-24       60  YPS---PAKGKCPVKAGSIVTIEMHAQPGDRDCSKE---AMGGAHHGPVLVYMTKVSDAA  113
thievela_terestis_17/1-240           60  GTS---RPTVKCPVKAGSTVTIEMHQQPGDRSCANE---AIGGDHYGPVMVYMSKVDDAV  113
myceliophthora_thermophilia_12       59  NPS---PARGKCPVKAGSTVTVEMHQQPGDRSCSSE---AIGGAHYGPVMVYMSKVSDAA  112
chaetomium_globosum_22/1-239         59  GTS---RPSGKCPVKAGSTVTVEMHQQNGDRSCNNE---AIGGAHYGPVIVYMSKVSDAA  112

                                                   130       140       150       160       170       180
                                         ....|....|....|....|....|....|....|....|....|....|....|....|
chaetomium_globosum_23/1-244        116  S-ASLNGLKWFKIWEDTFDTGS-------RVWGVDNLLKNNGWVYFNLPQCVASGQYLLR  167
myceliophthora_thermophilia_5/      116  T-ASKTGLKWFKIWEDTFNPST-------KTWGVDNLINNNGWVYFNLPQCIADGNYLLR  167
thievela_terestis_2/1-244           116  S-ASQTGLKWFKIWQDGFDTSS-------KTWGVDNLIKNNGWVYFHLPQCLAPGQYLLR  167
chaetomium_thermophilum_15/1-2      116  S-ASHTGLRWFKIWSEGFDVGS-------KRWGVDTMINNGGWYYFNLPQCIADGQYLMR  167
podospora_anseria_29/1-244          116  T-ASSNGQKWFKIWHEGFDQGS-------RKWGVDTVIQNQGWTYFNIPQCIAPGQYLLR  167
sodaria_macrospora_16/1-245         116  ASASSTGQQWFKIWQDGFETSS-------RKWGVDNMLQNNGWTYFNLPNCIAPGQYLLR  168
Paravalsa_indica_13/1-241           115  S-ASSSGQRWFKVASEGLSGG---------RWAVDTMIANNGMWSFKLPSCIAPGQYLMR  164
Paravalsa_indica_12/1-241           115  S-ASSSGQKWFKVASEGLNGG---------KWAVDTMIANNGMWSFKLPSCIAPGQYLLR  164
glomerrela_graminic_17/1-244        116  G-GGVTGLQWFKVAEDTLDTGS-------GQWAVDRMIGGGGWSYFDLPRCIAPGDYLLR  167
verticillium_dahliae_23/1-243       115  T-SSHANLEWFKIAEDGFDNTR-------GIWGVDNLLQQDGWTYFGIPECIAPGDYILR  166
verticilium_albo_atrum_15/1-21      93   T-SSHANLEWFKIAEDGFDNTR-------GIWGVDNLLQQDGWTYFDVPECIAPGDYLLR  144
myceliophthora_thermophilia_6/      115  T-ASPSGLKWFKVAERGLNNG---------VWAVDELIANNGWHYFDLPSCVAPGQYLMR  164
chaetomium_globosum_31/1-241        115  S-ASASGQQWFKIAERGVNNG---------VWAVDEMISNNGWHYFDVPSCVAPGQYLMR  164
podospora_anseria_4/1-241           115  N-AGTSGLQWFKVAEQGLNNG---------VWAVDNMISNGGWHYFDMPSCVAPGHYLMR  164
sodaria_macrospora_2/1-240          115  T-TGTSGLKWFKVAEAGFSGG---------KWAVDDLIANNGWSYFDMPTCIAPGQYLMR  164
TYPE2:NCU02916/1-241                115  T-TGTSGLKWFKVAEAGLSNG---------KWAVDDLIANNGWSYFDMPTCIAPGQYLMR  164
neurospora_tetrasperma_16/1-24      115  T-TGTSGLKWFKVAEAGLSNG---------KWAVDDLIANDGWSYFNMPTCIAPGQYLMR  164
neurospora_tetrasperma_14/1-24      115  T-TGTSGLKWFKVAEAGLSNG---------KWAVDDLIANDGWSYFNMPTCIAPGQYLMR  164
leptosphaeria_maculans_15/1-17      56   T-TKVTGLKWFKIYEDGMDAN--------GEWAVTRLYNKKGLVDFAIPSCIPSGHYLLR  106
botryotinia_fuckeliana_2/1-234      113  T-DVGYGPGWFKISEQGLNVA-------TQGWATTDLINNAGVQSITIPSCIANGQYLLR  164
botryotinia_fuckeliana_12/1-24      119  T-DVGYGPGWFKISEQGLNVA-------TQGWATTDLINNAGVQSITIPSCIANGQYLLR  170
sclerotinia_sclerot_5/1-240         119  T-DVGYGPGWFKISEQGLNVA-------TQGWATTDLINNAGVQSITIPSCIANGQYLLR  170
glarea_lozoyensis_6/1-240           119  T-DSGVGDGWFKIQEQGYNPS-------TGSWGVTDLIANAGLHSITIPSCLANGQYLLR  170
Phaeospheraria_nodorum_9/1-227      108  T-DSGVGGGWFKISEDAFDG---------SKWGVDRLIANKGVQTVKIPACIAPGQYLLR  157
TYPE2:NCU02240/1-235                112  T-DTGIGGGWFKIQEDGYNN---------GQWGTSTVITNGGFQYIDIPACIPSGQYLLR  161
glomerrela_graminic_9/1-232         112  T-DTGVGGGWYKIQEDGFSN---------GVWGTERVINNAGKHNITIPKCIANGQYLLR  161
chaetomium_thermophilum_18/1-2      115  T-DSGVGDGWFKIQEDGFDG---------TTWGTERVINGQGRHKIKIPECIEPGQYLLR  164
myceliophthora_thermophilia_10      115  T-DSGVGGGWFKIQEDGLTN---------GVWGTERVINGQGRHNIKIPECIAPGQYLLR  164
podospora_anseria_30/1-234          114  T-DSGVGGGWFKIQEDGYNN---------GVWGTEKVINGQGRHSIKIPSCIAPGQYLLR  163
colletotrichum_higginsianum_35      115  T-DTGVGNGWFKIQQDGFSN---------GVWGTEKVINGQGRHSIKIPECIAPGQYLLR  164
sodaria_macrospora_11/1-236         115  T-DSGVGNGWFKIQQDGMDAN--------GVWGTERVINGKGRQSIKIPECIAPGQYLLR  165
neurospora_tetrasperma_1/1-236      115  T-ASGVGNGWFKIQQDGMDSS--------GVWGTERVINGKGRHSIKIPECIAPGQYLLR  165
4EIR/1-223                          100  T-ASGVGNGWFKIQQDGMDSS--------GVWGTERVINGKGRHSIKIPECIAPGQYLLR  150
neurospora_crassa_1/1-236           115  T-ASGVGNGWFKIQQDGMDSS--------GVWGTERVINGKGRHSIKIPECIAPGQYLLR  165
TYPE2:NCU01050/1-226                115  T-ASGVGNGWFKIQQDGMDSS--------GVWGTERVINGKGRHSIKIPECIAPGQYLLR  165
pyrenophora_trici_repentis_22/      95   T-DTAAGDGWFKIFDHGYNSAT-------DRWCTDEIIDNNGLLSVNLPKGLKGGDYLAR  146
Paravalsa_indica_8/1-210            90   T-AVGSSAGWFKVSEMGLASNN------PNYYGSQVLNDNCGHYTFTVPNV-APGNYLLR  141
leptosphaeria_maculans_11/1-22      111  T---ATSGSFFKVAEDGYTGTT-------ASWGTEILNANCGKRAFTVPKNIASGDYLVR  160
Phaeospheraria_nodorum_14/1-22      111  T---ATSGSFFKVAEDGYSGTT-------ASWGTEILNANCGKRAFTVPKNIASGDYLVR  160
pyrenochaeta_lyocope_1/1-227        111  T---ATSGSFFKVAEDGHAGTV-------ATWGTEILNANCGKRAFTVPKSLASGDYLVR  160
```

```
pyrenophora_trici_repentis_11/   111 T---ATSGSFFKVAEDGYKGTT-------ATWGTEILNANCGKRAFTVPKNIASGDYLVR 160
pyrenophora_terestis_25/1-227    111 T---ATSGSFFKVAEDGYKGTT-------ATWGTEILNANCGKRAFTVPKNIASGDYLVR 160
glarea_lozoyensis_1/1-214        95  S--DPGSGSWFKISEEGYNPST-------KKWGTDSLNANCGKRTATIPSSLAPGDYLVR 145
glomerrela_graminic_32/1-232     113 A--ADGSEPFFKIFESGYFANN-------NTWGNDLINEGCGKQNVVIPADIAPGDYLLR 163
pyrenophora_teres_17/1-233       107 T--ADGSAPFFKIFQDTWAKNTGGGGGSDDYWGTKDLNKNCGKMDVKIPANLASGDYLLR 164
pyrenophora_trici_repentis__8/   107 T--AEGSAPFFKIFQDTWAKNTGGGGGSDDYWGTKDLNKNCGKMDVKIPANLASGDYLLR 164
podospora_anseria_15/1-234       109 T-ADGTSTGWFKILSNSWSKKAGGRVGDDDNWGSKDLNACCGKMDVKIPADIPSGDYLLR 167
thievela_terestis_10/1-235       109 T--ADGSTGWFKIFADTWSKKAGSSVGDDDNWGTRDLNACCGKMQVKIPADIPSGDYLLR 166
podospora_anseria_24/1-240       114 T--ADGSTGWFKIFDDGWRKNPSGRVGDDDFWGTKDLNACCGKMNVKIPSDIPSGDYLLR 171
chaetomium_thermophilum_7/1-24   114 T--ADGSTGWFKIFEDGWAKNPSGKVGDDDFWGTKDLNTCCGKMDVKIPEDIPSGDYLLR 171
thievela_terestis_17/1-240       114 T--ADGSSGWFKVFQDSWAKNPSGSTGDDDYWGTKDLNSCCGKMNVKIPEDIEPGDYLLR 171
myceliophthora_thermophilia_12   113 S--ADGSSGWFKVFEDGWAKNPSGGSGDDDYWGTKDLNSCCGKMNVKIPADLPSGDYLLR 170
chaetomium_globosum_22/1-239     113 S--ADGSSGWFKVFQDGWAKKSGGGSGDDDYWGVKDLNACCGKMDVKIPSDIAPGDYLLR 170

                                              190       200       210       220       230       240
                                     ....|....|....|....|....|....|....|....|....|....|....|.....|
chaetomium_globosum_23/1-244     168 VEVLALHSAGKQGQAQFYQSCAQINVSGSGSLSP--SDTVSFPGAYSASDPGILTSIYGS 225
myceliophthora_thermophilia_5/   168 VEVLALHSAYSQGQAQFYQSCAQINVSGGGSFTP--ASTVSFPGAYSASDPGILINIYGA 225
thievela_terestis_2/1-244        168 VEVLALHSAYQQGQAQFYQSCAQINVSGSGSFSP--SQTVSIPGVYSATDPSILINIYGS 225
chaetomium_thermophilum_15/1-2   168 VELLALHSAGNYRGAQFYQSCAQINVSGGGSFTP--SQTVSFPGAYSQNDPGILVNIYGL 225
podospora_anseria_29/1-244       168 VELLALHSANQQGQAQFYKSCAQINISGSGSFTP--SQTVSFPGAYQANHPGILTSIYGL 225
sodaria_macrospora_16/1-245      169 VEIIALHSAKNSGQAQFYQSCAQINVSGSGSYTP--SSTVSFPGAYKSNDPGILINIYGK 226
Paravalsa_indica_13/1-241        165 GELLALHSAGSVNGEQFYISCAQINVTSGGSFSP--SSTVSFPGAYSSSDPGILISIYGS 222
Paravalsa_indica_12/1-241        165 GELLALHSAYSQNGEQFYISCAQINVTSGGSFSP--SSTVSFPGAYSPTDPGILINIYGS 222
glomerrela_graminic_17/1-244     168 VELLALHSAYSQKGAQFYISCANVRVTGSGTFSP--SSTVSFPGAYQQSDPSIVINIYGA 225
verticillium_dahliae_23/1-243    167 VELLALHSAYVPRGSQFYTSCVNLRVTRGGNFAP--EETFAIPGVYEANDPAITIMIYGN 224
verticilium_albo_atrum_15/1-21   145 VELLALHSAYVPKGSQFYTSCVNLRVTRGGNFAP--AETFAIPGVYEANDPAITIMIYGN 202
myceliophthora_thermophilia_6/   165 VELLALHSASSPGGAQFYMGCAQIEVTGSGTNSG--SDFVSFPGAYSANDPGILLSIYDS 222
chaetomium_globosum_31/1-241     165 VELLALHSASTAGGAQFYMECAQVEITGSGTNSG--SNFVSFPGAYPANHPGIVVSIYDS 222
podospora_anseria_4/1-241        165 VELLALHSASVRGAAQFYMECAQIEITGSGTNTG--SNFVSFPGAYTADHPGILVSIYDL 222
sodaria_macrospora_2/1-240       165 AEIIALHNAGSSQGAQFYIGCAQINVTGGGNAS---PSTVSFPGAYSASDPGILINIYGT 221
TYPE2:NCU02916/1-241             165 AELIALHNAGSQAGAQFYIGCAQINVTGGGSASP--SNTVSFPGAYSASDPGILINIYGG 222
neurospora_tetrasperma_16/1-24   165 AELIALHNAGSQAGAQFYIGCAQINVTGGGSASP--SNTVSFPGAYSASDPGILINIYGG 222
neurospora_tetrasperma_14/1-24   165 AEIIALHNAGSQAGAQFYIGCAQINVTGGGSTSP--SNTVSFPGAYSASDPGILINIYGG 222
leptosphaeria_maculans_15/1-17   107 AEIIALHGAGSYPGAQFYMECAQINVTGGGNAN---PPTISFPGAYKGSDPGIKFQLYWP 163
botryotinia_fuckeliana_2/1-234   165 AELIALHSAGGSPGAQLYMECAQINVSGGTGTST--PSTVSFPGAYGQSDPGILINIYQT 222
botryotinia_fuckeliana_12/1-24   171 AELIALHSAGGSQGAQLYMECAQINVSGGTGTST--PSTVSFPGAYGQSNPGILINIYQT 228
sclerotinia_sclerot_5/1-240      171 AELIALHAASGLQGAQLYMECAQINVSGGTGTSS--PSTVSFPGAYAQNDPGILINIYQT 228
glarea_lozoyensis_6/1-240        171 AELIALHSAGSSQGAQLYMECAQINVTGGSGTAA--PSTVAFPGAYKSTDPGILINIYQT 228
Phaeospheraria_nodorum_9/1-227   158 GELIALHSASSSMGAQFYMECAQINIVGGSADKT--PATVSFPGAYKQNDVGIIYNLYSG 215
TYPE2:NCU02240/1-235             162 AEMIALHAASSTAGAQLYMECAQINIVGGTGGTALPSTTYSIPGIYKATDPGLLVNIYSM 221
glomerrela_graminic_9/1-232      162 AEMIALHSASSYPGAQLYMECAQINVVGGTAAKT--PSTVSFPGAYKGTDPGITLSIYYP 219
chaetomium_thermophilum_18/1-2   165 AEMLALHGASNYPGAQFYMECAQLNIVGGTGTKK--PSTVSFPGAYSGNDPGVKINIWWP 222
myceliophthora_thermophilia_10   165 AEMLALHGASNYPGAQFYMECAQLNIVGGTGSKT--PSTVSFPGAYKGTDPGVKINIYWP 222
podospora_anseria_30/1-234       164 AEMLALHGAGNYPGAQFYMECAQLNIVGGTGSKT--PSTVAFPGAYSGSHPGVKISIYWP 221
colletotrichum_higginsianum_35   165 AEMIALHGAGSYPGAQFYMECAQINVVGGTGTKT--PSTVSFPGAYKSSDPGVTISIWWP 222
sodaria_macrospora_11/1-236      166 AEMIALHSAGNYPGAQFYMECAQLNVVGGTGAKT--PSTVSFPGAYSGSDPGVKINIYWP 223
neurospora_tetrasperma_1/1-236   166 AEMIALHAASNYPGAQFYMECAQLNVVGGTGAKT--PSTVSFPGAYSGSDPGVKISIYWP 223
4EIR/1-223                       151 AEMIALHAASNYPGAQFYMECAQLNVVGGTGAKT--PSTVSFPGAYSGSDPGVKISIYWP 208
neurospora_crassa_1/1-236        166 AEMIALHAASNYPGAQFYMECAQLNVVGGTGAKT--PSTVSFPGAYSGSDPGVKISIYWP 223
TYPE2:NCU01050/1-226             166 AEMIALHAASNYPGAQFYMECAQLNVVGGTGAKT--PSTVSFPGAYSGSDPGVKISIYWP 223
pyrenophora_trici_repentis_22/   147 PEILALHAAK-DGDPQEYTGCAQIFLQSSGNLVP--ESTVSIPGIMKYNTPPTDFDIYNT 203
Paravalsa_indica_8/1-210         142 AEVIALHVASSVGGAQFYMTCFQLSVGGSGTGS---PSTVKLPGAYGASDPGILYTLWMS 198
leptosphaeria_maculans_11/1-22   161 AEAIALHAG--AGNPQPYVSCFQVKVTGGGSAN---PTGVSFPGGYKISDPLFTKSIYDS 215
Phaeospheraria_nodorum_14/1-22   161 AEALALHAG--VGQPQPYVSCFQVKVTGGGSAN---PAGVSFPGAYKLSDGLFTKAIYDS 215
pyrenochaeta_lyocope_1/1-227     161 AEVLALHAG--AGNAQPYVSCFQVKVTNGGSAS---PSGVSFPGAYKNSDGLFTKSIWDS 215
pyrenophora_trici_repentis_11/   161 AEVIALHAG--AGQSQPYVSCFQVKVTGGGSAT---PAGVSFPGGYKTSDSLFTQSIYNS 215
pyrenophora_terestis_25/1-227    161 AEAIALHAG--ANQPQPYVSCFQVKVTGGGSAT---PAGVSFPGGYKTSDSLFTQSIYNS 215
glarea_lozoyensis_1/1-214        146 AEAIALHSASSAGGAQFYMTCYQVTVTGTGTAN---PAGVKFPGAYKANDPGIQINIYNN 202
glomerrela_graminic_32/1-232     164 AETVALHAAGSVDGAQYYMSCYQLKVEGSGSAK---PTGVTFPGAYKATDPGLLINIYNK 220
pyrenophora_teres_17/1-233       165 AEAIALHASSGLGGAQFYMTCFQITVTGGGSSS---PAGVSFPGAYKATDPGIQINIYQN 221
pyrenophora_trici_repentis__8/   165 AEAIALHAASGLGGAQFYMTCYQITVTGGGSSS---PAGVSFPGAYKAADPGIQINIYQN 221
podospora_anseria_15/1-234       168 AESLALHAAGPSGGGQFYMTCYQITVSGGGNAS---PATVRLPGAYGASEG--QVNIHAA 222
thievela_terestis_10/1-235       167 AEALALHTAGQVGGAQFYMSCYQITVSGGGSAS---PATVKFPGAYSANDPGIHINIHAA 223
podospora_anseria_24/1-240       172 AEAIALHAAGGAGGAQPYMTCYQITVSGGGSAS---PPTVSIPGHFKASDPGVQVNIHGA 228
chaetomium_thermophilum_7/1-24   172 AEAIALHVGGQPGGAQLYMTCYQLTVEGGGNAN---PATVKFPGAYSATDPGILVNIHSA 228
thievela_terestis_17/1-240       172 AEVIALHVAASSGGAQFYMSCYQLTVTGSGSAT---PSTVNFPGAYSASDPGILINIHAP 228
myceliophthora_thermophilia_12   171 AEALALHTAGSAGGAQFYMTCYQLTVTGSGSAS---PPTVSFPGAYKATDPGILVNIHAP 227
chaetomium_globosum_22/1-239     171 AEVIALHTAGGAGGAQLYMTCYQLSVTGGGSAT---PATVSFPGAYKSSDPGILVNIHSA 227

                                              250       260
                                     ....|....|....|....|.
chaetomium_globosum_23/1-244     226 TGKPDNDGKAYTAPGPRPI-- 244
myceliophthora_thermophilia_5/   226 TGQPDNNGQPYTAPGPAPI-- 244
thievela_terestis_2/1-244        226 TGQPDNGGKAYNPPGPAPI-- 244
chaetomium_thermophilum_15/1-2   226 TGQPDNGGKPYQAPGPAPI-- 244
podospora_anseria_29/1-244       226 TGQPDNGGKPYSIPGPAPI-- 244
```

```
sodaria_macrospora_16/1-245      227 LGQPDMDGKPYTVPGPAPI-- 245
Paravalsa_indica_13/1-241        223 SGQPDNGGRAYTPPGPAVI-- 241
Paravalsa_indica_12/1-241        223 SGQPDNGGKAYTPPGPAVI-- 241
glomerrela_graminic_17/1-244     226 GGVPNNGGKAYSAPGPRPI-- 244
verticillium_dahliae_23/1-243    225 TGKPDNNLKPYTPAGPRPI-- 243
verticilium_albo_atrum_15/1-21   203 T------GEAGQQF------- 210
myceliophthora_thermophilia_6/   223 SGKPNNGGRSYPIPGPRPI-- 241
chaetomium_globosum_31/1-241     223 TGKPTMNGRTYQIPGPAPI-- 241
podospora_anseria_4/1-241        223 QGRPTNGGRPYTIPGPAPL-- 241
sodaria_macrospora_2/1-240       222 GGSTNNGGRAYQIPGPPAV-- 240
TYPE2:NCU02916/1-241             223 SGKTDNGGKPYQIPGPALF-- 241
neurospora_tetrasperma_16/1-24   223 SGTTDNGGKPYQIPGPALF-- 241
neurospora_tetrasperma_14/1-24   223 SGKTDNGGKPYQIPGPALF-- 241
leptosphaeria_maculans_15/1-17   164 K------PTSYSIPGPRPF-- 176
botryotinia_fuckeliana_2/1-234   222 -------LTTYTIPGPTPF-- 234
botryotinia_fuckeliana_12/1-24   228 -------LTTYTIPGPTPF-- 240
sclerotinia_sclerot_5/1-240      228 -------LSSYPIPGPTPF-- 240
glarea_lozoyensis_6/1-240        228 -------LPSYKIPGPPVF-- 240
Phaeospheraria_nodorum_9/1-227   215 -------QKTYTSPGPSVF-- 227
TYPE2:NCU02240/1-235             222 S-----PSSTYTIPGPAKF-- 235
glomerrela_graminic_9/1-232      220 P------VTNYVIPGPQKF-- 232
chaetomium_thermophilum_18/1-2   223 P------VTSYKVPGPPVF-- 235
myceliophthora_thermophilia_10   223 P------VTSYQIPGPGVF-- 235
podospora_anseria_30/1-234       222 P------VTNYQIPGPSVF-- 234
colletotrichum_higginsianum_35   223 P------VTNYVIPGPDVF-- 235
sodaria_macrospora_11/1-236      224 P------VTSYTVPGPSVF-- 236
neurospora_tetrasperma_1/1-236   224 P------VTSYTVPGPSVF-- 236
4EIR/1-223                       209 P------VTAYTVPGPSVFTC 223
neurospora_crassa_1/1-236        224 P------VTSYTVPGPSVF-- 236
TYPE2:NCU01050/1-226             224 P------VT------------ 226
pyrenophora_trici_repentis_22/   204 P------ASKYQIPGPPVA-- 216
Paravalsa_indica_8/1-210         198 -------SKIYTIPGPSSV-- 210
leptosphaeria_maculans_11/1-22   215 -------SFNAN----ALY-- 223
Phaeospheraria_nodorum_14/1-22   215 -------SFKYVSPGPAVY-- 227
pyrenochaeta_lyocope_1/1-227     215 -------SFKYVSPGPAVW-- 227
pyrenophora_trici_repentis_11/   215 -------DFKYVSPGPAVY-- 227
pyrenophora_terestis_25/1-227    215 -------DFKYTSPGPAVY-- 227
glarea_lozoyensis_1/1-214        202 -------LQSYTIPGPAVW-- 214
glomerrela_graminic_32/1-232     220 -------ITNYVVPGPAVY-- 232
pyrenophora_teres_17/1-233       221 -------LASYVAPGPAVI-- 233
pyrenophora_trici_repentis__8/   221 -------LASYAAPGPAVI-- 233
podospora_anseria_15/1-234       222 -------LTSYTAPGPAVY-- 234
thievela_terestis_10/1-235       223 -------VSNYVAPGPAVY-- 235
podospora_anseria_24/1-240       228 -------MTNYVIPGPAVY-- 240
chaetomium_thermophilum_7/1-24   228 -------MNNYVVPGPPVY-- 240
thievela_terestis_17/1-240       228 -------MSTYVVPGPTVY-- 240
myceliophthora_thermophilia_12   227 -------LSGYTVPGPAVY-- 239
chaetomium_globosum_22/1-239     227 -------MSGYTVPGPAVY-- 239
```
