# Supplementary material for: Bioinformatic characterization of type-specific sequence and structural features in auxiliary activity family 9 proteins
Source: Biotechnol Biofuels. 2016 Nov 9;9:239. doi: 10.1186/s13068-016-0655-2 (PMC5101804; doi:10.1186/s13068-016-0655-2)
Supplement: Supplementary file 5 — Additional file 5. Multiple sequence alignment of the Type 3 PMO sequences used in this study. [file 13068_2016_655_MOESM5_ESM.pdf]

```
                                                       10        20        30        40        50        60
                                               ....|....|....|....|....|....|....|....|....|....|....|....|
hypocrea_rufa_1/1-246               1    -------MIQKLSNLLVTALAVATGVVGHGHINDIVINGVWYQAYDPTTFPYESNPPIVV  53
hypocrea_rufa_2/1-246               1    -------MIQKLSNLLVTALAVATGVVGHGHINDIVINGVWYQAYDPTTFPYESNPPIVV  53
trichoderma_saturnusporum_1/1-      1    -------MIQKLSNLLVAALTVATGVVGHGHINNIVINGVYYQAYDPTSFPYESNPPIVV  53
hypocrea_orientalis_1/1-246         1    -------MIQKLSNLLLTALAVATGVVGHGHINNIVVNGVYYQGYDPTSFPYESDPPIVV  53
trichoderma_SP_SSL_1/1-246          1    -------MIQKLSNLLLTALAVATGVVGHGHINNIVVNGVYYQGYDPTSFPYESDPPIVV  53
Hypocrea_virens_3/1-246             1    -------MTQKLTSLLVTALTVATGVIGHGHVNNIVINGAYYQGYDPTLFPYEPNPPIVV  53
trichoderma_atroviride_2/1-246      1    -------MAQKLSNLFAIALTVATGVVGHGHVNNIVVNGVYYQGYDPTSFPYMPDPPIVV  53
chaetomium_globosum_5/1-220         1    -------MPSYTSKTLLSALAGAVSVAAHGHVTNIVINGVSYEGFDPTSFPYNPNPPTVV  53
TYPE3:NCU05969/1-243                1    -------MPSFTSKSLLAVLAGAASVAAHGHVSNIVINGEYYRG-FDSSLNYMANPPAVV  52
aspergillus_tereus_6/1-245          1    -------MDRLSKSTLLALL--ASQVAGHGHVTNIVINGVSYQGWDINSFPYMADPPTVV  51
aspergillus_kawachii_37/1-247       1    -------MRQAQSASLLAALLSATQVAAHGHVTNLVVDGVYYEGFDISVFPYESDPPKVA  53
aspergillus_tereus_10/1-245         1    -------MHTLQSAILLGGL-LATQVAAHGHVTNIVINGVYYRGWNIDSDPYNSNPPLVA  52
neosartorya_fischeri_4/1-247        1    -------MRHVQSTQLLAALLFTTHVTAHGHVTNIVVNGVSYRGWNIDSDPYNPNPPVVV  53
aspergillus_fumingatus_4/1-247      1    -------MRHVQSTQLLAALLLTTRVTAHGHVTNIVINGVSYRGWNIDSDPYNPDPPVVV  53
giberella_zeae_7/1-251              1    MA-FQSINSSKA-SFWLTLLLPALGISAHGHVDEIIVNGVSYQGYGSTDFPYMQDPPVVA  58
fusarium_oxysporum_3/1-252          1    MSLLSEMTCFKA-ACWLALLLPAITVSAHGHVDEIIINGVSYQGYGSTDFPYMENPPVVA  59
nectria_heamatococcuss_1/1-252      1    MTFFTAMSTLCA-SAWLYLLFSAVSVSAHGHVTQVIINGVAYGGYLSTSFPLQRKPPVVL  59
verticilium_albo_atrum_13/1-24      1    ----------MAQKALFAVVFGAVSAAAHGFVETITVNGKTYDNYNPSTFPYNPSPPIVP  50
verticillium_dahliae_4/1-241        1    ----------MAQKALFAVVLGAVSAAAHGFVETITVNGKTYDNYNPSTFPYNPSPPVVP  50
neurospora_tetrasperma__2/1-24      1    ----------MARKSILTALAGASLVAAHGHVSKVIVNGVEYQNYDPTSFPYNSNPPTVI  50
TYPE3:NCU07760/1-240                1    ----------MARMSILTALAGASLVAAHGHVSKVIVNGVEYQNYDPTSFPYNSNPPTVI  50
podospora_anseria_31/1-244          1    -------MSNKA-ATLLAALSGAALVAAHGHVSHIIVNGVYYQNYDPTTHFYQPNPPTVI  52
magna_porte_oryzae_16/1-243         1    ---------MKS-AALLAALGSAGTVLAHGHVDYIIVEGVQYPGYDVTKYPWQSQQPTVV  50
glomerrela_graminic_4/1-242         1    -------MSSFKNSIVLSALAGAVSVMAHGHVVSFTDGSQEFSGYDVTKMAYMNPAPEVY  53
aspergillus_niger_1/1-245           1    ---------MPLSKIAGVLLASASLVAGHGYVSSIEVDGTTYGGYLVDTYYYESDPPELI  51
aspergillus_kawachii_40/1-245       1    ---------MSLSKIAGVLLASASLVAGHGYVSSIEVDGTTYGGYLVDTYYYESDPPELI  51
aspergillus_tereus_5/1-232          1    ---------MSLSKIATGILASATLVAGHGYVSGIVADGKYYSGYLVDKYSYMNDPPETI  51
3ZUD/1-228                          1    ----------------------------HGFVQNIVIDGKNYGGYLVNQYPYMSNPPEVI  32
emmericella_nidulans_9/1-245        1    ---------MSVARTAGFALASAAIVAGHGYVTGIVADGTYYGGYLVNQYPYSNDPPAVV  51
penicillium_chrysogenum_2/1-245     1    ---------MSVSKIAGLMLSSAAMVAGHGFVSGAVVDGTYHGGYLVNNYPYNDNHPETI  51
aspergillus_fumingatus_1/1-245      1    ---------MSVPKIAAALLSSAALVAGHGFVTGAVVDGKYYTGYLVNQYPYMSSPPDSI  51
neosartorya_fischeri_1/1-245        1    ---------MSVSKIAAVLLSSAALVAGHGFVSGAVVDGKYYTGYLVNQYPYMSSPPESI  51
aspergillus_clavatus_6/1-241        1    ---------MSVTKIAGILLGSAAMVAGHGFVTGAVVDGTYHTGYLVNNYPYSSNPPKTI  51
aspergilus_oryzae_7/1-242           1    ---------MSIAKIAGVVLGSAALVAGHGYVSGAVVDGQYYSGYDM-SYHYMSNPPQVI  50
aspergillus_favus_5/1-242           1    ---------MSIAKIAGIVLGSAALVAAHGHVSGAVVDGQYYPGYDI-SYHYMPDPPKVI  50
aspergillus_tereus_8/1-241          1    ---------MSVAKIAGVVLGSAALVAGHGYVTGAVVDGKYYAGYDVTSAPYSSDPPATI  51
aspergillus_niger_2/1-245           1    ---------MSVAKIAGVVLGSAALVAGHGYVSGAVIDGEYYGGYIVSSYPYESDPPETI  51
aspergillus_niger_12/1-244          1    ---------MSVAKIAGVVLGSAALVAGHGYVSGAVIDGEYYGGYIVSSYAYESDPPETI  51
aspergillus_kawachii_38/1-245       1    ---------MSVAKIAGVVLGSAALVAGHGYVSGAVVDGQYYGGYIVSSYAYESDPPETI  51
zea_mys_1/1-245                     1    ---------MSVAKIAGVVLGSAALVAGHGYVSGAVVDGQYYGGYIVTSYAYESDPPETI  51
2VTC/1-228                          1    ----------------------------HGQVQNFTINGQYNQGFILDYYYQKQNTGHFP  32
TYPE3:NCU07898/1-239                1    ---------MKT---FATLLASIGLVAAHGFVDNATIGGQFYQFYQPYQDPYMGSPPDRI  48

                                                       70        80        90        100       110       120
                                               ....|....|....|....|....|....|....|....|....|....|....|....|
hypocrea_rufa_1/1-246               54   GWTAA---DLDNGFVSPDAYQNPDIICHKNATNAKGHASVKARDTILFQWVPV-PWPHPG  109
hypocrea_rufa_2/1-246               54   GWTAA---DLDNGFVSPDAYQNPDIICHKNATNAKGHASVKAGDTILFQWVPV-PWPHPG  109
trichoderma_saturnusporum_1/1-      54   GWTAA---DLDNGFVSPDAYGSPDIICHKNATNAKGHASVRAGDTVLFQWVPL-PWPHPG  109
hypocrea_orientalis_1/1-246         54   GWTAA---DLDNGFVSPDAYQSPDIICHKNATNAKGHASVKAGDTILFQWVPV-PWPHPG  109
trichoderma_SP_SSL_1/1-246          54   GWTAA---DLDNGFVSPDAYQSPDIICHKNATNAKGHASVKAGDTIPLQWVPV-PWPHPG  109
Hypocrea_virens_3/1-246             54   GWTAS---DTDNGFVAPDAYQSPDIICHRNATNARGHASVMAGSSVLIQWVPI-PWPHPG  109
trichoderma_atroviride_2/1-246      54   GWTAA---DTDNGFVSPDAYQTPDIVCHKNGTNAKGHASVKAGDSVLFQWVPV-PWPHKS  109
chaetomium_globosum_5/1-220         54   GWTEG---NSDNGFVAPDAFGNGDIICHKSATNAGGHAVVAAGDSVFIQWDTW-PESHHG  109
TYPE3:NCU05969/1-243                53   GWKAN---NQDNGFVGPDAFSSPDIICHKDATNAKGHAVVKAGDKISIQWETW-PESHKG  108
aspergillus_tereus_6/1-245          52   AWGTP---NTGNGFIAPDAFTSDDMICHLNATNAKGFATVAAGDSINLQWTEW-PESHHG  107
aspergillus_kawachii_37/1-247       54   AWTTP---NTGNGFISPDAYRDPNIICHENATNAQAHVVVGAGEKINIQWTAW-PDSHHG  109
aspergillus_tereus_10/1-245         53   AWRTP---NTANGFIAPDAFGTSDIICHLNALNGQGHIQVAAGDRISLQWNTW-PESHHG  108
neosartorya_fischeri_4/1-247        54   AWQTP---NTANGFISPDAYGTNDIICHLNATNARGHAVVAAGDKISIQWTTW-PDSHHG  109
aspergillus_fumingatus_4/1-247      54   AWQTP---NTANGFISPDAYGTNDIICHLNATNARGHAVVAAGDKISIQWTAW-PDSHHG  109
giberella_zeae_7/1-251              59   GWTIE---QADNGFVSPDKYDDPDIICHRDATPAKGHIELAAGDTLTLRWSGW-PENHSG  114
fusarium_oxysporum_3/1-252          60   GWTIS---QRDNGFVSPEAYGDPDIICHRDATPAEGHIEVTAGDVITLRWSGW-PENHIG  115
nectria_heamatococcuss_1/1-252      60   GWTIE---QRDNGFVSPDKYDHPDIICHRDATPAQGHVQVAAGDTITIKWSSW-PENHRG  115
verticilium_albo_atrum_13/1-24      51   GWTAD---FPDLGFVEPAATGDPDIICHRSATNGGSHIPLAAGDTLTLKWSPW-PESHKG  106
verticillium_dahliae_4/1-241        51   GWTAD---FPDLGFVEPAATGDPDIICHRSATNGGSHIPVAAGDTLTLKWSPW-PESHKG  106
neurospora_tetrasperma__2/1-24      51   GWTID---QKDNGFVSPDAFDSGDIICHKSATPAGGHATVKAGDKISLQWDQW-PESHKG  106
TYPE3:NCU07760/1-240                51   GWTID---QKDNGFVSPDAFDSGDIICHKSAKPAGGHATVKAGDKISLQWDQW-PESHKG  106
podospora_anseria_31/1-244          53   GWSAL---QQDNGFVEPNNFGTTDIICHKSAAPGGGSATVNAGDKISIVWTPEWPESHIG  109
magna_porte_oryzae_16/1-243         51   GWSAT---NTDNGFVEPNNFGSPDIICHRGAQPAKGHARVKAGDRILLQWDTW-PESHHG  106
glomerrela_graminic_4/1-242         54   GWSET---ATDNGFVAPSAYTDADIICHRGAKNAALTAKVAAGDKLTVFWDTW-PQSHVG  109
aspergillus_niger_1/1-245           52   AWSTN---ATDDGYVSPTQYDSSNIVCHRGSAPGALAAPVTPGGTVKMTWNTW-PDDHHG  107
aspergillus_kawachii_40/1-245       52   AWSTN---ATDDGYVSPTQYESSNIVCHRGSAPGALEAPVAPGGTVKMTWNTW-PDDHHG  107
aspergillus_tereus_5/1-232          52   GWSTT---ATDLGFVDGTGYDTVDIACHKGSAPGALTATVPAGSKIEMQWNTW-PESHHG  107
3ZUD/1-228                          33   AWSTT---ATDLGFVDGTGYQTPDIICHRGAKPGALTAPVSPGGTVELQWTPW-PDSHHG  88
emmericella_nidulans_9/1-245        52   GWAED---ATDLGFVDGSGYTSGDIICHKDATNAQASATVAAGGTVELQWTEW-PESHHG  107
penicillium_chrysogenum_2/1-245     52   GWAEK---ATDLGFVDGSGYSGPDIICHKEATPGAISAEVKAGGDVELQWTEW-PESHHG  107
```

```
aspergillus_fumingatus_1/1-245   52  GWSET---ATDLGFVDGSGYSSGDIICHKDAKNGAISAEIKAGGKVEFQWTEW-PESHHG 107
neosartorya_fischeri_1/1-245     52  GWSET---ATDLGFVDGSGYSSGDIICHKSAKNGAISAEIKAGGKVEFQWTEW-PESHHG 107
aspergillus_clavatus_6/1-241     52  GWSET---ATDLGFVDGTGYASGDIICHKNAKPGALSADIKAGGKVEFQWTQW-PESHHG 107
aspergilus_oryzae_7/1-242        51  GWSTD---ATDLGFVDGSSYADADIICHKNAKNGAISAEIAAGKQVELQWTAW-PESHKG 106
aspergillus_favus_5/1-242        51  GWSTD---ATDNGFVDGSSYADADIICHKNAKNGAISAEIAAGKQVELQWTAW-PESHKG 106
aspergillus_tereus_8/1-241       52  GWSTT---ATDLGFVDGTEYSEPDIICHKDAKPGSLSAEITAGGKVELQWTEW-PESHHG 107
aspergillus_niger_2/1-245        52  AWSTT---ATDLGFVDGSEYSDPDIICHKSAKPGAISADVKAGGTVELQWTDW-PSSHHG 107
aspergillus_niger_12/1-244       52  AWSTT---ATDLGFVDGSEYSDPDIICHKSAKPGAISADVKAGGTVELQWTDW-PSSHHG 107
aspergillus_kawachii_38/1-245    52  AWSTE---ATDLGFVDGSEYAEPDIICHKSAKPGAISADVKAGGTVELQWTDW-PSSHHG 107
zea_mys_1/1-245                  52  AWSTE---ATDLGFVDGSEYAEADIICHKSAKPGAISADVKAGGTVELQWTDW-PSSHHG 107
2VTC/1-228                       33  NVAGWYAEDLDLGFISPDQYTTPDIVCHKNAAPGAISATAAAGSNIVFQWGPGVWPHPYG 92
TYPE3:NCU07898/1-239             49  SRK-------IPGNGPVEDVTSLAIQCNADSAPAKLHASAAAGSTVTLRWTIW-PDSHVG 100

                                                130       140       150       160       170       180
                                     ....|....|....|....|....|....|....|....|....|....|....|....|
hypocrea_rufa_1/1-246            110 PIVDYLANCNGDCE---TVDKTTLEFFKIDGVGLLSGGD-PGTWASDVLISNNNTWVVKI 165
hypocrea_rufa_2/1-246            110 PIVDYLANCNGDCE---TVDKTTLEFFKIDGVGLLSGGD-PGTWASDVLISNNNTWVVKI 165
trichoderma_saturnusporum_1/1-   110 PIVDYLANCNGDCE---TVDKTSLEFFKIDGVGLISGGD-PGNWASDVLIANNNTWVVKI 165
hypocrea_orientalis_1/1-246      110 PIVDYLANCNGDCE---TVDKTSLEFFKIDGVGLISGGD-PGNWASDVLIANNNTWVVKI 165
trichoderma_SP_SSL_1/1-246       110 PIVDYLANCNGDCE---TVDKTSLEFFKIDGVGLISGGD-PGNWASDVLIANNNTWVVKI 165
Hypocrea_virens_3/1-246          110 PVLDYLANCNGDCE---TVDKTTLEFFKIDGIGLISGGN-PGRWASDVLIGNNGTWVVQI 165
trichoderma_atroviride_2/1-246   110 TVVDYLANCNGPCE---TVDKTTLEFFKIDGIGLLSGGN-PGTWGSDVLIGNNNTWVIQI 165
chaetomium_globosum_5/1-220      110 PVIDYLASCGDAGCE--TVDKTSLEFFKVAEAGLIDGTNAPGFWASDQLIANNNSWMVKI 167
TYPE3:NCU05969/1-243             109 PVIDYLANCGASGCE--TVDKTSLEFFKIDEVGLVDGQK----WGSDQLIANNNSWLVEI 162
aspergillus_tereus_6/1-245       108 PVLDYLAPCGTAGCE--TVDKTTLQFFKIDGVGLVDDAAPPGVWAADQLIANNNSWLVQI 165
aspergillus_kawachii_37/1-247    110 PVLDYLARCDGSCE---TVDKTDLEFFKIDGVGLVSDSEVPGTWGTDQLINNNNSWMVEI 166
aspergillus_tereus_10/1-245      109 PVLDYLADCGGSCE---TVDKTTLKFFKIDGVGLVDDTTPPGIWGDDQLIANNNTWLVEI 165
neosartorya_fischeri_4/1-247     110 PVIDYLARCGSSCE---TVDKTTLEFFKIDGVGLVDGSTPPGVWGDDQLIADNNSWLVEI 166
aspergillus_fumingatus_4/1-247   110 PVIDYLARCGSSCE---TVDKTTLEFFKIDGVGLVDGSNPPGVWGDDQLIADNNSWLVEI 166
giberella_zeae_7/1-251           115 PILNYLANCNGPCE---RVDKTKLEFFKIDGLGLLEQGT-PGRYADKVLQDNGDRWNVRI 170
fusarium_oxysporum_3/1-252       116 PVLNYLANCKGPCE---RVDKTELEFFKIDGLGQLEQGT-PGRYADSVLQENGDRWNVRI 171
nectria_heamatococcuss_1/1-252   116 PVMDYLANCNGPCE---TVDKTKLEFFKIDGMGLISQDR-PGKYADGALRENGYTWSVRI 171
verticilium_albo_atrum_13/1-24   107 PIIDYLANCNGDCT---TVDKTALRFFKIAEQGLLDAA--NSNWAADELIAAGEVWEVTI 161
verticillium_dahliae_4/1-241     107 PIIDYLANCNGDCT---TVDKTALRFFKIAEQGLLDAA--SSNWAADELIAAGEVWEVTI 161
neurospora_tetrasperma__2/1-24   107 PVIDYLAACDGDCE---SVDKTALKFFKIDGAGYDATN----GWASDVLIKDGNSWVVEI 159
TYPE3:NCU07760/1-240             107 PVIDYLAACDGDCE---SVDKTALKFFKIDGAGYDATN----GWASDTLIKDGNSWVVEI 159
podospora_anseria_31/1-244       110 PVIDYLANCNGPCE---TVDKTSLRWFKIGGAGYNPNT---RTWAADDLRANGNSWLVQI 163
magna_porte_oryzae_16/1-243      107 PVIDYLARCSGNCE---TVDKTSLRFFKIGAGTYISGSP-PGYWASDELIKNGFSWVVQI 162
glomerrela_graminic_4/1-242      110 PIIDYLAPCGDDCS---TVDKSSLEFFKIQEAGLNSG-----KWPTDELIANNNSWAVTV 161
aspergillus_niger_1/1-245        108 PVITYLANCNGSCS---DVDKTALQFFKIDAGGLIDDSEIPGTWATDKLIDNNYTRSITI 164
aspergillus_kawachii_40/1-245    108 PVITYLANCNGSCA---DVDKTALQFFKIDAGGLIDDSEIPGTWATDKLIDNNYTRSITI 164
aspergillus_tereus_5/1-232       108 PVLNYLAPCNGDCA---QADKSSLEFFKIEAEGLIDGSSPPGQWATDELISNNNTAVVTI 164
3ZUD/1-228                       89  PVINYLAPCNGDCS---TVDKTQLEFFKIAESGLINDDNPPGIWASDNLIAANNSWTVTI 145
emmericella_nidulans_9/1-245     108 PVIDYIASCNGDCT---TVDKTTLEWVKISESGLVDGSSAPGTWASDNLISNNNSWTVTI 164
penicillum_chrysogenum_2/1-245   108 PVINYLANCNGDCS---KVDKKTLKWFKIAESGLIDGSNAPGKWASDELIANNNSASVTI 164
aspergillus_fumingatus_1/1-245   108 PVITYMANCNGDCA---SVDKTTLEFFKIDESGLISDSNVPGTWASDNLIANNNSWTVTV 164
neosartorya_fischeri_1/1-245     108 PVITYMANCNGDCA---SVDKTSLEFFKIDESGLISDSNVPGTWASDNLIANNNSWTVTV 164
aspergillus_clavatus_6/1-241     108 PVITYMANCNGDCA---SVDKSQLEFFKIEEKGLIGS----GHWASDDLIANNNSWTVTV 160
aspergilus_oryzae_7/1-242        107 PVITYLANCNGDCA---TVDKTQLEFFKIDEKGLISGSD--NTWASDNLISSNNSWTVTI 161
aspergillus_favus_5/1-242        107 PVITYLANCNGDCA---TVDKTQLEFFKIDEKGLISGSD--NTWATDNLIASNNSWTVTI 161
aspergillus_tereus_8/1-241       108 PVITYLANCNGDCS---SVDKTSLEFFKIDQKGLIEG----NTWASDQLISNNNSWTVTI 160
aspergillus_niger_2/1-245        108 PVLTYLANCNGDCS---DVTKTDLEFFKIDESGLISDTEVPGTWATDNLISNNNSWTVTI 164
aspergillus_niger_12/1-244       108 PVLTYLANCNGDCS---DVTKTDLEFFKIDESGLISDTEVPGTWATDNLISNNNSWTVTI 164
aspergillus_kawachii_38/1-245    108 PVLTYLANCNGDCS---DVTKTDLEFFKIDESGLISDTEVPGTWATDNLISNNNSWTVTI 164
zea_mys_1/1-245                  108 PVLTYLANCNGDCS---DVTKTDLEFFKIDESGLISDTEVPGTWATDNLISNNNSWTVTI 164
2VTC/1-228                       93  PIVTYVVECSGSCT---TVNKNNLRWVKIQEAGINYNT---QVWAQQDLINQGNKWTVKI 146
TYPE3:NCU07898/1-239             101 PVITYMARCPDTGCQDWTPSASDKVWFKIKEGGREGTS---NVWAATPLMTAPANYEYAI 157

                                                190       200       210       220       230       240
                                     ....|....|....|....|....|....|....|....|....|....|....|....|
hypocrea_rufa_1/1-246            166 PDNLAPGNYVLRHEIIALHSAGQANGAQNYPQCFNIAVSGSGSLQ--PSGVLGTDLYHAT 223
hypocrea_rufa_2/1-246            166 PDNLAPGNYVLRHEIIALHSAGQANGAQNYPQCFNIAVSGSGSLQ--PSGVLGTDLYHAT 223
trichoderma_saturnusporum_1/1-   166 PDDLAPGNYVLRHEIIALHSAGQANGAQNYPQCFNLAVSGSGSLK--PSGVKGTALYHAT 223
hypocrea_orientalis_1/1-246      166 PEDLAPGNYVLRHEIIALHSAGQADGAQNYPQCFNLAVSGSGSLQ--PSGVKGTALYHSD 223
trichoderma_SP_SSL_1/1-246       166 PEDLAPGNYVLRHEIIALHSAGQADGAQNYPQCFNLAVPGSGSLQ--PSGVKGTALYHSD 223
Hypocrea_virens_3/1-246          166 PADLETGNYVLRHELIALHSAGSVDGAQNYPQCFNLAVTGTGSLQ--PTGVLGTKLYQES 223
trichoderma_atroviride_2/1-246   166 PEDLQTGNYVLRHELIALHSAEQADGAQNYPQCFNLAVTGTGSLQ--PSGVLATDLYHET 223
chaetomium_globosum_5/1-220      168 PEGIAPGNYVLRHEIIALHSGGQPNGAQNYPQCFNLQSSSADHRA--RSAITGSA----- 220
TYPE3:NCU05969/1-243             163 PPTIAPGFYVLRHEIIALHSAGQPNGAQNYPQCFNIQVTGSGTEK--PAGVKGTALYKPD 220
aspergillus_tereus_6/1-245       166 PADIAPGDYVLRHEMIALHGAFAEDGAQNYMQCFNLRVTGGGSQS--PAGVPATELYTST 223
aspergillus_kawachii_37/1-247    167 PPSIAAGNYVLRHELIALHGAEEEDGAQNYPQCFNLQVTGTGTAT--PSGVKGTELYTAT 224
aspergillus_tereus_10/1-245      166 PSSIAPGNYVLRHELIALHGAGSANGAQNYPQCFNLQITGSGTVK--PSGVLGTALYSPT 223
neosartorya_fischeri_4/1-247     167 PPTIAPGYYVLRHELIALHGAGSQNGAQNYPQCFNLQITGSGTAQ--PSGVKGTKLYSPT 224
aspergillus_fumingatus_4/1-247   167 PPTIAPGYYVLRHELIALHGAGSQNGAQNYPQCFNLQITGSGTAQ--PSGVKGTELYSPT 224
giberella_zeae_7/1-251           171 PKNIAPGNYVLRHEIIALHNALDKGGAQNYPQCFNLKITGDGSDS--PSGYLGTELYDAA 228
fusarium_oxysporum_3/1-252       172 PENIASGNYVLRHEIIALHNALERGGAQNYPQCFNLRITGDGSDS--PSGYLGTELYDVE 229
nectria_heamatococcuss_1/1-252   172 PSNIAPGNYVLRHEIIALHSGLERNGAQNYPQCFNLKITGSGSDN--PPGYLGTELYDAN 229
```

```
verticilium_albo_atrum_13/1-24    162 PTDIAAGSYVLRHEIIALHSGGQPNGAQFYPQCINLEISGGGSAS--PSGVAGTSLYTET 219
verticillium_dahliae_4/1-241      162 PTDIAAGSYVLRHEIIALHSGGQPNGAQFYPQCINLEISGGGSAS--PSGVAGTSLYTET 219
neurospora_tetrasperma__2/1-24    160 PENIKPGNYVLRHEIIALHSAGQANGAQNYPQCFNLKVEGSGSTV--PAGVAGTELYKAT 217
TYPE3:NCU07760/1-240              160 PESIKPGNYVLRHEIIALHSAGQANGAQNYPQCFNLKVEGSGSTV--PAGVAGTELYKAT 217
podospora_anseria_31/1-244        164 PADLKAGNYVLRHEIIALHGGSSPNGAQAYPQCLNLRIVGNGNNS--PAGVAGTSLYRAN 221
magna_porte_oryzae_16/1-243       163 PASIAPGNYVLRHEIIALHGAPNANGAQAYPQCFNLEITGSGSTQ--PAGVAGTSLYTAN 220
glomerrela_graminic_4/1-242       162 PTSLKPGKYVWRHEIIALHSAGQEDGAQNYPQCLNIEVTGSGSEE--PAGVKGTALYTPK 219
aspergillus_niger_1/1-245         165 PSDIEAGNYVLRHEIIALHGAEDLDGAQNYPQCINLNVTGSGTAT--PSGTLGTALYMDT 222
aspergillus_kawachii_40/1-245     165 PSDIEAGSYVLRHEIIALHGAEDLDGAQNYPQCINLNVTGSGTAT--PSGTLGTALYKDT 222
aspergillus_tereus_5/1-232        165 PASIASGNYVLRHEIIGLHSAGNLNGAQNYPQCINIEITGGGSTK--PSGVSATIFYKNT 222
3ZUD/1-228                        146 PTTIAPGNYVLRHEIIALHSAQNQDGAQNYPQCINLQVTGGGSDN--PAGTLGTALYHDT 203
emmericella_nidulans_9/1-245      165 PSSLAAGGYVLRHEIIALHSAGNENGAQNYPQCVNLEVTGGGSAS--PSGTVGTELYTPT 222
penicillum_chrysogenum_2/1-245    165 PSSIAAGNYVLRHEIIALHSAGQENGAQNYPQCLNLKVTGGGSDV--PEGVVGTELYKPD 222
aspergillus_fumingatus_1/1-245    165 PSSIAAGNYVMRHEIIALHSAGNQNGAQNYPQCINLKVTGGGSDK--PAGTLGTALYKNT 222
neosartorya_fischeri_1/1-245      165 PSSIAAGNYVMRHEIIALHSAGNQNGAQNYPQCINLKVTGGGSDK--PAGTLGTALYKNT 222
aspergillus_clavatus_6/1-241      161 PSSLAAGNYVMRHEIIGLHSAGNANGAQNYPQCINLKVTGGGSDK--PVGTLGTKLYKAT 218
aspergilus_oryzae_7/1-242         162 PSSIAAGNYVMRHEIIALHSAGNKDGAQNYPQCLNFKVTGGGSDK--PEGTLGTALYKDT 219
aspergillus_favus_5/1-242         162 PSSIAAGNYVMRHEIIGLHSAGQKDGAQNYPQCLNFKVTGGGSDK--PEGTLGTALYKDT 219
aspergillus_tereus_8/1-241        161 PSSIASGNYVLRHEIIGLHSAGNKDGAQNYPQCMNLKVTGGGSDK--PAGTLGTALYKDT 218
aspergillus_niger_2/1-245         165 PSTLEAGNYVLRHEIIALHSAENKDGAQNYPQCLNLKVTGSGSST--YSGTKGEALYKDT 222
aspergillus_niger_12/1-244        165 PSTLEAGNYVLRHEIIALHSAENKDGAQNYPQCLNLKVTGSGSST--YSGTKGEALYKDT 222
aspergillus_kawachii_38/1-245     165 PSTLESGNYVLRHEIIALHSAGNKDGAQNYPQCLNLKVTGSGSST--YSGTKGEALYKDT 222
zea_mys_1/1-245                   165 PSTLEAGNYVLRHEIIALHSAENKDGAQNYPQCLNLKVTGSGSST--YSGTKGEALYKDT 222
2VTC/1-228                        147 PSSLRPGNYVFRHELLAAHGASSANGMQNYPQCVNIAVTGSGTKAL-PAGTPATQLYKPT 205
TYPE3:NCU07898/1-239              158 PSCLKPGYYLVRHEIIALHSAYSYPGAQFYPGCHQLQVTGSGTKTPSSGLVSFPGAYKST 217

                                                  250         260
                                      ....|....|....|....|...
hypocrea_rufa_1/1-246             224 DPGVPINIYTSPLNYIIPGPTVV 246
hypocrea_rufa_2/1-246             224 DPGVLINIYTSPLNYIIPGPTVV 246
trichoderma_saturnusporum_1/1-    224 DPGVLINIYTSPLNYIIPGPTVV 246
hypocrea_orientalis_1/1-246       224 DPGVLINIYTSPLAYTIPGPSVV 246
trichoderma_SP_SSL_1/1-246        224 DPGVLINIYTSPLAYTIPGPSVV 246
Hypocrea_virens_3/1-246           224 DPGILFNIYTSPLTYTIPGPTVV 246
trichoderma_atroviride_2/1-246    224 DPGILFNIYTSPLTYIIPGPTVV 246
chaetomium_globosum_5/1-220       220 ----------------------- 220
TYPE3:NCU05969/1-243              221 DAGISVNIYQSLSSYSIPGPALI 243
aspergillus_tereus_6/1-245        224 DPGILVDIYNS-LTYTVPGPSMI 245
aspergillus_kawachii_37/1-247     225 ENGILVNIYSTLTTYTVPGPTAY 247
aspergillus_tereus_10/1-245       224 DPGILVNIYNS-LNYIVPGPTPI 245
neosartorya_fischeri_4/1-247      225 DPGILVNIYNSLSTYIVPGPTLI 247
aspergillus_fumingatus_4/1-247    225 DPGILVNIYNALSTYIVPGPTLI 247
giberella_zeae_7/1-251            229 DPGILVNVYSSSVDYEVPGPTIC 251
fusarium_oxysporum_3/1-252        230 DPGILVNVYSSSVDYEVPGPTIV 252
nectria_heamatococcuss_1/1-252    230 DPGILVNIYGNLPNYQVPGPTIV 252
verticilium_albo_atrum_13/1-24    220 DPGVLFNIYTA-TEYPIPGPPLY 241
verticillium_dahliae_4/1-241      220 DPGVLFNIYTA-TEYPIPGPPLY 241
neurospora_tetrasperma__2/1-24    218 DAGILFDIYKNDISYPVPGPSLI 240
TYPE3:NCU07760/1-240              218 DAGILFDIYKNDISYPVPGPSLI 240
podospora_anseria_31/1-244        222 DAGILFNPYVASPNYPVPGPALI 244
magna_porte_oryzae_16/1-243       221 DPGILFQLYQAPISYRVPGPPMM 243
glomerrela_graminic_4/1-242       220 DAGILVSVYNNLSKYQIPGPALP 242
aspergillus_niger_1/1-245         223 DPGIYIDIWQSISSYTIPGPTLY 245
aspergillus_kawachii_40/1-245     223 DPGIYIDIWQSISSYTIPGPTLY 245
aspergillus_tereus_5/1-232        223 DPGIKFR-------------HLL 232
3ZUD/1-228                        204 DPGILINIYQKLSSYIIPGPPLY 226
emmericella_nidulans_9/1-245      223 DPGILVNIYTSLDSYTIPGPALW 245
penicillum_chrysogenum_2/1-245    223 DAGILVSIYNQLKDYTIPGPALY 245
aspergillus_fumingatus_1/1-245    223 DAGILVNIYQSLSSYEIPGPALY 245
neosartorya_fischeri_1/1-245      223 DAGILVNIYQSLSSYDIPGPALY 245
aspergillus_clavatus_6/1-241      219 DPGILVNIYGSLTSYTIPGPALW 241
aspergilus_oryzae_7/1-242         220 DPGIEINIYQTLSSYTIPGPALY 242
aspergillus_favus_5/1-242         220 DPGIQINIYQTLTSYTIPGPALY 242
aspergillus_tereus_8/1-241        219 DPGILVNIYQTLSSYVIPGPALY 241
aspergillus_niger_2/1-245         223 DPGILVNIYETLSSYDIPGPAMY 245
aspergillus_niger_12/1-244        223 DPGILVNIYETLSSYDIPGPAI- 244
aspergillus_kawachii_38/1-245     223 DPGILVNIYQSLSSYDIPGPAMY 245
zea_mys_1/1-245                   223 DPGILVNIYETLSSYDIPGPAMY 245
2VTC/1-228                        206 DPGILFNPYTTITSYTIPGPALW 228
TYPE3:NCU07898/1-239              218 DPGVTYDAYQA-ATYTIPGPAVF 239
```
