# Supplementary material for: Bioinformatic characterization of type-specific sequence and structural features in auxiliary activity family 9 proteins
Source: Biotechnol Biofuels. 2016 Nov 9;9:239. doi: 10.1186/s13068-016-0655-2 (PMC5101804; doi:10.1186/s13068-016-0655-2)
Supplement: Supplementary file 6 — Additional file 6. Motifs identified on AA9 domain sequences. The regular expression describes the sequence of the motif, with residues in square bracket showing various amino acids that can occur in that position. The “width” column indicates the length of the motif and the “sites” column displays the number of times the motif occurs in the input dataset. [file 13068_2016_655_MOESM6_ESM.pdf]

Motif	Regular expression	Width	Sites	E-value
Motif 1	**IPS[CD][IL][AP][PS]G[NQD]Y[LV]LR[AHV]E[HI]I[AG]LHS**	21	152	8.0e-2189
Motif 2	**GAQ[FN]Y[PM][SQ]CA[QN][LI][KN]VTG[GS]GS[AG][ST]P**	21	151	2.0e-1787
Motif 3	**V[SK][FG][PT]G[AL]Y[KS]A[TS]DPGIL[IFV][NS]I**	18	148	1.7e-1350
Motif 4	**[IW]PxSH[PK]GPV[IL]VY[LM][AS]K[VCA][PN][GD][AD][AC]T**	21	153	9.5e-1395
Motif 5	**[LA][LA][AL][LA]AAL[VA][SA][AG]H[YG][IT][FV][QS]x[LI]VV[NDG]G**	21	143	2.6e-993
Motif 6	**[FY]PY[EMN][SP][DN]PPEV[IV][GA]W[ST][TA]TATD[NL]GFV[SD][PG][DS]AYG[SD]PDIICH[KR][NS]A[TK][NP]**	41	41	1.1e-951
Motif 7	**[KG][TG][GS][LG]E[WF]FK[IV][YD]EDGLx**	15	151	2.1e-887
Motif 8	**[YW][PN]x[PL][TK]SYT[IV]PGPAV[WF]**	15	142	1.9e-755
Motif 9	**N[GS]P[VI][TE]DVTS[NP]D[IL][RA]CN**	15	108	2.1e-637
Motif 10	**TA[GP]S[ST]G[TV][IL][TE]V[KA]AG[SD][TK][VI][TG][FA]K[WL]x**	21	110	5.7e-564
Motif 11	**W[AG][TSV]DxLI[AN]NN[GN]**	11	90	3.2e-348
Motif 12	**[GI][HS]AxV[KA]AG[DG][TK][VI]E[LF]QW**	15	41	9.1e-2220
Motif 13	**K[SN]K[DGP]F[VKT]D[DT][AD]WC[TS]W[DQG]KD[RT][ILV]EF[TK]**	21	22	1.9e-212
Motif 14	**[DE]W[EQ]Y[VI]RK[NT]T[NR]x**	11	53	1.80E-184
Motif 15	**H[VE]IGG[AP]Q[GF]P[NG]DPDNP**	15	18	2.20E-122
Motif 16	**[DN]QG[QL][LG][KT]G[VLI]R[AV]P**	11	27	1.10E-97
Motif 17	**Q[PN]GDRSC[ST][NQ][EP]A**	11	16	5.40E-77
Motif 18	**MKLSLL**	6	80	2.50E-66
Motif 19	**[QS][LI]TWPSNGK[TS][ESQ][VF]S[VF][TKP]**	15	17	6.10E-66
Motif 20	**GDDD[YF]WG[TV]KDLN[AST]CCGKM[ND]VK**	21	5	4.80E-50
Motif 21	**AKYNPTKW**	8	12	5.50E-45
Motif 22	**VYY[QG]GY[DL][VP]**	8	39	6.50E-38
Motif 23	**MS[VL][AS]KIAG**	8	15	1.20E-28

Motif 24	**YMLSTTG[DS][SA]P[AN]**	11	9	7.70E-26
Motif 25	**D[KY][AG][SG][QT]C[VA]R[TL][PV][AK]**	11	12	1.40E-24
Motif 26	**M[IR][QH][KV][LQ]S[NT][LQ]L[LV][TA]**	11	10	9.10E-22
Motif 27	**[SD]D[ST][EN][VI]PGT**	8	9	1.20E-07
Motif 28	**FPGG[EG]WTLSDTYTFT**	15	3	6.60E-06
Motif 29	**HTL[DT]S[TK]P[AES][NT]D[AK]**	11	5	7.00E-05
Motif 30	**FGSQLTWL[SA]AA[NS]Y[ND]I**	15	3	4.80E-03
