# Supplementary material for: Bioinformatic characterization of type-specific sequence and structural features in auxiliary activity family 9 proteins
Source: Biotechnol Biofuels. 2016 Nov 9;9:239. doi: 10.1186/s13068-016-0655-2 (PMC5101804; doi:10.1186/s13068-016-0655-2)
Supplement: Supplementary file 7 — Additional file 7. Amino acid composition values for all AA9 domains evaluated. [file 13068_2016_655_MOESM7_ESM.pdf]

Sequence name	A	C	D	E	F	G	H	I	K	L	M	N	P	Q	R	S	T	V	W	Y
a_fuminga_3	0.084	0.017	0.076	0.042	0.059	0.097	0.025	0.034	0.084	0.050	0.029	0.038	0.076	0.017	0.029	0.046	0.071	0.059	0.025	0.042
n_fischer_7	0.088	0.017	0.067	0.042	0.059	0.097	0.025	0.034	0.088	0.055	0.025	0.055	0.067	0.021	0.025	0.050	0.059	0.059	0.025	0.042
a_clavatus_1	0.101	0.017	0.055	0.046	0.059	0.092	0.025	0.038	0.084	0.046	0.021	0.050	0.071	0.021	0.029	0.046	0.063	0.071	0.021	0.042
p_chrysoge_4	0.102	0.017	0.068	0.055	0.055	0.093	0.021	0.038	0.085	0.025	0.034	0.034	0.076	0.017	0.025	0.051	0.068	0.068	0.025	0.042
a_oryzae_5	0.092	0.017	0.071	0.042	0.050	0.101	0.021	0.029	0.092	0.038	0.029	0.029	0.071	0.021	0.025	0.055	0.067	0.067	0.021	0.059
a_favus_3	0.092	0.017	0.071	0.042	0.050	0.101	0.021	0.029	0.092	0.038	0.029	0.029	0.071	0.021	0.025	0.055	0.067	0.067	0.021	0.059
a_niger_9	0.071	0.017	0.080	0.042	0.046	0.113	0.025	0.042	0.084	0.046	0.034	0.034	0.080	0.029	0.025	0.038	0.080	0.046	0.025	0.042
e_nidulans_6	0.084	0.017	0.088	0.046	0.046	0.105	0.025	0.042	0.067	0.038	0.038	0.038	0.071	0.038	0.025	0.042	0.071	0.050	0.021	0.046
v_albo_atrum_17	0.104	0.009	0.052	0.052	0.062	0.104	0.024	0.033	0.071	0.038	0.024	0.043	0.076	0.019	0.033	0.043	0.071	0.081	0.014	0.047
v_dahiae_25	0.100	0.014	0.062	0.052	0.066	0.090	0.024	0.033	0.076	0.038	0.024	0.033	0.071	0.019	0.028	0.062	0.076	0.071	0.014	0.047
a_clavatu_5	0.080	0.017	0.050	0.034	0.063	0.092	0.025	0.046	0.097	0.029	0.025	0.067	0.076	0.021	0.029	0.055	0.046	0.076	0.017	0.055
c_higginsianum_24	0.097	0.017	0.046	0.042	0.063	0.113	0.021	0.034	0.092	0.029	0.025	0.046	0.067	0.017	0.021	0.050	0.067	0.080	0.021	0.050
g_graminic_7	0.097	0.017	0.063	0.042	0.067	0.105	0.021	0.038	0.076	0.025	0.029	0.050	0.063	0.017	0.029	0.055	0.071	0.063	0.021	0.050
n_fischer_2	0.051	0.017	0.055	0.047	0.047	0.098	0.026	0.051	0.064	0.064	0.021	0.030	0.081	0.030	0.017	0.094	0.068	0.068	0.021	0.051
a_fuminga_6	0.060	0.017	0.055	0.043	0.047	0.094	0.026	0.047	0.064	0.064	0.021	0.034	0.081	0.034	0.026	0.085	0.068	0.064	0.021	0.051
a_tereus_12	0.060	0.017	0.072	0.034	0.051	0.106	0.021	0.047	0.051	0.060	0.026	0.030	0.072	0.026	0.021	0.098	0.055	0.077	0.017	0.060
a_favus_7	0.055	0.017	0.068	0.043	0.047	0.102	0.021	0.055	0.068	0.060	0.021	0.034	0.077	0.030	0.017	0.085	0.055	0.068	0.017	0.060
p_anseria_17	0.074	0.017	0.030	0.048	0.043	0.121	0.022	0.069	0.043	0.056	0.013	0.074	0.078	0.030	0.056	0.043	0.052	0.052	0.022	0.056
c_globusum_15	0.092	0.017	0.044	0.044	0.052	0.118	0.017	0.048	0.061	0.048	0.031	0.066	0.083	0.022	0.035	0.048	0.039	0.061	0.017	0.057
g_graminic_16	0.052	0.022	0.030	0.039	0.043	0.113	0.026	0.061	0.057	0.048	0.022	0.057	0.074	0.035	0.026	0.065	0.070	0.074	0.026	0.061
g_graminic_31	0.083	0.017	0.048	0.039	0.048	0.113	0.026	0.048	0.057	0.061	0.030	0.043	0.078	0.022	0.017	0.065	0.070	0.061	0.026	0.048
P_nodo_4	0.100	0.009	0.043	0.043	0.035	0.100	0.022	0.061	0.091	0.057	0.022	0.043	0.065	0.030	0.017	0.052	0.074	0.061	0.022	0.052
p_teres_22	0.078	0.017	0.030	0.048	0.043	0.096	0.022	0.048	0.078	0.057	0.022	0.057	0.065	0.039	0.017	0.083	0.048	0.083	0.026	0.043
p_trici_repentis_23	0.072	0.019	0.034	0.053	0.034	0.096	0.024	0.053	0.072	0.067	0.019	0.053	0.077	0.034	0.019	0.067	0.053	0.087	0.029	0.038
p_anseria_5	0.063	0.014	0.036	0.014	0.045	0.104	0.023	0.059	0.045	0.045	0.014	0.077	0.072	0.045	0.023	0.113	0.059	0.081	0.014	0.054
m_thermophilia_2	0.090	0.014	0.059	0.009	0.041	0.113	0.023	0.059	0.032	0.050	0.009	0.050	0.072	0.041	0.018	0.077	0.108	0.063	0.018	0.059
t_terestis_7	0.090	0.013	0.067	0.004	0.049	0.112	0.018	0.067	0.031	0.049	0.013	0.063	0.076	0.049	0.013	0.058	0.094	0.049	0.018	0.063
s_macrospora_4	0.092	0.014	0.037	0.018	0.046	0.111	0.023	0.060	0.032	0.055	0.005	0.041	0.074	0.032	0.014	0.106	0.111	0.055	0.018	0.055
n_tetrasperma_12	0.092	0.014	0.041	0.028	0.041	0.101	0.023	0.050	0.041	0.055	0.005	0.041	0.064	0.032	0.009	0.115	0.087	0.083	0.023	0.055
TYPE1:NCU00836	0.092	0.014	0.046	0.028	0.041	0.101	0.023	0.050	0.041	0.055	0.005	0.041	0.064	0.032	0.009	0.110	0.087	0.083	0.023	0.055
s_commune_15	0.114	0.009	0.039	0.053	0.022	0.101	0.018	0.053	0.031	0.053	0.013	0.057	0.083	0.031	0.018	0.061	0.088	0.088	0.013	0.057
s_commune_16	0.110	0.009	0.031	0.026	0.026	0.096	0.022	0.066	0.022	0.053	0.018	0.057	0.083	0.026	0.018	0.110	0.083	0.075	0.013	0.057
s_lacrymans_5	0.100	0.009	0.031	0.022	0.017	0.100	0.017	0.066	0.013	0.061	0.013	0.052	0.066	0.035	0.017	0.100	0.092	0.109	0.022	0.057
4b5q	0.069	0.009	0.032	0.055	0.046	0.111	0.018	0.051	0.023	0.055	0.009	0.055	0.088	0.065	0.014	0.060	0.092	0.069	0.014	0.065
e_nidulan_3	0.092	0.009	0.083	0.031	0.035	0.131	0.017	0.039	0.031	0.074	0.009	0.044	0.087	0.017	0.017	0.052	0.092	0.070	0.026	0.044
a_tereus__4	0.101	0.009	0.066	0.035	0.035	0.123	0.018	0.044	0.031	0.066	0.013	0.026	0.088	0.018	0.013	0.057	0.110	0.070	0.022	0.057
v_dahiae_24	0.081	0.009	0.045	0.036	0.059	0.081	0.014	0.045	0.027	0.072	0.014	0.041	0.099	0.036	0.045	0.090	0.068	0.081	0.014	0.045
v_albo_atrum_16	0.083	0.009	0.046	0.037	0.065	0.083	0.014	0.037	0.018	0.074	0.009	0.055	0.106	0.037	0.046	0.074	0.055	0.088	0.018	0.046
v_dahiae_26	0.072	0.009	0.041	0.072	0.050	0.109	0.023	0.032	0.023	0.072	0.018	0.041	0.104	0.023	0.032	0.072	0.063	0.081	0.018	0.045
p_trici_repentis_20	0.105	0.009	0.022	0.031	0.026	0.118	0.018	0.031	0.057	0.070	0.009	0.035	0.075	0.031	0.013	0.101	0.101	0.066	0.022	0.061
P_nodorum_28	0.097	0.009	0.022	0.022	0.053	0.111	0.018	0.031	0.049	0.053	0.013	0.031	0.080	0.031	0.013	0.111	0.111	0.080	0.013	0.053
t_terestis_11	0.100	0.013	0.030	0.030	0.035	0.108	0.026	0.052	0.022	0.069	0.009	0.030	0.087	0.056	0.013	0.069	0.126	0.065	0.017	0.043
c_thermophilia_14	0.078	0.013	0.026	0.026	0.039	0.113	0.035	0.043	0.039	0.074	0.013	0.039	0.091	0.048	0.013	0.056	0.113	0.082	0.017	0.043
p_trici_repentis_13	0.109	0.014	0.032	0.018	0.032	0.100	0.023	0.064	0.036	0.059	0.009	0.068	0.082	0.041	0.027	0.082	0.077	0.059	0.023	0.045
m_thermophilia_16	0.096	0.013	0.030	0.039	0.035	0.122	0.026	0.043	0.026	0.065	0.017	0.017	0.078	0.052	0.013	0.096	0.091	0.078	0.017	0.043
p_anseria_11	0.100	0.013	0.030	0.017	0.030	0.126	0.026	0.035	0.043	0.065	0.013	0.035	0.082	0.048	0.013	0.078	0.100	0.082	0.017	0.048
c_globusum_24	0.080	0.013	0.040	0.027	0.035	0.115	0.027	0.044	0.058	0.062	0.009	0.027	0.084	0.031	0.009	0.088	0.102	0.093	0.018	0.040
TYPE1ncr:NCU02344	0.116	0.013	0.022	0.043	0.039	0.134	0.030	0.026	0.047	0.078	0.022	0.039	0.069	0.022	0.013	0.073	0.082	0.065	0.009	0.060
a_oligospora_11	0.101	0.016	0.037	0.016	0.032	0.106	0.021	0.069	0.037	0.058	0.011	0.037	0.090	0.026	0.026	0.101	0.085	0.063	0.026	0.042
g_graminic_6	0.130	0.013	0.026	0.030	0.039	0.109	0.022	0.030	0.039	0.078	0.009	0.030	0.070	0.035	0.022	0.091	0.083	0.065	0.026	0.052
p_teres_11	0.120	0.010	0.033	0.014	0.033	0.086	0.024	0.057	0.038	0.067	0.010	0.067	0.077	0.038	0.029	0.100	0.067	0.057	0.024	0.048
P_nodorum_18	0.119	0.014	0.041	0.014	0.032	0.087	0.023	0.060	0.050	0.060	0.014	0.069	0.078	0.041	0.018	0.083	0.069	0.060	0.023	0.046
t_terestis_18	0.103	0.013	0.049	0.018	0.040	0.103	0.018	0.045	0.022	0.045	0.009	0.049	0.098	0.054	0.022	0.071	0.063	0.089	0.031	0.058
3eja	0.087	0.019	0.053	0.019	0.038	0.096	0.019	0.043	0.024	0.034	0.005	0.048	0.106	0.058	0.024	0.077	0.067	0.087	0.034	0.063
p_anseria_18	0.090	0.013	0.027	0.013	0.040	0.103	0.027	0.049	0.013	0.058	0.009	0.063	0.085	0.063	0.045	0.099	0.054	0.067	0.027	0.054
TYPE1:NCU03328	0.096	0.013	0.026	0.017	0.044	0.092	0.026	0.057	0.035	0.057	0.009	0.052	0.074	0.057	0.022	0.122	0.066	0.057	0.026	0.052

c_globusum_8	0.090	0.013	0.040	0.018	0.045	0.108	0.027	0.049	0.040	0.049	0.009	0.054	0.072	0.058	0.022	0.112	0.049	0.072	0.027	0.045
m_thermophilia_21	0.094	0.013	0.031	0.031	0.045	0.099	0.027	0.040	0.022	0.054	0.009	0.045	0.076	0.081	0.031	0.076	0.072	0.081	0.022	0.049
c_globusum_23	0.094	0.012	0.053	0.012	0.029	0.111	0.016	0.045	0.045	0.078	0.012	0.066	0.061	0.066	0.020	0.111	0.037	0.078	0.020	0.033
m_thermophilia_5	0.102	0.012	0.045	0.016	0.037	0.102	0.016	0.061	0.041	0.061	0.012	0.090	0.066	0.061	0.012	0.082	0.057	0.066	0.020	0.037
t_terestis_2	0.078	0.012	0.045	0.008	0.033	0.107	0.025	0.057	0.041	0.074	0.012	0.066	0.061	0.086	0.016	0.115	0.033	0.074	0.020	0.037
c_thermophilia_15	0.082	0.012	0.049	0.012	0.037	0.131	0.029	0.066	0.029	0.070	0.012	0.061	0.061	0.070	0.037	0.082	0.041	0.057	0.020	0.041
p_anseria_29	0.098	0.016	0.041	0.008	0.045	0.115	0.029	0.070	0.045	0.049	0.008	0.082	0.066	0.090	0.020	0.070	0.041	0.057	0.016	0.033
s_macrospora_16	0.086	0.012	0.049	0.008	0.033	0.110	0.016	0.069	0.053	0.061	0.016	0.082	0.069	0.065	0.016	0.098	0.041	0.057	0.020	0.037
P_indica_13	0.112	0.012	0.037	0.012	0.033	0.124	0.017	0.066	0.037	0.058	0.017	0.058	0.062	0.046	0.021	0.141	0.041	0.054	0.021	0.029
P_indica_12	0.112	0.012	0.037	0.012	0.033	0.120	0.017	0.062	0.050	0.062	0.012	0.066	0.066	0.050	0.008	0.124	0.046	0.054	0.021	0.033
g_graminic_17	0.115	0.012	0.045	0.016	0.037	0.127	0.020	0.066	0.037	0.053	0.012	0.037	0.074	0.053	0.029	0.098	0.041	0.070	0.025	0.033
v_dahiae_23	0.107	0.012	0.058	0.058	0.029	0.099	0.025	0.070	0.025	0.074	0.008	0.058	0.074	0.029	0.029	0.053	0.058	0.074	0.021	0.041
v_albo_atrum15	0.110	0.010	0.057	0.067	0.043	0.110	0.024	0.062	0.029	0.057	0.010	0.048	0.071	0.038	0.038	0.057	0.048	0.052	0.029	0.043
m_thermophilia_6	0.116	0.012	0.046	0.017	0.029	0.112	0.025	0.058	0.033	0.058	0.017	0.079	0.079	0.046	0.029	0.091	0.021	0.079	0.021	0.033
c_globusum_31	0.120	0.012	0.033	0.025	0.029	0.104	0.025	0.079	0.033	0.037	0.021	0.083	0.066	0.058	0.025	0.083	0.037	0.071	0.021	0.037
p_anseria_4	0.116	0.012	0.037	0.025	0.037	0.112	0.037	0.054	0.029	0.058	0.021	0.075	0.071	0.041	0.029	0.066	0.041	0.083	0.021	0.033
s_macrospora_2	0.133	0.013	0.042	0.013	0.029	0.138	0.021	0.071	0.042	0.050	0.013	0.075	0.063	0.038	0.013	0.079	0.050	0.071	0.021	0.029
TYIPE2:NCU02916	0.141	0.012	0.050	0.012	0.029	0.129	0.021	0.071	0.050	0.054	0.025	0.071	0.058	0.033	0.008	0.079	0.050	0.062	0.017	0.029
n_tetrasperma_16	0.141	0.012	0.050	0.012	0.025	0.129	0.021	0.075	0.046	0.054	0.021	0.071	0.058	0.033	0.008	0.079	0.054	0.062	0.021	0.029
n_tetrasperma_14	0.129	0.012	0.050	0.012	0.025	0.129	0.021	0.079	0.050	0.050	0.021	0.071	0.058	0.033	0.008	0.083	0.058	0.062	0.021	0.029
l_maculas_15	0.085	0.011	0.068	0.034	0.034	0.102	0.028	0.068	0.080	0.080	0.023	0.034	0.102	0.017	0.017	0.051	0.051	0.040	0.023	0.051
b_fuckelina_2	0.094	0.013	0.043	0.021	0.017	0.094	0.013	0.077	0.026	0.056	0.021	0.056	0.081	0.034	0.017	0.081	0.124	0.068	0.013	0.051
b_fuckelina_12	0.079	0.013	0.033	0.017	0.025	0.100	0.017	0.083	0.021	0.071	0.021	0.063	0.071	0.046	0.013	0.083	0.121	0.058	0.013	0.054
s_sclerot_5	0.083	0.013	0.042	0.017	0.025	0.092	0.017	0.075	0.021	0.079	0.017	0.054	0.075	0.050	0.013	0.096	0.108	0.058	0.013	0.054
g_lozoyensis_6	0.092	0.013	0.050	0.013	0.025	0.096	0.021	0.079	0.046	0.067	0.008	0.042	0.079	0.042	0.013	0.100	0.096	0.058	0.013	0.050
P_nodo_9	0.088	0.013	0.062	0.013	0.035	0.115	0.018	0.066	0.057	0.053	0.018	0.044	0.053	0.031	0.018	0.110	0.079	0.079	0.013	0.035
TYPE2:NCU02240	0.106	0.013	0.060	0.017	0.017	0.111	0.017	0.081	0.038	0.068	0.021	0.038	0.064	0.030	0.013	0.085	0.111	0.047	0.013	0.051
g_graminic_9	0.091	0.017	0.056	0.017	0.022	0.108	0.022	0.069	0.052	0.052	0.017	0.052	0.073	0.039	0.022	0.060	0.099	0.073	0.013	0.047
c_thermophilia_18	0.072	0.013	0.038	0.038	0.026	0.119	0.021	0.055	0.051	0.051	0.026	0.068	0.085	0.030	0.021	0.068	0.072	0.089	0.021	0.034
m_thermophilia_10	0.077	0.013	0.030	0.034	0.021	0.132	0.017	0.068	0.043	0.051	0.026	0.060	0.081	0.034	0.026	0.068	0.085	0.081	0.017	0.038
p_anseria_30	0.090	0.013	0.026	0.026	0.026	0.128	0.026	0.073	0.043	0.043	0.026	0.064	0.077	0.034	0.017	0.085	0.068	0.077	0.017	0.043
c_higginsianum_23_12	0.068	0.013	0.034	0.026	0.026	0.128	0.017	0.068	0.043	0.051	0.021	0.051	0.077	0.043	0.017	0.094	0.085	0.085	0.021	0.034
s_macrospora_11	0.093	0.013	0.038	0.025	0.021	0.123	0.013	0.059	0.042	0.047	0.025	0.051	0.068	0.038	0.021	0.089	0.081	0.097	0.017	0.038
n_tetrasperma_1	0.102	0.013	0.034	0.030	0.021	0.110	0.017	0.064	0.042	0.047	0.025	0.047	0.068	0.030	0.021	0.106	0.072	0.097	0.017	0.038
4eir	0.090	0.018	0.036	0.031	0.022	0.117	0.018	0.063	0.040	0.036	0.022	0.049	0.067	0.031	0.022	0.099	0.085	0.094	0.018	0.040
n_cassa_1	0.102	0.013	0.034	0.030	0.021	0.110	0.017	0.059	0.042	0.047	0.025	0.047	0.068	0.030	0.021	0.106	0.076	0.097	0.017	0.038
TYPE2:NCU01050	0.106	0.013	0.035	0.031	0.018	0.111	0.018	0.062	0.044	0.049	0.027	0.049	0.062	0.031	0.022	0.102	0.075	0.093	0.018	0.035
p_trici_repentis_22	0.102	0.014	0.074	0.028	0.037	0.097	0.023	0.046	0.056	0.079	0.019	0.051	0.079	0.032	0.028	0.060	0.065	0.065	0.014	0.032
P_indica_8	0.095	0.024	0.029	0.014	0.029	0.114	0.014	0.033	0.033	0.071	0.024	0.062	0.052	0.033	0.014	0.114	0.067	0.110	0.014	0.052
l_maculans_11	0.139	0.027	0.040	0.027	0.036	0.108	0.018	0.045	0.054	0.054	0.018	0.049	0.049	0.022	0.018	0.099	0.081	0.067	0.013	0.036
P_nodorum_14	0.128	0.026	0.044	0.022	0.035	0.119	0.018	0.022	0.057	0.057	0.018	0.022	0.053	0.031	0.022	0.101	0.075	0.097	0.013	0.040
p_lycope_1	0.145	0.026	0.040	0.026	0.035	0.101	0.022	0.026	0.053	0.053	0.018	0.035	0.048	0.022	0.022	0.128	0.057	0.093	0.022	0.026
p_trici_repentis_11	0.128	0.026	0.035	0.026	0.040	0.106	0.018	0.035	0.053	0.031	0.022	0.031	0.048	0.035	0.022	0.110	0.088	0.088	0.013	0.044
p_teres_25	0.128	0.026	0.040	0.026	0.040	0.101	0.018	0.031	0.053	0.035	0.022	0.031	0.057	0.031	0.022	0.101	0.101	0.079	0.013	0.044
g_lozoyensis_1	0.117	0.023	0.028	0.023	0.019	0.103	0.023	0.056	0.047	0.033	0.023	0.061	0.084	0.037	0.023	0.103	0.079	0.065	0.014	0.037
g_graminic_32	0.151	0.026	0.065	0.034	0.026	0.086	0.026	0.047	0.047	0.043	0.039	0.065	0.052	0.030	0.017	0.065	0.047	0.082	0.009	0.043
p_teres_17	0.137	0.026	0.052	0.013	0.039	0.116	0.017	0.052	0.043	0.047	0.021	0.056	0.052	0.047	0.017	0.090	0.069	0.060	0.013	0.034
p_trici_repentis_8	0.137	0.026	0.047	0.017	0.030	0.116	0.017	0.056	0.043	0.056	0.021	0.060	0.052	0.047	0.017	0.086	0.060	0.060	0.013	0.039
p_anseria_15	0.132	0.030	0.047	0.021	0.026	0.132	0.026	0.030	0.043	0.056	0.017	0.034	0.056	0.038	0.034	0.090	0.068	0.068	0.017	0.034
t_terestis_10	0.136	0.030	0.051	0.021	0.030	0.115	0.030	0.043	0.038	0.038	0.026	0.043	0.055	0.043	0.030	0.085	0.055	0.081	0.021	0.030
p_anseria_24	0.113	0.029	0.063	0.013	0.029	0.117	0.029	0.050	0.038	0.046	0.025	0.046	0.063	0.050	0.033	0.079	0.067	0.067	0.017	0.029
c_thermoph_7	0.121	0.029	0.063	0.029	0.021	0.113	0.025	0.038	0.058	0.054	0.033	0.046	0.075	0.033	0.017	0.054	0.054	0.079	0.021	0.038
t_terestis_17	0.108	0.029	0.075	0.025	0.025	0.092	0.021	0.046	0.038	0.042	0.029	0.038	0.063	0.033	0.021	0.100	0.067	0.088	0.021	0.042
m_thermophilia_12	0.138	0.029	0.059	0.017	0.025	0.113	0.021	0.021	0.042	0.059	0.025	0.038	0.071	0.025	0.021	0.100	0.067	0.071	0.017	0.042
c_globusum_22	0.117	0.029	0.063	0.013	0.021	0.138	0.021	0.033	0.046	0.042	0.025	0.033	0.050	0.033	0.021	0.105	0.059	0.092	0.017	0.042
h_rufa_1	0.081	0.016	0.065	0.016	0.024	0.093	0.033	0.077	0.028	0.073	0.004	0.081	0.085	0.033	0.008	0.053	0.065	0.098	0.024	0.041
h_rufa_2	0.081	0.016	0.065	0.016	0.024	0.098	0.033	0.077	0.028	0.077	0.004	0.081	0.081	0.033	0.004	0.053	0.065	0.098	0.024	0.041

t_saturnusporum_1	0.089	0.016	0.061	0.016	0.024	0.102	0.033	0.073	0.033	0.077	0.004	0.081	0.081	0.024	0.008	0.061	0.053	0.098	0.020	0.045
h_orientalis_1	0.085	0.016	0.069	0.020	0.024	0.102	0.033	0.069	0.033	0.077	0.004	0.069	0.081	0.033	0.004	0.069	0.049	0.098	0.020	0.045
t_SP_SSL_1	0.085	0.016	0.069	0.020	0.020	0.102	0.033	0.069	0.033	0.077	0.004	0.069	0.089	0.033	0.004	0.065	0.049	0.098	0.020	0.045
H_virens_3	0.069	0.016	0.053	0.024	0.024	0.110	0.028	0.069	0.016	0.085	0.008	0.065	0.081	0.037	0.016	0.053	0.089	0.089	0.020	0.045
t_atroviride_2	0.069	0.016	0.061	0.024	0.033	0.102	0.033	0.057	0.028	0.077	0.008	0.069	0.077	0.041	0.004	0.049	0.085	0.102	0.020	0.045
c_globusum_5	0.118	0.018	0.055	0.036	0.041	0.109	0.041	0.059	0.023	0.045	0.009	0.073	0.064	0.027	0.014	0.091	0.055	0.073	0.023	0.027
type3	0.103	0.016	0.053	0.037	0.033	0.099	0.029	0.074	0.053	0.058	0.008	0.066	0.066	0.037	0.008	0.091	0.037	0.074	0.021	0.037
a_tereus_6	0.110	0.016	0.073	0.024	0.033	0.098	0.029	0.057	0.016	0.078	0.024	0.057	0.069	0.037	0.012	0.057	0.078	0.073	0.024	0.033
a_kawachi_37	0.097	0.016	0.057	0.057	0.024	0.089	0.032	0.053	0.020	0.069	0.008	0.065	0.061	0.036	0.016	0.057	0.093	0.085	0.020	0.045
a_tereus_10	0.082	0.016	0.057	0.016	0.020	0.110	0.037	0.082	0.016	0.094	0.004	0.082	0.073	0.037	0.016	0.057	0.073	0.065	0.024	0.037
n_fischer_4	0.073	0.016	0.057	0.016	0.020	0.097	0.040	0.073	0.020	0.073	0.004	0.069	0.077	0.040	0.020	0.065	0.089	0.085	0.024	0.040
a_fuminga_4	0.081	0.016	0.061	0.020	0.016	0.097	0.036	0.077	0.016	0.077	0.004	0.069	0.077	0.040	0.024	0.061	0.081	0.081	0.024	0.040
g_zeae_7	0.072	0.020	0.084	0.040	0.028	0.104	0.028	0.068	0.040	0.088	0.008	0.060	0.072	0.032	0.028	0.064	0.044	0.056	0.020	0.048
f_oxysporum_3	0.063	0.024	0.063	0.071	0.024	0.103	0.028	0.071	0.016	0.079	0.012	0.056	0.067	0.028	0.040	0.063	0.048	0.075	0.020	0.048
n_heamatoccocuss_1	0.067	0.020	0.056	0.032	0.032	0.099	0.032	0.067	0.032	0.075	0.016	0.063	0.075	0.036	0.036	0.063	0.060	0.067	0.020	0.052
v_albo_atrum_13	0.120	0.017	0.054	0.046	0.041	0.100	0.025	0.071	0.025	0.066	0.004	0.050	0.091	0.021	0.012	0.062	0.079	0.054	0.021	0.041
v_dahiae_4	0.120	0.017	0.054	0.046	0.037	0.100	0.025	0.066	0.025	0.066	0.004	0.046	0.091	0.021	0.012	0.066	0.079	0.062	0.021	0.041
n_tetrasperma_2	0.104	0.017	0.079	0.033	0.029	0.100	0.029	0.075	0.063	0.054	0.004	0.054	0.063	0.029	0.008	0.075	0.046	0.075	0.021	0.042
type3:NCU07760	0.104	0.017	0.079	0.033	0.029	0.100	0.029	0.075	0.063	0.054	0.008	0.050	0.063	0.029	0.008	0.079	0.046	0.071	0.021	0.042
p_anseria_31	0.119	0.016	0.041	0.020	0.020	0.107	0.033	0.066	0.025	0.070	0.004	0.094	0.082	0.029	0.025	0.061	0.053	0.066	0.025	0.045
m_porte_oryzae_16	0.103	0.016	0.041	0.029	0.029	0.111	0.033	0.066	0.029	0.062	0.012	0.045	0.082	0.049	0.029	0.070	0.053	0.066	0.025	0.049
g_graminic_4	0.112	0.017	0.058	0.045	0.029	0.087	0.025	0.050	0.054	0.062	0.017	0.050	0.062	0.029	0.008	0.091	0.062	0.079	0.025	0.041
a_niger_1	0.082	0.016	0.086	0.029	0.008	0.102	0.024	0.082	0.020	0.073	0.012	0.045	0.065	0.020	0.012	0.086	0.098	0.053	0.020	0.065
a_kawachii_40	0.086	0.016	0.082	0.037	0.008	0.102	0.024	0.082	0.024	0.073	0.008	0.041	0.061	0.020	0.012	0.090	0.094	0.053	0.020	0.065
a_tereus_5	0.095	0.017	0.052	0.039	0.022	0.116	0.030	0.078	0.043	0.069	0.013	0.056	0.056	0.022	0.009	0.091	0.078	0.052	0.017	0.047
3zud	0.071	0.018	0.062	0.022	0.018	0.106	0.031	0.088	0.022	0.071	0.004	0.075	0.093	0.049	0.009	0.053	0.080	0.053	0.022	0.053
e_nidulan_9	0.110	0.016	0.057	0.041	0.008	0.122	0.024	0.057	0.012	0.061	0.004	0.053	0.053	0.020	0.008	0.094	0.094	0.082	0.029	0.053
p_chrysoge_2	0.098	0.016	0.057	0.053	0.012	0.122	0.033	0.065	0.053	0.069	0.012	0.065	0.053	0.020	0.004	0.082	0.041	0.073	0.020	0.049
a_fuminga_1	0.102	0.016	0.053	0.037	0.020	0.110	0.024	0.069	0.045	0.061	0.016	0.065	0.049	0.024	0.004	0.102	0.061	0.065	0.020	0.053
n_fischer_1	0.098	0.016	0.049	0.037	0.020	0.110	0.024	0.069	0.045	0.061	0.016	0.065	0.045	0.024	0.004	0.118	0.053	0.069	0.020	0.053
a_clavatus_6	0.091	0.017	0.046	0.029	0.021	0.133	0.033	0.066	0.054	0.066	0.017	0.062	0.050	0.021	0.004	0.079	0.075	0.066	0.025	0.046
a_oryzae_7	0.103	0.017	0.066	0.033	0.017	0.107	0.025	0.079	0.050	0.058	0.017	0.058	0.041	0.033	0.004	0.095	0.062	0.058	0.021	0.058
a_favus_5	0.107	0.017	0.070	0.029	0.017	0.107	0.029	0.087	0.054	0.054	0.012	0.054	0.050	0.037	0.004	0.074	0.070	0.054	0.021	0.054
a_tereus_8	0.087	0.017	0.062	0.037	0.012	0.116	0.025	0.066	0.050	0.071	0.008	0.046	0.054	0.025	0.004	0.095	0.079	0.071	0.021	0.054
a_niger_2	0.082	0.016	0.073	0.053	0.012	0.102	0.024	0.065	0.041	0.069	0.008	0.045	0.053	0.012	0.004	0.106	0.082	0.069	0.020	0.061
a_niger_12	0.086	0.016	0.074	0.053	0.012	0.102	0.025	0.070	0.041	0.070	0.004	0.045	0.049	0.012	0.004	0.107	0.082	0.070	0.020	0.057
a_kawachii_38	0.086	0.016	0.069	0.049	0.012	0.106	0.024	0.061	0.041	0.069	0.008	0.045	0.049	0.020	0.004	0.110	0.073	0.073	0.020	0.061
z_mys_1	0.094	0.016	0.069	0.057	0.012	0.102	0.024	0.061	0.041	0.069	0.008	0.045	0.045	0.016	0.004	0.098	0.082	0.073	0.020	0.061
2_chainA	0.088	0.018	0.031	0.018	0.031	0.096	0.026	0.066	0.035	0.053	0.004	0.083	0.079	0.070	0.013	0.048	0.083	0.066	0.031	0.061
t_3:NCU07898	0.117	0.021	0.046	0.021	0.033	0.092	0.025	0.050	0.038	0.050	0.017	0.021	0.092	0.042	0.025	0.079	0.084	0.059	0.021	0.067
